# Desymmetrization of Dicationic Diboranes by Isomerization Catalyzed by a Nucleophile

**DOI:** 10.1002/anie.202001640

**Published:** 2020-04-06

**Authors:** Florian Schön, Lutz Greb, Elisabeth Kaifer, Hans‐Jörg Himmel

**Affiliations:** ^1^ Anorganisch-Chemisches Institut Ruprecht-Karls-Universität Heidelberg Im Neuenheimer Feld 270 69120 Heidelberg Germany

**Keywords:** diborane, dications, electrophilic compounds, guanidine, isomerization

## Abstract

Cationic monoboranes exhibit a rich chemistry. By constrast, only a few cationic diboranes are known, that all are symmetrically substituted. In this work, the first unsymmetrically substituted dicationic diboranes, featuring sp^2^–sp^2^‐hybridized boron atoms, are reported. The compounds are formed by intramolecular rearrangement from preceding isomeric symmetrically substituted dicationic diboranes, a process that is catalyzed by nucleophiles. From the temperature‐dependence of the isomerization rate, activation parameters for this unprecedented rearrangement are derived. The difference in fluoride ion affinity between the two boron atoms and the bonding situation in these unique unsymmetrical dicationic diboranes are evaluated.

## Graphical abstract

**An uneven match**: Isomerization of symmetrically substituted dicationic diboranes, catalyzed by nucleophiles, affords unsymmetrically substituted dicationic diboranes with polarized B−B bonds.

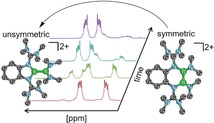

## Introduction

Diboranes with sp^2^ hybridization of the two boron atoms are valuable reagents in modern synthetic chemistry, and they are used in numerous borylation and diboration reactions.^1^ The Lewis acidity of diboranes with weak π‐donor substituents is exceptionally high, and allows, for example, for spontaneous dihydrogen activation with the symmetrically substituted tetra(*o*‐tolyl)‐diborane.^2^ In the diboranes commonly applied for synthesis (for example B_2_cat_2_ or B_2_pin_2_, where cat denotes the catecholate and pin the pinacolate group), Lewis acidity is attenuated by π‐donor substituents.^1^

Unsymmetrically substituted diboranes feature polarized B−B bonds and reveal enhanced reactivity towards various substrates. Thus, the external addition of one equivalent of a Lewis basic co‐reagent to symmetrical diboranes with sp^2^‐hybridized boron atoms is usually required to activate diboranes for further reactions.^3^, ^4^ For example, the addition of a chiral base allows enantioselective borylation reactions.^5^ Base addition turns these compounds into nucleophiles,^4^ especially if anionic activators such as alkoxides are used.^6^ Neutral diboranes that are a priori unsymmetrical have also been reported, for example, pinBBMes_2_ 7 (where Mes denotes the mesityl group).

**Figure 1:**
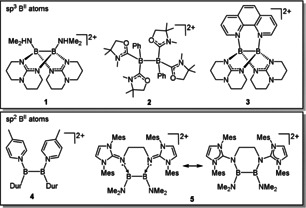
Collection of known, symmetrically substituted, dicationic diboranes with sp^3^ and sp^2^ hybridized boron atoms. Dur=2,3,5,6‐tetramethylphenyl.

Charge is another way to vary the Lewis acidity of monoboranes, as shown in boronium cations with sp^3^‐hybridized boron atoms, borenium cations with sp^2^‐hybridized boron atoms, or even borinium cations with sp‐hybridized boron atoms.^8^ Generally, the Lewis acidity increases with decreasing number of substituents (boronium < borenium < borinium). Consequently, also the rewarding synthesis of cationic diboranes has been achieved in recent years (see Figure 1). In compounds **1**,^9^ **2**,^10^ and **3**,^11^ the two boron atoms are sp^3^‐hybridized. Compound **4**,^12^ isolated in small amounts, and compound **5** 13 are the only known examples of dicationic diboranes with sp^2^‐hydridization of the two boron atoms.^14^ Importantly, all hitherto known dicationic diboranes are symmetrically substituted, and lack the advantageous effect of a polarized B−B bond. Unsymmetrically substituted dicationic diboranes remain unknown to date.

Herein we report the comprehensive characterization of unsymmetrically substituted dicationic diboranes and a computational evaluation of their bonding situation. They are obtained by isomerization from initially formed symmetrically substituted diboranes. The isomerization process is elucidated by spectroscopy and computation and broadens our understanding of an emerging class of synthetically useful reagents.

## Results and Discussion

**Figure 2:**
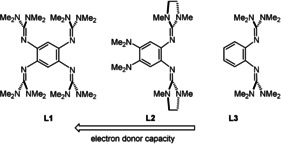
Donor substituents used in the study for the preparation of dicationic diboranes. 1,2,4,5‐tetrakis(tetramethyl‐guanidino)‐benzene (**L1**), 1,2‐bis(tetramethylguanidino)‐4,5‐bis(dimethylamino)‐benzene (**L2**), 1,2‐bis(tetramethyl‐guanidino)‐benzene (**L3**), and *E*_ox_ vs. Fc^+^/Fc in CH_2_Cl_2_: **L1** −0.62 V (*E*_1/2_=−0.70 V), **L2** −0.48 V (*E*_1/2_=−0.52 V), and **L3** 0.06 V (not reversible).

In our experiments, the diborane B_2_Cl_2_(NMe_2_)_2_ was reacted with three neutral guanidine donors **L1**–**L3** (Figure 2) in the presence of a chloride abstraction reagent (AlCl_3_, GaCl_3_ or SiMe_3_OTf). While **L3** is commercially available and **L1** known from previous reports,^15^, ^16^ the synthesis for **L2** had to be developed (see the Supporting Information for further details). The compounds were sorted with respect to their oxidation potentials (**L1** 15, 16 < **L2** (this work) < **L3** 17) measured by cyclic voltammetry in CH_2_Cl_2_ solutions (as shown in Figure 2). Inoue et al. used SbCl_5_ as chloride abstraction reagent for the synthesis of **5** starting with diborane B_2_Cl_2_(NMe_2_)_2_.^13^ However, this abstraction reagent could not be applied in our reactions, as first tests showed that it oxidizes the electron rich ligands (for example, **L2**) to the dication instead of abstracting chloride from the diborane reagent.

**Figure 3:**
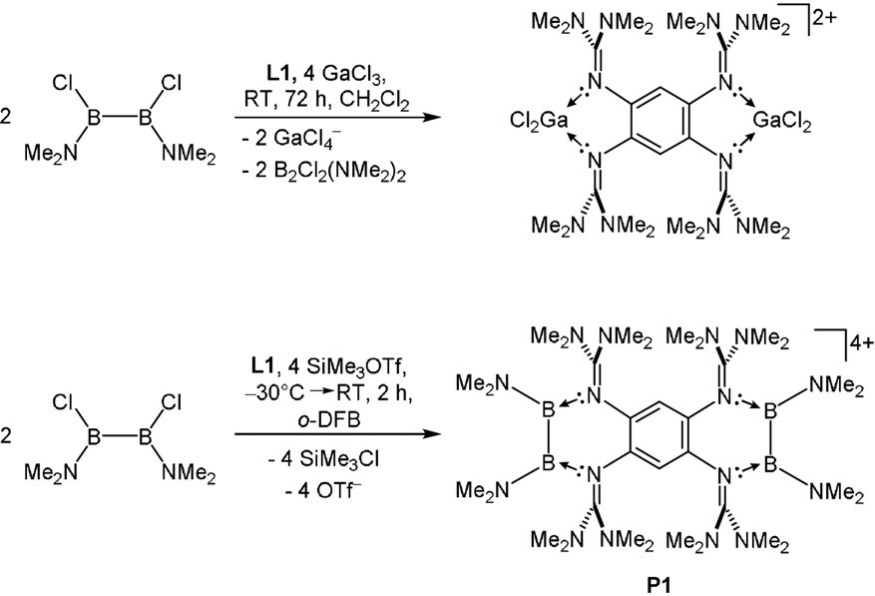
Reactions with **L1**.

We start the discussion with the results obtained with the strongest electron donor, **L1**. First, we tested reactions with the non‐oxidizing chloride abstraction reagents GaCl_3_ and AlCl_3_. However, reaction of **L1** with two equivalents of B_2_Cl_2_(NMe_2_)_2_ and four equivalents of GaCl_3_ yielded the salt [(**L1**)(GaCl_2_)_2_](GaCl_4_)_2_ (Figure 3), which crystallized from the reaction mixture and was isolated with a yield of 89 %. Hence, instead of chloride abstraction from the diborane, GaCl_3_ underwent self‐ionization to give GaCl_2_^+^ stabilized by **L1**. The use of an excess of GaCl_3_ also led not to the desired product and a similar pathway was observed with AlCl_3_.

**Figure 4:**
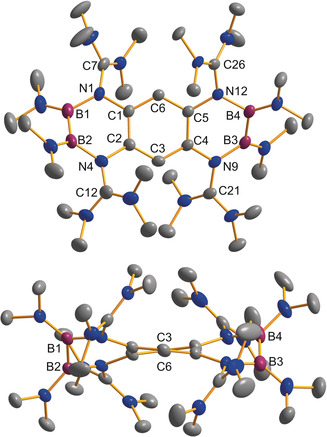
Illustration of the structure of the tetracationic bis‐diborane **P1** (ellipsoids set at 50 % probability). All hydrogen atoms and the four TfO^−^ counterions are omitted.^26^

Next, we tried Me_3_SiOTf as chloride abstraction reagent. To our delight, this reaction gave the tetracationic bis(diborane) **P1** in a yield of 80 %. The structure is in line with sp^2^‐hybridization of all boron atoms in **P1**. The two B−B bonds are 1.688(8) and 1.700(6) Å long, which are in the typical range for B−B single bonds.^18^ Noticeable is the distortion of the aromatic backbone between the C2/C3/C4 and C5/C6/C1 plane of 12.7° (Figure 4). We suggest the reason for the distortion to be the steric demand of the NMe_2_ groups in combination with the presence of one distorted six‐membered ring on both sides of the aromatic backbone. The oxidation of ligand **L1** can be excluded owing to the bond lengths in the central C_6_ ring and the absence of the characteristic deep green color of **L1** ^2+^. Noteworthily, bis‐diborane **P1** turned out as very robust. Heating a solution of **P1**(OTf)_4_ in CH_3_CN to a temperature of 80 °C for 24 h also did not lead to traceable changes.

**Figure 5:**
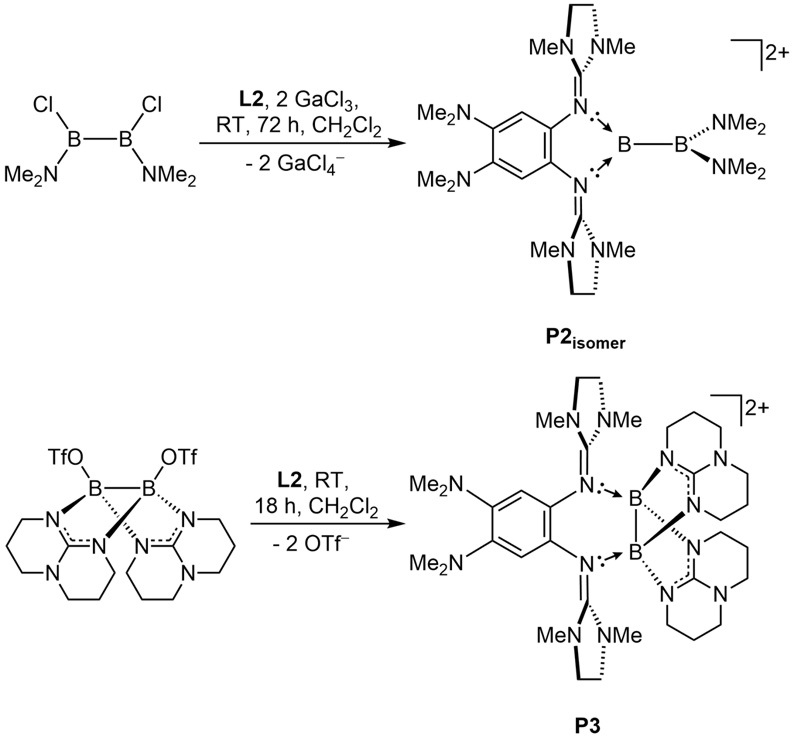
Reactions with **L2**.

**Figure 6:**
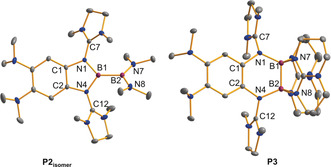
Illustration of the structures of the dicationic diboranes **P2_isomer_** and **P3** (ellipsoids set at 50 % probability). All hydrogen atoms and the GaCl_4_^−^ counterions are omitted.^26^

Subsequently, we carried out experiments with the weaker electron‐donor **L2**. Due to the lower Lewis basicity of the dimethylamino groups, only the two guanidino groups were expected to bind to the boron atoms. In this case, the use of GaCl_3_ as chloride abstraction reagent was successful and a new dicationic diborane was isolated in a yield of 84 %. To our surprise, the ^11^B NMR spectrum for the product dissolved in CD_3_CN indicated two chemically inequivalent boron atoms (*δ*=34.2 and 31.2 ppm). The compound was crystallized from CH_3_CN/Et_2_O solutions, and single‐crystal X‐ray diffraction (SCXRD) analysis verified the formation of an unsymmetrically substituted dicationic diborane **P2_isomer_** (Figure 5, Figure 6). Obviously, the substituents at boron were subjected to a 1,2‐migration process. To complete our studies on **L2**, we also synthesized the symmetric dicationic diborane **P3** with sp^3^‐hybridized boron atoms by reaction of **L2** with [B(hpp)(OTf)]_2_ (hpp=1,3,4,6,7,8‐hexahydro‐2*H*‐pyrimido[1,2‐α]pyrimidate).^19^ This reaction shows that **L2** is also capable to bind symmetrically to both boron atoms of a diboron reagent. The B−B bond distances measure 1.714(2) Å in **P2_isomer_** and 1.696(3) Å in **P3**, values that fall into the typical range of B‐B single bonds.^18^

With respect to free **L2**, the former imino bonds (N1−C7/N4−C12) are considerably elongated (from 1.279(2) in **L2** to 1.377(2)/1.382(2) Å in **P2_isomer_** and 1.370(2)/1.377(2) Å in **P3**). These changes indicate that in both cases the positive charge is delocalized into the guanidino groups.

Notably, all of the B−N bonds are significantly shorter in **P2_isomer_** with two sp^2^‐hybridized boron atoms that could establish π‐interactions compared to **P3** with two sp^3^‐hybridized boron atoms.

Finally, we attempted the synthesis of a dicationic diborane stabilized by **L3**. B_2_Cl_2_(NMe_2_)_2_ was reacted in CH_2_Cl_2_ (room temperature, 72 h) with **L3** in the presence of GaCl_3_.

**Figure 7:**
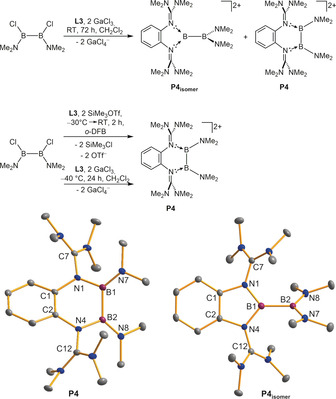
Top: Reaction leading to the isomers **P4** and **P4_isomer_**. From the experiments we propose **P4** to be the kinetic and **P4_isomer_** the thermodynamic product. Bottom: Structures of the two isomers **P4** and **P4_isomer_** in the solid state (ellipsoids set at 50 % probability). All hydrogen atoms and the GaCl_4_^−^ counterions are omitted.^26^

Interestingly, ^1^H NMR spectroscopic analysis of the reaction product indicated a mixture of the two isomeric dicationic diboranes **P4** and **P4_isomer_** (Figure 7) in a ratio of 43:57.

The isomerization from **P4** to **P4_isomer_** proceeded at room temperature in solution. Thus, under the given conditions, it was not possible to obtain **P4** in pure form. However, both isomers crystallized from a CH_3_CN/Et_2_O solution at −40 °C in the form of cube‐shaped crystals. Careful crystal picking enabled SCXRD analysis of both isomers (Figure 7). Some structural data of **P4** to **P4_isomer_** are compared in Table 1. The B−B bond is slightly longer in **P4_isomer_**, but both B−B bond lengths are in a region typical for B−B single bonds.^18^ Both compounds display long N1−C7 and N4−C12 bond distances (compare with free **L3**; these bond distances measure 1.291(3) and 1.301(3) Å^20^), indicating charge delocalization into the guanidino groups. Pure **P4** was finally obtained as TfO^−^ salt by reacting B_2_Cl_2_(NMe_2_)_2_ with **L3** at −30 °C in the presence of MeSiOTf in *o*‐difluorobenzene solution. The suspension was warmed to room temperature and stirred for additional 2 h. Then the solvent was removed to give **P4**(OTf)_2_ before significant isomerization took place. Fortunately, we were also able to obtain pure **P4**(GaCl_4_)_2_ by reacting **L3** with GaCl_3_ at −40 °C for 24 h in CH_2_Cl_2_.

**Table 1:** Selected bond lengths [Å] of the diboranes as obtained by X‐ray diffraction analyses.^26^

	**P1**(OTf)_4_	**P2_isomer_**(GaCl_4_)_2_	**P3**(OTf)_2_	**P4**(GaCl_4_)_2_	**P4**_**isomer**_(GaCl_4_)_2_
B1−B2	1.688(8)	1.714(2)	1.696(3)	1.690(2)	1.719(2)
B3−B4	1.700(6)	–	–	–	–
B1−N1	1.485(6)	1.457(2)	1.516(2)	1.493(2)	1.415(2)
B3−N9	1.497(5)	–	–	–	–
B1−N4	–	1.450(2)	–	–	1.468(2)
B1−N7	1.389(6)	–	1.562(3)	1.498(2)	–
B3−N15	1.396(5)	–	–	–	–
B2−N4	1.501(6)	1.421(2)	1.556(2)	1.395(2)	1.427(2)
B4−N12	1.487(5)	–	–	–	–
B2−N7	–	1.413(2)	–	–	1.418(2)
B2−N8	1.393(6)	–	1.562(3)	1.392(2)	–
B4−N16	1.396(6)	–	–	–	–
C1−C2	1.401(5)	1.386(2)	1.404(2)	1.419(2)	1.397(2)
C4−C5	1.409(5)	1.410(10)	1.419(2)	1.382(2)	1.392(2)
N1−C7	1.400(5)	1.377(2)	1.370(2)	1.391(2)	1.395(2)
N9−C21	1.388(5)	–	–	–	–
N4−C12	1.381(5)	1.382(2)	1.377(2)	1.387(2)	1.384(2)
N12−C26	1.396(5)	–	–	–	–

**Figure 8:**
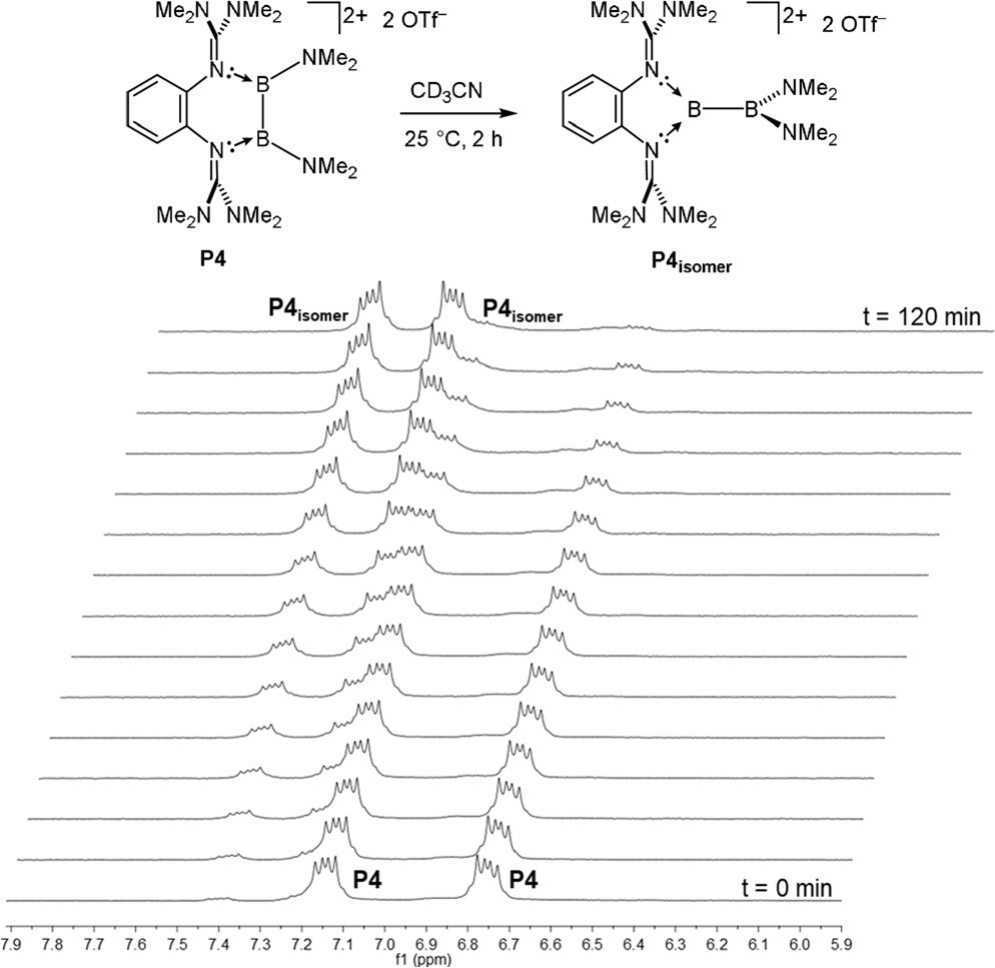
Quantitative isomerization from the symmetrically substituted diborane **P4** to the unsymmetrically substituted **P4_isomer_** in CD_3_CN at 25 °C as observable from the ^1^H NMR spectra in the region of the aromatic C−H protons (see the Supporting Information for details).

NMR studies showed that **P4** is quantitatively converted in CH_3_CN solution in 2 h at 25 °C to the thermodynamically preferred isomer **P4_isomer_**. The rate of the isomerization process **P4**→**P4_isomer_** for the dications with TfO^−^ counterions in CH_3_CN solution was studied for several temperatures (*T=*298.2, 308.4 and 318.2 K) by ^1^H NMR spectroscopy (Figure 8). An activation enthalpy Δ*H* ^≠^=49.86±2.04 kJ mol^−1^ and activation entropy of Δ*S* ^≠^=−143.5±7.1 J mol^−1^ K^−1^ were derived from an Eyring plot of the first order rate constants (see the Supporting Information for details). It should be noted that rearrangements of substituents in neutral diboranes upon addition of a donor (for example, phosphines or carbenes) were observed previously.^12^, ^21^, ^22^ For example, addition of PEt_3_ to 1,2‐dibromo‐1,2‐dimesityldiborane(4), Mes(Br)B−B(Br)Mes, resulted in the isomerization of the (not detected) initial addition product Mes(Br)B−B(Br)Mes(PEt_3_) to give (Mes)_2_B−BBr_2_(PEt_3_). Calculations predicted a barrier of Δ*G* ^≠^=96.3 kJ mol^−1^ for this base‐induced isomerization. Without base addition, the isomerization does not occur, despite the barrier height for Mes(Br)B−B(Br)Mes→(Mes)_2_B−BBr_2_ is only slightly higher (Δ*G* ^≠^=115.2 kJ mol^−1^), but the isomerization is now endergonic (Δ*G*=+15.1 kJ mol^−1^).^22^ It is therefore impossible to interconvert the two isomers thermally. By contrast, **P_4_**→**P_4isomer_** isomerization is a significantly exergonic reaction (Δ*G*_298K_=−60.3 kJ mol^−1^, see computational analysis below). The experimentally derived barrier of Δ*G* ^≠^_298K=_93.48±0.17 kJ mol^−1^ is very close to that calculated for the Mes(Br)B−B(Br)Mes(PEt_3_) isomerization.

The addition of catalytic amounts of KF in the presence of [18]‐crown‐6 accelerates the **P_4_**→**P_4isomer_** isomerization drastically. In our experiments (see the Supporting Information for details), the process was completed in less than 15 min at 22 °C, whereas it took 2 h (*t*_1/2_=44 min, 25 °C) in the absence of KF (for the dications with TfO^−^ counterions). Hence the isomerization is catalyzed by nucleophiles. To obtain more information about the effect of nucleophiles, we repeated the isomerization experiments with **P4**(GaCl_4_)_2_ in CD_3_CN at different temperatures (*T=*293.2, 313.6, and 324.4 K). To our surprise, the best fit of the experimental data was obtained now by assuming a zero‐order rate law (see the Supporting Information), in contrast to the first‐order rate constants derived for the isomerization of **P4**(TfO)_2_. However, the rate decrease upon increase of the concentration argues for a more complex mechanism (see the Supporting Information). An activation enthalpy of Δ*H* ^≠^=67.09±4.36 kJ mol^−1^ and an activation entropy of Δ*S* ^≠^=−101.3±10.9 J mol^−1^ K^−1^ were derived from an Eyring plot of the zero order rate constants (see the Supporting Information for details). Hence the activation enthalpy was higher, but the activation entropy lower than for isomerization of **P4**(TfO)_2_, resulting in a relatively small change of the activation free energy from Δ*G* ^≠^_298K_=93.48±0.17 kJ mol^−1^ for **P4**(TfO)_2_ to 97.86±0.20 kJ mol^−1^ for **P4**(GaCl_4_)_2_. The change from first‐order reaction for **P4**(TfO)_2_ to zero‐order for **P4**(GaCl_4_)_2_ as well as the significant negative entropy of activation in both cases cannot be understood by a thermal (nucleophile‐free) isomerization process. Furthermore, the calculated transition state energy for the purely thermal intramolecular isomerization amounts to a much higher value of Δ*G* ^≠^_298K=_181 kJ mol^−1^ (Supporting Information, Figure S24). One possible explanation might be the catalysis by Cl^−^ ions generated in small quantities in a rate‐determining pre‐equilibrium reaction from GaCl_4_^−^. A related phenomenon was reported for the iodination of acetone, where the enol form as reactive species is generated in small amounts in the rate‐controlling step from the unreactive keto form.^23^ Herein, the reaction follows a pseudo zero‐order kinetic on the iodine. Since the isomerization process even took place when a suspension of **P4**(GaCl_4_)_2_ in CH_2_Cl_2_ was stirred for 4 d (see the Supporting Information), we could exclude a significant role of the solvent CD_3_CN as nucleophile.

**Figure 9:**
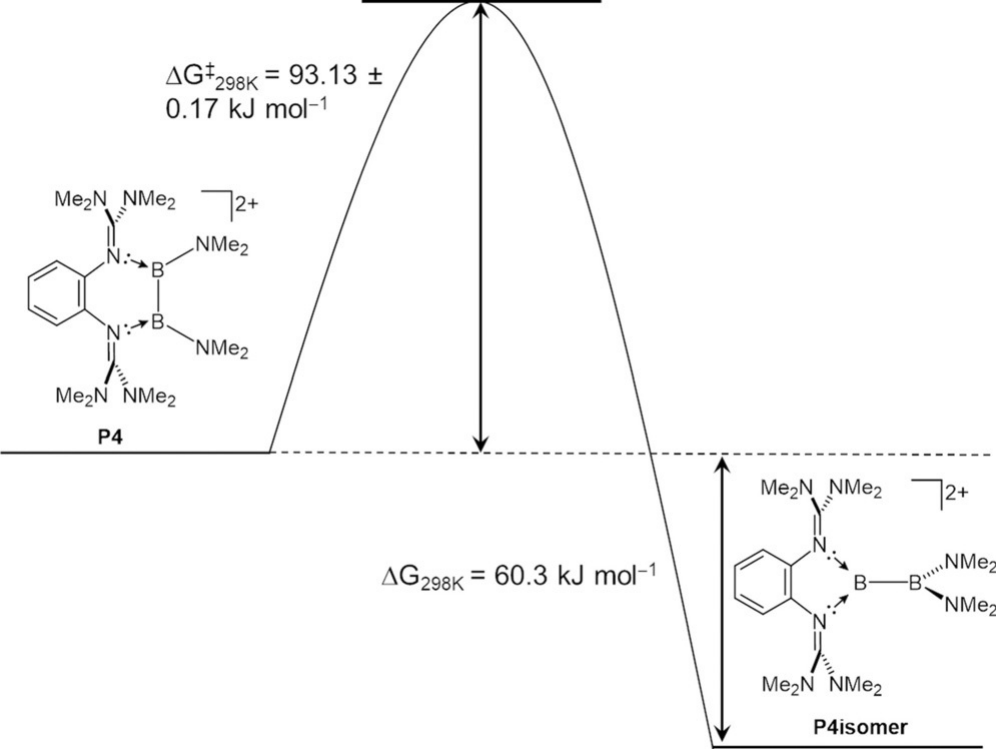
Gibbs free energy change (from calculations, B3LYP+D3/def2‐TZVP; COSMO *ϵ*_r_=37.5) and activation Gibbs free energy (from NMR experiments at variable temperature for the dications with TfO^−^ counterions in CH_3_CN solution) for the TfO^−^ catalyzed **P_4_**→**P_4isomer_** isomerization.

Next, the experimental data was backed‐up by further quantum chemical calculations. Structure optimizations at the B3LYP+D3/def2‐TZVP level of theory reproduced the structure of both isomers very well (see the Supporting Information). The unsymmetrical isomer was preferred over the symmetric one (Δ*G*_298K_(**P1**_**isomer**_−**P1**)=−105 kJ mol^−1^ (rearrangement of both diborane units), Δ*G*_298K_(**P2_isomer_**−**P2**)=−43 kJ mol^−1^, Δ*G*_298K_(**P4_isomer_**−**P4**)=−49 kJ mol^−1^; Figure 9). Calculations with inclusion of solvation (COSMO with *ϵ*_r_=37.5) found a difference in the Gibbs free energy of Δ*G*_298K_=−60 kJ mol^−1^ in favor of **P4_isomer_**. Hence the symmetric isomers are the kinetic and the unsymmetrical isomers the thermodynamic reaction products. By contrast, in the case of compound **5** synthesized by Inoue et al. (see Figure 1), the symmetric isomer is preferred by −11 kJ mol^−1^ (see the Supporting Information).

The barrier for isomerization appears to depend on the electron‐donor capacity of the guanidine substituent. The computed transition state for the isomerization of **P4**→**P4_isomer_** revealed, that the migration of a guanidino group causes the barrier, but not the subsequent migration of the −NMe_2_ unit. This situation should persist likewise for the case of a nucleophile catalyzed isomerization. With the relatively weak electron donor **L_3_**, the barrier is below 100 kJ mol^−1^. With **L2**, the barrier should be smaller, since the unsymmetrical product **P2_isomer_** is formed immediately, and we were not able to detect its symmetric isomer **P2**. Although **L1** is the strongest electron donor (the compound with the lowest oxidation potential), only the symmetric isomer **P1** is formed, and not the unsymmetrical **P1**_**isomer**_. At first glance this observation contradicts the prediction that the barrier lowers with increasing electron donor character of the guanidine substituents. However, the presence of the second dicationic diborane unit strongly reduces the electron–donor character of **L1** (the electron donor capacity of [(B_2_(NMe_2_)_2_)**L1**]^2+^ is certainly lower than that of **L2** or **L3**).

The experimental ^11^B NMR chemical spectra of **P4_isomer_** (*δ*=37.7 and 30.7 ppm) featured the presence of two substantially different boron atoms, and thus of a polarized B−B bond. To understand the electronic structure and bonding situation of **P4_isomer_** in more depth, further quantum chemical calculations and bond analysis tools were carried out. The bond polarization in **P4_isomer_** was inspected first by NBO charge analysis. Indeed, the boron atom coordinated to the guanidines is substantially more positive (+0.84) than the boron atom in the B(NMe_2_)_2_ unit (+0.58). In agreement to that, the fluoride ion affinity of the guanidino substituted boron center is by 130 kJ mol^−1^ higher as that of the B(NMe_2_)_2_ unit. This unbalanced charge distribution can be rationalized by the stronger π‐donor properties of the NMe_2_ units in comparison to the positively charged guanidino functionalities (see below). The influence of the additional amino groups in **P2_isomer_** can also be unraveled by NBO charge analysis. The boron atom coordinated to the guanidines becomes less positive (+0.72) than in **P4_isomer_** (+0.84) owing to the better π‐donor capability of the ligand. The boron in the B(NMe_2_)_2_ unit remains almost unchanged (+0.60). Indeed, the weaker polarization of the B−B bond within **P2_isomer_** is in line with the decreasing ^11^B NMR shift separation (Δ(*δ* ^11^B)=3.0 ppm) in comparison to **P4_isomer_** (Δ(*δ* ^11^B)=7.0 ppm) and exemplifies the unique handle to control bond ionicity by alteration of substituents at the bisguanidinium donor entity.

**Figure 10:**
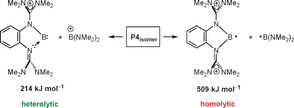
Comparison betweeen heterolytic and homolytic B−B bond cleavage of **P4_isomer_**. The difference in the fragmentation energy of the relaxed fragments is Δ*E*=−295 kJ mol^−1^ for the heterolytic bond cleavage.

Next, the homo‐ and heterolytic bond dissociation (Figure 10) energies of the B−B bonds in **P4_isomer_** were computed (PW6B95+D3(BJ)/def2‐QZVPP) and compared with that of the symmetric, neutral analogue tetrakis(dimethylamino)diborane (TDADB). Interestingly, the heterolytic bond dissociation enthalpy for **P4_isomer_** is 295 kJ mol^−1^ more favorable than the homolytic bond rupture.

In contrast, for the unpolarized TDADB, the homolytic bond cleavage is clearly the favored process. According to the IUPAC definition, this would imply a type of dative bond interaction between the two boron atoms in **P4_isomer_**.

To unravel the question of dative vs. covalent B−B bonding further, the electron density was inspected by QTAIM. Bond descriptors for the B−B bonds revealed similar and predominantly covalent bond characteristics for both compounds, with the B−B bond in **P4_isomer_** being more polarized/ionic in comparison to the symmetrical TDADB (Supporting Information, Table S5). Finally, energy decomposition analysis between the fragments of homolytic and heterolytic B−B bond cleavage was performed. Several EDA studies suggested that the fragmentation that corresponds to the best description of the bonding situation (dative/heterolytic or covalent/homolytic) is the one with the smallest orbital interaction energy term Δ*E*_orb_, as it requires the smallest change in electronic charge distribution to conform to the electronic structure of the molecule.^24^ The numerical results of the EDA (Supporting Information, Table S6) revealed that for both **P4_isomer_** and TDADB, the electron‐sharing bond is the more realistic representation. In turn, from the coulombic attraction energy it can be concluded, that the coulombic repulsion between the two cationic fragments after heterolytic bond cleavage rationalizes the strongly favored heterolytic dissociation in **P4_isomer_**. Moreover, EDA reveals a significant amount of dispersion interaction that further stabilizes the B−B bond in **P4_isomer_** Importantly, none of the given results support a dative B−B bonding within **P4_isomer_** that is pretended by the trend in homolytic vs. heterolytic bond dissociation enthalpies.

**Figure 11:**
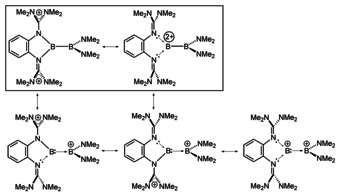
Possible resonance structures for **P4_isomer_**. According to our quantum‐chemical bond analysis, the structures in the box should be of higher relevance.

To conclude, from the various possible Lewis representations assembled in Figure 11, the most realistic ones are those in the box. Owing to the larger electron‐donor capability of **L2**, the structure in which all positive charge is accumulated on the bisguanidine unit is more important for **P2_isomer_** than for **P4_isomer_**.

Hence the degree of B−B bond polarization is efficiently tunable by the electron‐donor character of the bisguanidine substituent at hand in our group.^25^

**Figure 12:**
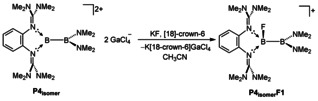
Reaction of **P4_isomer_** with KF in the presence of 18‐crown‐6 as suggested by NMR spectroscopy, HR ESI mass spetrometry, and quantum chemical calculations (see the Supporting Information).

It will be interesting to see whether this picture is also reflected in the reactivity of those compounds. In a first reactivity test, we were able to convert **P4_isomer_** with KF in the presence of [18]‐crown‐6 to the suggested (for a discussion, see the Supporting Information) monocationic diborane **P4_isomer_F1** with a mixed sp^3^–sp^2^ hybridization (Figure 12). The ^11^B NMR spectra displays two remarkable different signals at 36.06 and 7.42 ppm.

## Conclusion

We have reported the first unsymmetrically substituted dicationic diboranes. Reactions between electron‐rich bisguanidines and B_2_Cl_2_(NMe_2_)_2_ in the presence of chloride abstraction reagents first led to symmetrically substituted dicationic diboranes, which undergo nucleophilic catalyzed isomerization to the energetically preferred unsymmetrical diborane isomers. A comparison between unsymmetrical diboranes with different bisguanidine substitutents indicates that the B−B bond polarization could be tuned by the electron‐donor character of the bisguanidine. Experiments and theoretical analysis of the fluoride ion affinity discloses significant differences between the two boron atoms. To unleash the full potential of this new dicationic unsymmetrically substituted diboranes, a detailed analysis of the reactivity is under current investigations.

## Conflict of interest

The authors declare no conflict of interest.

## Supporting information

As a service to our authors and readers, this journal provides supporting information supplied by the authors. Such materials are peer reviewed and may be re‐organized for online delivery, but are not copy‐edited or typeset. Technical support issues arising from supporting information (other than missing files) should be addressed to the authors.

Supplementary
