# Supplementary material for: Desymmetrization of Dicationic Diboranes by Isomerization Catalyzed by a Nucleophile
Source: Angew Chem Int Ed Engl. 2020 Apr 6;59(23):9127–33. doi: 10.1002/anie.202001640 (PMC7317786; doi:10.1002/anie.202001640)
Supplement: Supplementary file 1 — Supplementary [file ANIE-59-9127-s001.pdf]

Angewandte Chemie
Eine Zeitschrift der Gesellschaft Deutscher Chemiker

Supporting Information

Desymmetrization of Dicationic Diboranes by Isomerization Catalyzed by a Nucleophile

*Florian Schön, Lutz Greb, Elisabeth Kaifer, and Hans-Jörg Himmel**

anie_202001640_sm_miscellaneous_information.pdf

SUPPORTING INFORMATION

Abstract: Cationic monoboranes exhibit a rich chemistry. By constrast, only a few cationic diboranes are known, that all are symmetrically substituted. In this work, we report the first unsymmetrically substituted dicationic diboranes, featuring sp^2-sp^2-hybridized boron atoms. The compounds are formed by intramolecular rearrangement from preceeding isomeric symmetrically substituted dicationic diboranes, a process that is catalysed by nucleophiles. From the temperature-dependence of the isomerization rate, activation parameters for this unprecedented rearrangement are derived. The difference in fluoride ion affinity between the two boron atoms and the bonding situation in these unique unsymmetric dicationic diboranes are evaluated.

DOI: 10.1002/anie.2016XXXXX

Content

1. General Information ... 3
2. Experimental Procedures ... 4
N1,*N1*,*N2*,*N2*-Tetramethylbenzene,1,2,4,5-tetramine dihydrochloride ... 4
L2 ... 4
[**L1**($GaCl_2$)$_2$]($GaCl_4$)$_2$ ... 4
P1(OTf)$_4$ ... 4
P3(OTf)$_2$ ... 5
P2$_{isomer}$($GaCl_4$)$_2$ ... 5
P2$_{isomer}$($AlCl_4$)$_2$ ... 5
P4($GaCl_4$)$_2$ and **P4**$_{isomer}$($GaCl_4$)$_2$ ... 6
P4(OTf)$_2$ and **P4**$_{isomer}$(OTf)$_2$ ... 6
3. Analytical data ... 8
X-ray crystallographic details ... 10
Crystal data for [**L1**($GaCl_2$)$_2$]($GaCl_4$)$_2$: ... 11
Crystal data for **P1**(OTf)$_4$: ... 12
Crystal data for **L2**: ... 13
Crystal data for **P2**$_{isomer}$($GaCl_4$)$_2$: ... 14
Crystal data for **P2**$_{isomer}$($AlCl_4$)$_2$: ... 15
Crystal data for **P3**[OTf]$_2$: ... 16
Crystal data for **P4**($GaCl_4$)$_2$: ... 17
Crystal data for **P4**$_{isomer}$($GaCl_4$)$_2$: ... 18
Crystal data for **P4**$_{isomer}$(OTf)$_2$: ... 19
4. DFT calculations: ... 20
DFT calculations on the bonding mode of P4$_{isomer}$: ... 32
5. Computation of the transition state of P4 isomerisation ... 37
6. Kinetic data for the isomerisation process of P4 ... 39
Reaction zero-order kinetic on [P4]($GaCl_4$)$_2$ ... 46
7. NMR Spectra: ... 47
8. References ... 65

1. General Information

If not stated otherwise, all synthetic procedures were carried out under an Ar inert-gas atmosphere using standard Schlenk techniques or in a glove box (MBraun, LABstar) under N_2 atmosphere. Solvents were dried with an MBraun Solvent Purification System, degassed by three freeze-pump-thaw cycles and stored over molecular sieves prior to their use. 1,2-Difluorobenzene was purchased from abcr GmbH, dried over molecular sieves (4 Å) and degassed by bubbling Ar into the solution for 30 min. The synthesis of 4,5-Bis(dimethylamino)-1,2-dinitrobenzene[S1], $[(TfO)B(hpp)]_2$[S2], **L1**[S3] and **L3**[S4] generally followed literature procedures. Elemental analysis was performed at the Microanalytical Laboratory of the University of Heidelberg using the vario EL and vario MICRO cube devices from Elementar Analysensysteme GmbH. NMR spectra were recorded on a Bruker Avance II 400 spectrometer (BBFO probe) or Bruker Avance III 600 spectrometer. Solvent resonances were taken as references for all ^1H NMR or ^{13}C NMR spectra and ^{11}B chemical shifts are given relative to $BF_3 \cdot Et_2O$. If not stated otherwise, NMR spectra were recorded at 298 K. UV/Vis spectra were recorded with a Cary 5000 spectrophotometer. CV measurements were carried out at a Metrohm Autolab PGSTAT 204 potentiostate/galvanostate with an Ag/AgCl reference electrode, Pt rod counter electrode and glassy carbon working electrode. The curves were recorded at room temperature with different scan rates (50, 100, 700 $m \cdot V \cdot s^{-1}$). CH_2Cl_2 or CH_3CN was used as solvent for the individual compounds ($c = 10^{-3}$ M), whereas $nBu_4N(PF_6)$ or $nBu_4(ClO_4)$ was employed as supporting electrolyte ($c = 0.1$ M). Infrared spectra were recorded as solids on an ATR crystal with an AGILENT *Cary* 630 FTIR spectrometer.

2. Experimental Procedures

N1,*N1*,*N2*,*N2*-Tetramethylbenzene,1,2,4,5-tetramine dihydrochloride: A slight modified version of the previously published synthesis of N^1,N^1,N^2,N^2-Tetramethylbenzene,1,2,4,5-tetramine[S1] is used. Herein, a suspension of 4,5-Bis(dimethylamino)-1,2-dinitrobenzene (800 mg, 3.15 mmol) and 10% Pd/C (240 mg) in methanol (75 ml) is stirred at RT under a dihydrogen atmosphere (50 bar) in an autoclave for 48 h. After the filtration of the suspension under an inert atmosphere over a pad of Celite, that is rinsed with methanol (2 x 10 ml), HCl in Et_2O (8 ml, 1 M) is added to the filtrate to stabilize the highly air sensitive amine. After removal of the solvent in vacuo, a slightly pink residue is gained which is used immediately for the synthesis of **L2** without further purification.

L2: Oxalyl chloride (4.10 ml, 47.0 mmol) is slowly added to a solution of 1,3-Dimethylimidazolin-2-on (1.01 ml, 9.40 mmol) in anhydrous $CHCl_3$ (7 ml) and the reaction mixture is refluxed for 16 h. After removal of the solvent under reduced pressure, the pale-yellow solid is washed with Et_2O (3 x 10 ml), yielding 2-Chlor-1,3-dimethyl-4,5-dihydro-1H-imidazolium chloride as a colorless product. An ice cooled solution of 2-Chlor-1,3-dimethyl-4,5-dihydro-1H-imidazolium chloride in CH_3CN (9.5 ml) is added slowly to an cooled solution of N^1,N^1,N^2,N^2-Tetramethylbenzene,1,2,4,5-tetramine dihydrochloride (from previous step). Triethylamine (4.4 ml, 94.0 mmol) is added and the reaction mixture is stirred at 0 °C for 1 h. Afterwards, the suspension is allowed to warm to RT and stirred for additional 24 h. A solution of NaOMe in MeOH (4.0 ml, 5.4 M) is added to the filtrate und the solvent is removed in vacuo. The brown residue is washed with water (2 x 10 ml) and recrystallized from CH_3CN, forming colourless crystals which are collected and dried under reduced pressure to afford the product as beige solid (770 mg, 1.99 mmol, 63% over two steps).
^{1}H NMR (600 MHz, CD_3CN): δ = 6.23 (s, 2 H), 3.14 (s, 8 H), 2.65 (s, 12 H), 2.55 (s, 12 H) ppm. ^{13}C NMR (150 MHz, CD_3CN): δ = 153.85, 140.18, 136.91, 113.62, 49.20, 43.11, 34.93 ppm. Elemental analysis (%) for $C_{20}H_{34}N_8$ (386.55): calcd. C 62.14, H 8.87, N 28.99; found C 62.22, H 8.56, N 29.16. UV/Vis (CH_3CN, $c = 5.00 \cdot 10^{-5}$): λ_{max} (ε in L mol^{-1} cm^{-1}) = 250 ($2.28\cdot10^4$), 290 ($8.43\cdot10^3$), 330 ($7.44\cdot10^3$) nm. IR (ATR): ν = 2975 (w), 2950 (w), 2913 (w), 2894 (w), 2832 (w), 2813 (w), 2770 (w), 1663 (vs), 1655 (vs), 1590 (s), 1495 (s), 1478 (s), 1432 (s), 1407 (s), 1382 (s), 1333 (m), 1293 (m), 1276 (s), 1264 (s), 1242 (s), 1212 (m), 1192 (s), 1177 (m), 1142 (s), 1123 (m), 1096 (m), 1094 (m), 1067 (m), 1035 (s), 1020 (s), 990 (s), 949 (s), 917 (s), 868 (s), 852 (m), 770 (m), 733 (m), 716 (m), 702 (m), 690 (m), 677 (m), 641 (m), 616 (m), 582 (w), 503 (w), 493 (m) cm^{-1}.

[**L1**($GaCl_2$)$_2$]($GaCl_4$)$_2$: A solution of $GaCl_3$ (53.1 mg, 304 µmol) in CH_2Cl_2 (1 ml) is added to a solution of $B_2Cl_2(NMe_2)$ (27.3 mg, 25.0 µl, 152 µmol) and **L1** (30.0 mg, 75.5 µmol) in CH_2Cl_2 (1 ml). The reaction mixture is stirred for 72 h at RT. A colourless precipitate is formed, which is isolated by filtration, washed with CH_2Cl_2 (3 x 1 ml) and dried in vacuo. The residue is redissolved in CH_3CN (0.6 ml), overlayered with Et_2O and stored at –40 °C to afford colourless crystals. The supernatant is removed, and the crystals are dried at reduces pressure to afford a colourless powder (83.0 mg, 67.2 mmol, 89%).
^{1}H NMR (400 MHz, CD_3CN): δ = 6.14 (s, 2 H), 3.05 (s, 24 H), 2.91 (s, 24 H) ppm. ^{13}C NMR (100 MHz, CD_3CN): δ = 163.27, 132.37, 108.88, 44.63, 41.52 ppm. ^{71}Ga NMR (122 MHz, CD_3CN): δ = 251.40 (s) ppm. Elemental analysis (%) for $C_{26}H_{50}N_{12}Ga_4Cl_{12} \cdot CH_3CN$ (1276.12): calcd. C 26.35, H 4.19, N 14.27; found C 26.32, H 4.23, N 14.49. UV/Vis (CH_3CN, $c = 5.00 \cdot 10^{-5}$): λ_{max} (ε in L mol^{-1} cm^{-1}) = 240 ($3.69\cdot10^4$), 280 (sh, $2.18\cdot10^4$), 320 ($2.36\cdot10^4$), 360 (sh, $1.51\cdot10^4$) nm. IR (ATR): ν = 3020 (vw), 2963 (w), 2940 (w), 2905 (w), 2873 (w), 2800 (vw), 1635 (m), 1592 (s), 1588 (m), 1522 (s), 1483 (s), 1468 (s), 1448 (m), 1403 (vs), 1319 (s), 1279 (s), 1232 (s), 1178 (s), 1168 (s), 1141 (s), 1106 (m), 1062 (m), 1040 (m), 974 (m), 921 (m), 886 (s), 870 (s), 844 (m), 831 (m), 813 (s), 726 (s), 715 (m), 681 (w) cm^{-1}.

P1(OTf)$_4$: A solution of Me_3SiOTf (67.5 mg, 55.0 µl, 304 µmol) in *o*-difluorobenzene (1 ml) is cooled to –30 °C and added to a precooled solution of $B_2Cl_2(NMe_2)$ (21.8 mg, 20.0 µl, 121 µmol) and **L1** (32.0 mg, 60.4 µmol) at –30 °C in *o*-difluorobenzene (1 ml). After the addition, the reaction mixture is allowed to warm to room temperature and stirred for 2 h. The solvent of the suspension is removed at reduced

pressure and the residue is washed with CH_2Cl_2 (2 x 1 ml). The colourless solid is dried at reduced pressure, redissolved in CH_3CN (0.6 ml), overlayered with Et_2O and stored at –40 °C to afford colourless crystals. The supernatant is removed, and the crystals are dried at reduces pressure to afford a colourless powder (65.0 mg, 48.3 mmol, 80%).
^{1}H NMR (400 MHz, CD_3CN): δ = 6.63 (s, 2 H), 3.38 (s, 24 H), 3.01 (s, 24 H), 2.80 – 2.60 (br. s, 24 H) ppm. ^{13}C NMR (100 MHz, CD_3CN): δ = 158.14, 131.32, 101.10, 42.81, 42.78 ppm. ^{11}B NMR (128 MHz, CD_3CN): δ = 30.52 ppm. $^{11}B\{^{1}H\}$ NMR (128 MHz, CD_3CN): δ = 30.73 ppm. ^{19}F NMR (376 MHz, CD_3CN): δ = 79.25 ppm. Elemental analysis (%) for $C_{38}H_{74}B_4F_{12}N_{16}O_{12}S_4 \cdot CH_3CN$ (1346.47): calcd. C 34.62, H 5.59, N 17.16; found C 34.85, H 5.49, N 17.19.

P3(OTf)$_2$: A solution of **L2** (40.2 mg, 104 µmol) and [(TfO)B(hpp)]$_2$ (62.0 mg, 104 µmol) in CH_2Cl_2 (1 ml) is stirred for 18 h at RT. The yellow solution is filtered via a syring filter, overlayered with Et_2O and stored at –40 °C to afford colourless needles. The supernatant is removed, and the crystals are dried at reduced pressure to afford a colourless powder (91.0 mg, 93.6 µmol, 90%).
^{1}H NMR (400 MHz, CD_3CN): δ = 5.76 (s, 2 H), 3.92 – 3.84 (m, 8 H), 3.21 – 3.11 (m, 16 H), 2.91 (s, 12 H), 2.66 (s, 12 H), 1.84 – 1.82 (m, 8 H) ppm. ^{13}C NMR (100 MHz, CD_3CN): δ = 167.76, 159.97, 141.02, 129.43, 109.39, 48.21, 47.97, 42.59, 42.14, 35.01, 22.96 ppm. ^{11}B NMR (128 MHz, CD_3CN): δ = 1.62 (2 B) ppm. ^{19}F NMR (376 MHz, CD_3CN): δ = 79.32 ppm. Elemental analysis (%) for $C_{36}H_{58}N_{14}B_2F_6O_6S_2$ (982.42): calcd. C 44.00, H 5.95, N 19.96; found C 44.11, H 6.03, N 20.24. UV/Vis (CH_3CN, c = 5.00 · 10^{-5}): λ_{max} (ε in L mol^{-1} cm^{-1}) = 236 (2.69·10^{4}), 280 (sh, 7.04·10^{3}) 332 (8.82·10^{3}) nm. IR (ATR): ν = 2938 (m), 2861 (m), 2780 (w), 1580 (s), 1452 (vs), 1520 (s), 1481 (m), 1448 (m), 1410 (m), 1395 (m), 1364 (m), 1321 (m), 1260 (vs), 1220 (s), 1179 (m), 1141 (s), 1098 (s), 1047 (m), 1029 (vs), 1004 (s), 957 (w), 914 (m), 900 (m), 815 (w), 745 (m), 730 (m), 690 (w) cm^{-1}.

P2$_{\mathbf{isomer}}$($GaCl_4$)$_2$: $B_2Cl_2(NMe_2)$ (14.1 mg, 13.0 µl, 78.0 µmol) is added to a solution of **L2** (30.0 mg, 77.6 µmol) and $GaCl_3$ (27.3 mg, 155 µmol) in CH_2Cl_2 (1 ml). The reaction mixture is stirred for 72 h at RT. A colourless precipitate is formed, which is isolated by filtration, washed with CH_2Cl_2 (3 x 1.5 ml) and dried in vacuo. The residue is redissolved in CH_3CN (0.3 ml), overlayered with Et_2O and stored at –40 °C to afford colourless crystals. The supernatant is removed, and the crystals are dried at reduces pressure to afford a colourless powder (60.0 mg, 65.3 mmol, 84%).
^{1}H NMR (400 MHz, CD_3CN): δ = 6.78 (s, 2 H), 4.14 – 4.01 (m, 8 H), 2.95 (s, 12 H), 2.80 (s, 12 H), 2.68 (s, 12 H) ppm. ^{13}C NMR (100 MHz, CD_3CN): δ = 158.57, 143.74, 127.72, 104.70, 49.90, 42.06, 35.66 ppm. ^{11}B NMR (128 MHz, CD_3CN): δ = 34.20 (sh, 1 B), 30.85 (1 B) ppm. $^{11}B\{^{1}H\}$ NMR (128 MHz, CD_3CN): δ = 34.08 (sh, 1 B), 31.03 (1 B) ppm. Elemental analysis (%) for $C_{24}H_{46}N_{10}B_2Ga_2Cl_8$ (919.37): calcd. C 31.35, H 5.04, N 15.24; found C 31.44, H 5.47, N 15.49. UV/Vis (CH_3CN, c = 5.00 · 10^{-5}): λ_{max} (ε in L mol^{-1} cm^{-1}) = 248 (2.68·10^{4}), 290 (sh, 9.97·10^{3}), 320 (1.16·10^{4}) nm. IR (ATR): ν = 3005 (w), 2980 (w), 2939 (w), 2877 (w), 2852(w), 2588 (w), 2758 (w), 1624 (s), 1574 (s), 1511 (m), 1497 (s), 1451 (m), 1422 (m), 1413 (m), 1382 (s), 1302 (s), 1219 (m), 1196 (m), 1139 (s), 1126 (s), 1091 (m), 1062 (m), 1009 (m), 960 (m), 919 (m), 893 (m), 859 (m), 840 (w), 770 (w), 746 (w), 706 (w) cm^{-1}.

P2$_{\mathbf{isomer}}$($AlCl_4$)$_2$: $B_2Cl_2(NMe_2)$ (28.2 mg, 26.0 µl, 156 µmol) is added to a solution of **L2** (60.0 mg, 155.2 µmol) and $AlCl_3$ (41.4 mg, 310 µmol) in CH_2Cl_2 (2 ml). The reaction mixture is stirred for 72 h at RT. A colourless precipitate is formed, which is isolated by filtration, washed with CH_2Cl_2 (3 x 1.5 ml) and dried in vacuo. The residue is redissolved in CH_3CN (0.5 ml), overlayered with Et_2O and stored at –40 °C to afford colourless crystals. The supernatant is removed, and the crystals are dried at reduced pressure to afford a colourless powder (75.0 mg, 89.9 mmol, 58%).
^{1}H NMR (400 MHz, CD_3CN): δ = 6.78 (s, 2 H), 4.14 – 4.01 (m, 8 H), 2.95 (s, 12 H), 2.80 (s, 12 H), 2.68 (s, 12 H) ppm. ^{13}C NMR (100 MHz, CD_3CN): δ = 158.56, 143.74, 127.71, 104.70, 49.90, 42.05, 35.66 ppm. ^{11}B NMR (128 MHz, CD_3CN): δ = 34.04 (sh, 1 B), 30.62 (1 B) ppm. $^{11}B\{^{1}H\}$ NMR (128 MHz, CD_3CN): δ = 34.23 (sh, 1 B), 31.16 (1 B) ppm. Elemental analysis (%) for $C_{24}H_{46}N_{10}B_2Ga_2Cl_8$ (919.37): calcd. C 31.35, H 5.04, N 15.24; found C 31.44, H 5.47, N 15.49. UV/Vis (CH_3CN, c = 5.00 · 10^{-5}): λ_{max}

(ε in L mol^{-1} cm^{-1}) = 236 ($2.53\cdot10^4$), 290 ($9.97\cdot10^3$), 323 ($6.36\cdot10^3$) nm. IR (ATR): v = 3005 (w), 2980 (w), 2939 (w), 2877 (w), 2852(w), 2588 (w), 2758 (w), 1739 (m), 1624 (s), 1574 (s), 1511 (m), 1497 (s), 1451 (m), 1422 (m), 1413 (m), 1382 (s), 1302 (s), 1219 (m), 1196 (m), 1139 (s), 1126 (s), 1091 (m), 1062 (m), 1009 (m), 960 (m), 919 (m), 893 (m), 859 (m), 840 (w), 770 (w), 746 (w), 706 (w) cm^{-1}.

P4$(GaCl_4)_2$ and $\mathbf{P4_{isomer}}(GaCl_4)_2$: $B_2Cl_2(NMe_2)$ (14.1 mg, 13.0 µl, 78.0 µmol) is added to a solution of **L3** (23.6 mg, 77.6 µmol) and $GaCl_3$ (27.3 mg, 155 µmol) in CH_2Cl_2 (1 ml). The reaction mixture is stirred for 72 h at RT. A colourless precipitate is formed, which is isolated by filtration, washed with Et_2O (3 x 1.5 ml) and dried in vacuo. The residue is redissolved in CH_3CN (0.4 ml), overlayered with Et_2O and stored at –40 °C to afford colourless crystals. The supernatant is removed, and the crystals are dried at reduced pressure to afford a mixture of the two isomers **P4**$(GaCl_4)_2$ and $\mathbf{P4}_{isomer}(GaCl_4)_2$ (50.0 mg, 59.7 mmol, 77%).
Elemental analysis (%) for $C_{20}H_{40}N_8B_2Ga_2Cl_8$ (837.26): calcd. C 28.69, H 4.82, N 13.38; found C 29.17, H 4.36, N 12.97.

Pure **P4**$(GaCl_4)_2$ (40.0 mg, 47.8 mmol, 62%) is obtained by performing the same procedure at –40 °C with a reaction time of 24 h.
^{1}H NMR (400 MHz, CD_3CN): δ = 7.16 – 7.13 (m, 2 H), 6.75 – 6.73 (m, 2 H), 3.07 (s, 24 H), 2.85 – 2.75 (br. s, 12 H) ppm. ^{13}C NMR (100 MHz, CD_3CN): δ = 163.03, 131.30, 125.11, 119.54, 42.01 ppm. ^{11}B NMR (128 MHz, CD_3CN): δ = 33.05 ppm.

Pure $\mathbf{P4}_{isomer}(GaCl_4)_2$ is obtained by the quantitative isomerisation process at RT, redissolving **P4**$(GaCl_4)_2$ in CH_3CN and stirring for 24 h.
^{1}H NMR (400 MHz, CD_3CN): δ = 7.41 – 7.39 (m, 2 H), 7.21 – 7.18 (m, 2 H), 3.32 (s, 12 H), 2.96 (s, 12 H), 2.75 – 2.50 (br. s, 12 H) ppm. ^{13}C NMR (100 MHz, CD_3CN): δ = 158.79, 133.65, 125.78, 115.57, 43.00, 42.62 ppm. ^{11}B NMR (128 MHz, CD_3CN): δ = 37.69 (1 B), 30.69 (1 B) ppm. ^{11}B{^{1}H} NMR (128 MHz, CD_3CN): δ = 38.34 (1 B), 31.01 (1 B) ppm.

P4$(OTf)_2$ and $\mathbf{P4_{isomer}}(OTf)_2$: A solution of Me_3SiOTf (73.0 mg, 59.0 µl, 328 µmol) in *o*-difluorobenzene (1 ml) is cooled to –30 °C and added to a precooled solution of $B_2Cl_2(NMe_2)$ (29.7 mg, 27.0 µl, 165 µmol) and **L3** (50.0 mg, 164 µmol) at –30 °C in *o*-difluorobenzene (1 ml). After the addition, the reaction mixture is allowed to warm to room temperature and stirred for 2 h. The solvent of the suspension is removed at reduced pressure and the residue is washed with Et_2O (3 x 1.5 ml). The colourless powder (**P4**$(OTf)_2$) is dried at reduced pressure (81.8 mg, 115 mmol, 70%).
Elemental analysis (%) for $C_{22}H_{40}N_8B_2F_6O_6S_2$ (712.26): calcd. C 37.09, H 5.66, N 15.73; found C 37.11, H 5.85, N 15.65. ^{1}H NMR (400 MHz, CD_3CN): δ = 7.15 – 7.13 (m, 2 H), 6.77 – 6.74 (m, 2 H), 3.07 (s, 24 H), 2.85 – 2.75 (br. s, 12 H) ppm. ^{13}C NMR (100 MHz, CD_3CN): δ = 163.03, 131.34, 125.08, 119.56, 41.98 ppm. ^{11}B NMR (128 MHz, CD_3CN): δ = 33.01 ppm.

Pure $\mathbf{P4}_{isomer}(OTf)_2$ is obtained by the quantitative isomerisation process at RT, redissolving **P4**$(OTf)_2$ in CH_3CN and stirring for 2 h.
^{1}H NMR (400 MHz, CD_3CN): δ = 7.41 – 7.39 (m, 2 H), 7.21 – 7.18 (m, 2 H), 3.32 (s, 12 H), 2.96 (s, 12 H), 2.75 – 2.50 (br. s, 12 H) ppm. ^{13}C NMR (100 MHz, CD_3CN): δ = 158.79, 133.70, 125.76, 115.59, 42.94, 42.59 ppm. ^{19}F NMR (376 MHz, CD_3CN): δ = 79.30 ppm. ^{11}B NMR (128 MHz, CD_3CN): δ = 37.72 (1 B), 30.51 (1 B) ppm. ^{11}B{^{1}H} NMR (128 MHz, CD_3CN): δ = 37.87 (1 B), 31.06 (1 B) ppm.

Fluoride catalysed isomerisation of **P4**$(OTf)_2$:
[18]-crown-6 (22.6 mg, 85.5 µmol) and KF (0.81 mg, 86 µmol) are suspended in CD_3CN (3 ml) for 24 h. The colourless residue (undissolved KF) is removed by filtration and 500 µl of the resulting solution is added to **P4**$(OTf)_2$ (10.0 mg, 14.3 µmol).

Isomerisation of **P4**$(GaCl_4)_2$ in CH_2Cl_2:
Due to the bad solubility of **P4**$(GaCl_4)_2$ in CH_2Cl_2 we were not able to follow the isomerisation process *in-situ*. Therefore, we estimated the $\mathbf{P4_{isomer}}$ amount by directly analysing a CD_3CN solution of **P4**$(GaCl_4)_2$ with ^1H NMR spectroscopy to be > 2%. Then, we stirred a suspension of **P4**$(GaCl_4)_2$ (15.0 mg, 17.9 µmol) in CH_2Cl_2 (2 ml) for 4 d and repeated the estimation of $\mathbf{P4_{isomer}}$ by ^1H NMR spectroscopy to be < 98%.

$\mathbf{P4_{isomer}F1}$$(GaCl_4)_2$:
KF (1.0 mg, 18 µmol) and 18-crown-6 (4.7 mg, 18 µmol) is added to a solution of $\mathbf{P4_{isomer}}$$(GaCl_4)_2$ (15.0 mg, 17.9 µmol) in CD_3CN (0.5 ml) and stirred for 18 h.
^1H NMR (400 MHz, CD_3CN): δ = 7.07 – 7.05 (m, 2 H), 6.80 – 6.77 (m, 2 H), 3.09 (s, 6 H), 2.95 (s, 6 H), 2.86 (s, 6 H), 2.85 – 2.80 (br. s, 6 H) ppm, 2.54 (s, 6 H) ppm, 2.47 (s, 6 H) ppm. ^{19}F NMR (376 MHz, CD_3CN): δ = –150.73 ppm. ^{11}B NMR (128 MHz, CD_3CN): δ = 36.06 (1 B), 7.42 (1 B) ppm. $^{11}B\{^1H\}$ NMR (128 MHz, CD_3CN): δ = 35.96 (1 B), 7.43(1 B) ppm.
HR ESI-MS for $C_{20}H_{40}B_2N_8F$: calc. 433.3546; exp. 433.3538.

We suggest the formation of $\mathbf{P4_{isomer}F1}$$(GaCl_4)$ after the addition of KF and [18]-crown-6 to $\mathbf{P4_{isomer}}$$(GaCl_4)$ according to the following reasons:

1) Complete consumption of $\mathbf{P4_{isomer}}$ as evinced by ^1H NMR spectroscopy.
2) The HR ESI-MS confirm the chemical formula $C_{20}H_{40}B_2N_8F$ (calc. 433.3546; exp. 433.3538).
3) ^{19}F NMR showing a broad signal at –150.73 ppm. This region is typical for B-F bonded fluoride atoms (e.g. BF_4^- = –154 ppm).
4) Two significant different ^{11}B NMR shifts (7.42 and 36.06 ppm) indicating two different bound boron atoms.
5) The calculated ^{11}B NMR of $\mathbf{P4_{isomer}F1}$ (10 and 37 ppm) fits mutch better to the experimental derived ^{11}B NMR signals (7.42 and 36.06 ppm) as the calculated one of $\mathbf{P4_{isomer}F2}$ (10 and 46 ppm) (for details, see Figure S32 and Table S3).
6) The calculated FIA of **B**(GFA) is 130 kJ mol^{-1} higher than **B**$(NMe_2)_2$ (see Figure S31).

3. Analytical data

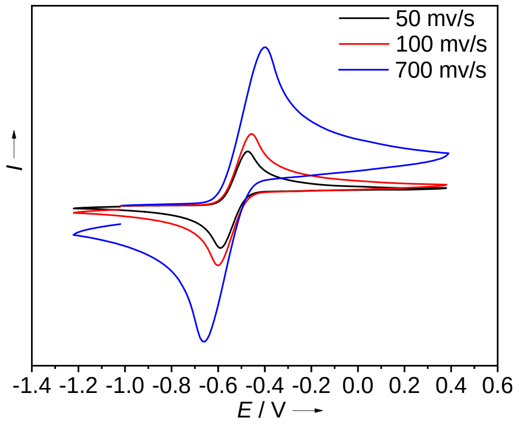

Figure S1: Comparison between the CV curves of **L2** in CH_2Cl_2 with different scan speeds. Ag/AgCl potentials given relative to the redox-couple Fc^+/Fc, Bu_4NPF_6 as supporting electrolyte.

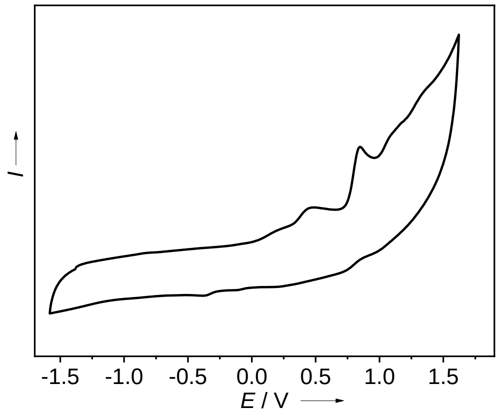

Figure S2: CV curve of **P1**$(OTf)_4$ in CH_3CN with 300 mv s^{-1} scan speed. Ag/AgCl potentials given relative to the redox-couple Fc^+/Fc, Bu_4NClO_4 as supporting electrolyte.

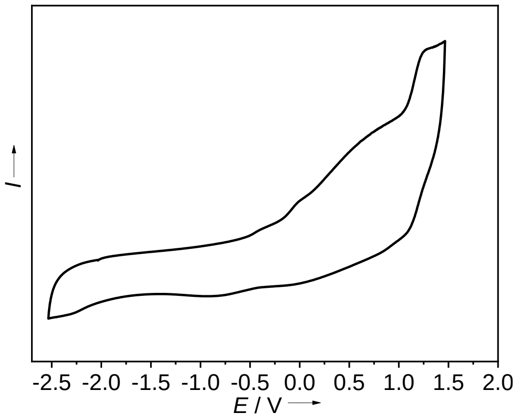

Figure S3: CV curve of $\mathbf{P2_{isomer}}(OTf)_2$ in CH_3CN with 300 mv s^{-1} scan speed. Ag/AgCl potentials given relative to the redox-couple Fc^+/Fc, Bu_4NClO_4 as supporting electrolyte.

The CV of **P1**$(OTf)_4$ (Figure S2) shows no reversible redox processes. Some irreversible oxidation processes were observed at $E_{ox}(1) = 0.19$; $E_{ox}(2) = 0.46$; $E_{ox}(3) = 0.85$; $E_{ox}(4) = 1.11$ and $E_{ox}(5) = 1.35$ V vs. Fc^+/Fc. However, no oxidation of Ligand **L1** is detectable which could be explained by the high charge of compound **P1**$(OTf)_4$, delocalized in the guanidine backbone of the ligand. Compound $\mathbf{P2_{isomer}}(OTf)_2$ shows a broad oxidation wave between –0.18 and 1.03 V vs Fc^+/Fc in the CV. Chemical

oxidation of **P2**$_{\mathbf{isomer}}$(OTf)$_2$ with 2 eq. $FcPF_6$ results in a deep green solution which is typical for **L2**$^{2+}$. Unfortunately, we were not able to analyse the reaction product. Further investigations are part of ongoing work.

X-ray crystallographic details

Full shells of intensity data were collected at low temperature with a Nonius Kappa CCD diffractometer (Mo-K_α radiation, sealed X-ray tube, graphite monochromator, CCDC 1979384 {**L2**}) and Bruker D8 Venture, dual source (Mo- or Cu-K_α radiation, microfocus X-ray tube, Photon III detector, CCDC 1979389 [**L1**$GaCl_2$]($GaCl_4$)$_2$, 1979390 **P1**(OTf)$_2$, 1979386 **P2**$_{isomer}$($GaCl_4$)$_2$, 1979394 **P2**$_{isomer}$($AlCl_4$)$_2$, 1979385 **P3**(OTf)$_2$, 1979388 **P4**($GaCl_4$)$_2$, 1979387 **P4**$_{isomer}$($GaCl_4$)$_2$, and 1979391 **P4**$_{isomer}$(OTf)$_2$,). Data were processed with the standard Nonius and Bruker (SAINT, APEX3) software package.[5] Multiscan absorption correction was applied using the SADABS program.[6] The structures were solved by intrinsic phasing[7] and refined using the SHELXTL software package (Version 2014/6 and 2018/3).[8] Graphical handling of the structural data during solution and refinement were performed with OLEX2.[9] All non-hydrogen atoms were given anisotropic displacement parameters. Hydrogen atoms bound to carbon were input at calculated positions and refined with a riding model. Hydrogen atoms bound to nitrogen were located in difference Fourier syntheses and refined, either fully or with appropriate distance and/or symmetry.

Due to severe disorder and fractional occupancy, electron density attributed to solvent of crystallization (dichloromethane) was removed from the structure of **P3**(OTf)$_2$ with the BYPASS procedure,[10] as implemented in PLATON (squeeze/hybrid).[11] Partial structure factors from the solvent masks were included in the refinement as separate contributions to F_{calc}.

Crystal data for [**L1**($GaCl_2$)$_2$]($GaCl_4$)$_2$:

Formula	$C_{28}H_{53}Cl_{12}Ga_4N_{13}$
$D_{calc.}$/ g cm^{-3}	1.624
μ/mm^{-1}	2.694
Formula Weight	1276.11
Colour	clear colourless
Shape	block
Size/mm^3	0.45×0.31×0.24
T/K	100
Crystal System	monoclinic
Space Group	$P2_1/c$
a/Å	23.7371(13)
b/Å	16.9012(8)
c/Å	13.0469(5)
α/°	90
β/°	94.324(2)
γ/°	90
V/Å^3	5219.3(4)
Z	4
Z'	1
Wavelength/Å	0.71073
Radiation type	MoK_α
Θ_{min}/°	1.975
Θ_{max}/°	30.538
Measured Refl.	225023
Independent Refl.	15968
Reflections with I > 2(I)	14348
R_{int}	0.0572
Parameters	532
Restraints	0
Largest Peak	0.759
Deepest Hole	-0.558
GooF	1.026
wR_2 (all data)	0.0544
wR_2	0.0522
R_1 (all data)	0.0271
R_1	0.0224

Crystal data for **P1**(OTf)$_4$:

Formula	$C_{40}H_{77}B_4F_{12}N_{17}O_{12}S_4$
$D_{calc.}$/ g cm^{-3}	1.308
μ/mm^{-1}	0.227
Formula Weight	1387.66
Colour	clear light colourless
Shape	plate
Size/mm^3	0.25×0.23×0.16
T/K	100
Crystal System	monoclinic
Space Group	$P2_1/n$
a/Å	10.4576(7)
b/Å	26.719(2)
c/Å	25.7325(18)
α/°	90
β/°	101.353(2)
γ/°	90
V/Å^3	7049.3(9)
Z	4
Z'	1
Wavelength/Å	0.71073
Radiation type	MoK$_\alpha$
Θ_{min}/°	1.991
Θ_{max}/°	25.998
Measured Refl.	496206
Independent Refl.	13838
Reflections with I > 2(I)	11390
R_{int}	0.0850
Parameters	920
Restraints	210
Largest Peak	1.530
Deepest Hole	-1.006
GooF	1.064
wR_2 (all data)	0.2736
wR_2	0.2613
R_1 (all data)	0.1084
R_1	0.0954

Crystal data for **L2**:

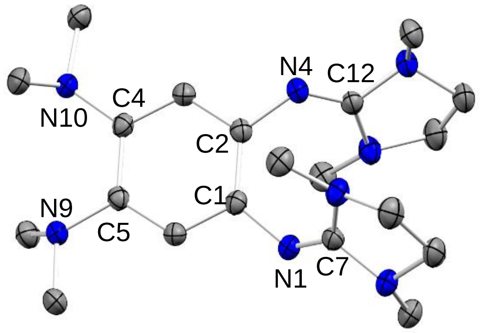

C1–C2	1.409(2) Å
N1–C7	1.279(2) Å
N4–C12	1.279(2) Å
C4–N10	1.432(2) Å
C5–N9	1.432(2) Å

*Only one of two molecules in the unit cell is shown.

Formula	$C_{20}H_{34}N_8$
$D_{calc.}$/ g cm^{-3}	1.242
μ/mm^{-1}	0.079
Formula Weight	386.55
Colour	colourless
Shape	box shaped
Size/mm^3	0.90×0.70×0.40
T/K	120
Crystal System	monoclinic
Space Group	$P2/n$
a/Å	13.133(3)
b/Å	12.028(2)
c/Å	13.881(3)
α/°	90
β/°	109.47(3)
γ/°	90
V/Å^3	2067.3(8)
Z	4
Z'	1
Wavelength/Å	0.71073
Radiation type	MoK_α
Θ_{min}/°	1.693
Θ_{max}/°	30.054
Measured Refl.	34048
Independent Refl.	6041
Reflections with I > 2(I)	4043
R_{int}	0.0582
Parameters	261
Restraints	0
Largest Peak	0.295
Deepest Hole	-0.243
GooF	1.023
wR_2 (all data)	0.1364
wR_2	0.1173
R_1 (all data)	0.0887
R_1	0.0489

Crystal data for **P2**$_{\mathbf{isomer}}$$(GaCl_4)_2$:

Formula	$C_{24}H_{46}B_2Cl_8Ga_2N_{10}$
$D_{calc.}$/ g cm^{-3}	1.506
μ/mm^{-1}	1.887
Formula Weight	919.37
Colour	clear colourless
Shape	block
Size/mm^3	0.44×0.34×0.28
T/K	100(2)
Crystal System	monoclinic
Space Group	$P2_1/n$
a/Å	11.0650(3)
b/Å	14.1530(5)
c/Å	25.9003(9)
α/°	90
β/°	90.7010(10)
γ/°	90
V/Å^3	4055.8(2)
Z	4
Z'	1
Wavelength/Å	0.71073
Radiation type	MoK_α
Θ_{min}/°	2.010
Θ_{max}/°	30.516
Measured Refl.	197578
Independent Refl.	12376
Reflections with I > 2(I)	10275
R_{int}	0.0595
Parameters	436
Restraints	0
Largest Peak	1.069
Deepest Hole	-1.002
GooF	1.026
wR_2 (all data)	0.0679
wR_2	0.0630
R_1 (all data)	0.0412
R_1	0.0292

Crystal data for **P2**$_{\mathbf{isomer}}$($AlCl_4$)$_2$:

Formula	$C_{24}H_{46}B_2Cl_8Al_2N_{10}$
$D_{calc.}$/ g cm^{-3}	1.363
μ/mm^{-1}	5.747
Formula Weight	833.89
Colour	clear colourless
Shape	block
Size/mm^3	0.47×0.34×0.20
T/K	100(2)
Crystal System	monoclinic
Space Group	$P2_1/n$
a/Å	11.0819(3)
b/Å	14.2094(4)
c/Å	25.7991(7)
α/°	90
β/°	90.649(2)
γ/°	90
V/Å^3	4062.25(19)
Z	4
Z'	1
Wavelength/Å	1.54178
Radiation type	CuK$_\alpha$
Θ_{min}/°	3.43
Θ_{max}/°	78.56
Measured Refl.	154814
Independent Refl.	7320
Reflections with I > 2(I)	6778
R_{int}	0.0952
Parameters	454
Restraints	1
Largest Peak	1.194
Deepest Hole	-0.992
GooF	1.009
wR_2 (all data)	0.2262
wR_2	0.2242
R_1 (all data)	0.1104
R_1	0.1059

Crystal data for **P3**[OTf]$_2$:

Formula	$C_{38}H_{62}B_2Cl_4F_6N_{14}O_6S_2$
$D_{calc.}$/ g cm^{-3}	1.412
μ/mm^{-1}	0.372
Formula Weight	1152.55
Colour	clear light colourless
Shape	block
Size/mm^3	0.47×0.37×0.31
T/K	100
Crystal System	triclinic
Space Group	P-1
a/Å	12.9899(6)
b/Å	13.3562(6)
c/Å	17.7091(8)
α/°	107.063(2)
β/°	102.893(2)
γ/°	103.524(2)
V/Å^3	2711.4(2)
Z	2
Z'	1
Wavelength/Å	0.71073
Radiation type	MoK$_\alpha$
Θ_{min}/°	1.971
Θ_{max}/°	27.498
Measured Refl.	64544
Independent Refl.	12408
Reflections with I > 2(I)	10822
R_{int}	0.0307
Parameters	675
Restraints	3
Largest Peak	0.849
Deepest Hole	-0.924
GooF	1.029
wR_2 (all data)	0.1336
wR_2	0.1268
R_1 (all data)	0.0546
R_1	0.0474

Crystal data for **P4**($GaCl_4$)$_2$:

Formula	$C_{20}H_{40}B_2Cl_8Ga_2N_8$
$D_{calc.}$/ g cm^{-3}	1.548
μ/mm^{-1}	2.122
Formula Weight	837.26
Colour	clear light colourless
Shape	block
Size/mm^3	0.81×0.41×0.34
T/K	100
Crystal System	triclinic
Space Group	P-1
a/Å	8.3951(4)
b/Å	12.0972(6)
c/Å	19.3347(9)
α/°	102.012(2)
β/°	91.465(2)
γ/°	109.920(2)
V/Å^3	1795.71(15)
Z	2
Z'	1
Wavelength/Å	0.71073
Radiation type	MoK$_\alpha$
Θ_{min}/°	1.908
Θ_{max}/°	28.745
Measured Refl.	91229
Independent Refl.	9254
Reflections with I > 2(I)	8858
R_{int}	0.0485
Parameters	374
Restraints	0
Largest Peak	0.802
Deepest Hole	-0.954
GooF	1.029
wR_2 (all data)	0.0530
wR_2	0.0523
R_1 (all data)	0.0224
R_1	0.0212

Crystal data for **P4**$_{isomer}$($GaCl_4$)$_2$:

Formula	$C_{22}H_{43}B_2Cl_8Ga_2N_9$
$D_{calc.}$/ g cm^{-3}	1.496
μ/mm^{-1}	1.959
Formula Weight	878.31
Colour	clear colourless
Shape	plate
Size/mm^3	0.31×0.14×0.11
T/K	100(2)
Crystal System	monoclinic
Space Group	$P2_1/c$
a/Å	21.8640(6)
b/Å	12.9944(3)
c/Å	14.2561(3)
α/°	90
β/°	105.6940(10)
γ/°	90
V/Å^3	3899.30(16)
Z	4
Z'	1
Wavelength/Å	0.71073
Radiation type	MoK_α
Θ_{min}/°	1.935
Θ_{max}/°	30.515
Measured Refl.	133894
Independent Refl.	11921
Reflections with I > 2(I)	9964
R_{int}	0.0520
Parameters	401
Restraints	0
Largest Peak	0.761
Deepest Hole	-0.682
GooF	1.040
wR_2 (all data)	0.0612
wR_2	0.0566
R_1 (all data)	0.0390
R_1	0.0276

Crystal data for **P4$_{isomer}$**(OTf)$_2$:

Formula	$C_{24}H_{43}B_2F_6N_9O_6S_2$
$D_{calc.}$/ g cm^{-3}	1.422
μ/mm^{-1}	0.234
Formula Weight	753.41
Colour	clear light colourless
Shape	block
Size/mm^3	0.21×0.16×0.12
T/K	106(2)
Crystal System	orthorhombic
Flack Parameter	0.01(3)
Space Group	$P2_12_12_1$
a/Å	12.3737(8)
b/Å	14.5285(8)
c/Å	19.5802(12)
α/°	90
β/°	90
γ/°	90
V/Å^3	3520.0(4)
Z	4
Z'	1
Wavelength/Å	0.71073
Radiation type	MoK$_\alpha$
Θ_{min}/°	1.947
Θ_{max}/°	28.322
Measured Refl.	89666
Independent Refl.	8752
Reflections with I > 2(I)	7556
R_{int}	0.1039
Parameters	455
Restraints	0
Largest Peak	0.251
Deepest Hole	-0.359
GooF	1.092
wR_2 (all data)	0.0886
wR_2	0.0795
R_1 (all data)	0.0536
R_1	0.0409

4. DFT calculations:

All quantum-chemical computations in the following part rely on the Turbomole V7.2.1 suite of programs or newer.[S12] Structural optimizations were performed at the RI-DFT[S13] level of theory with multipole accelerated RI-approximation (MARI-J)[S14] using the B3LYP[S15, S16] functional in combination with the def2-TZVP [S17] basis sets. Dispersion is included by the DFT-D3[S18] approach developed by Grimme and co-workers. All calculated geometries have been confirmed as energetic minima on the potential energy surface by analytical calculation of harmonic frequencies, revealing only positive values. As seen in the following, it figured out that the combination of the functional B3LYP+D3 with the def2-TZVP basis set describes all structure (**P1**, **P2**, **P4**, **P5**, $\mathbf{P1_{isomer}}$, $\mathbf{P2_{isomer}}$, $\mathbf{P4_{isomer}}$, $\mathbf{P5_{isomer}}$) in good to very good agreement (see below). For the calculations of relative free energies by considering the solvent effect, conductor-like screening model (COSMO)[S19] is used by means of single-point calculations (CH_3CN = 37.50). The deviation between the experimentally derived and calculated structures are analysed with the program aRMSD[S20] using default settings. The isodensity plots were obtained from the B3LYP/def2-TZVP output and rendered with IboView with a density threshold of 80.0 units.

The crystal structure of **P1**$_{isomer}$(OTf)$_4$ is in good agreement with that calculated, with a total RMSD of 0.47 Å. Deviations are due to twists of NMe$_2$ groups of the guanidine (Figure S4). All bond distances are in excellent agreement, as evinced with the RMSE of 0.011 Å (Figure S5).

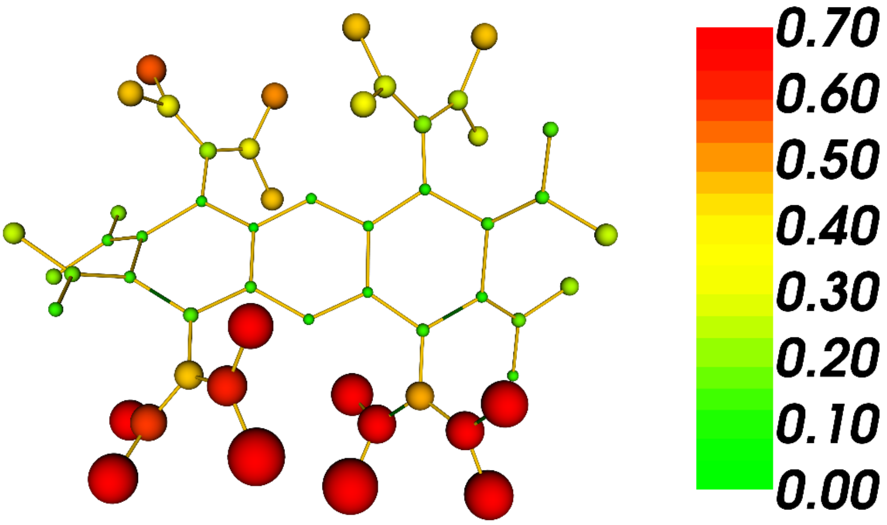

Figure S4: Superposition with Root-Mean-Square-Deviation (RMSD) for the experimentally determined structure of **P4**$_{isomer}$$^{4+}$ and the calculated structure. The sphere dimensions reflect the relative RMSD distribution and the color code the absolute deviation (small for green color and large for red color).

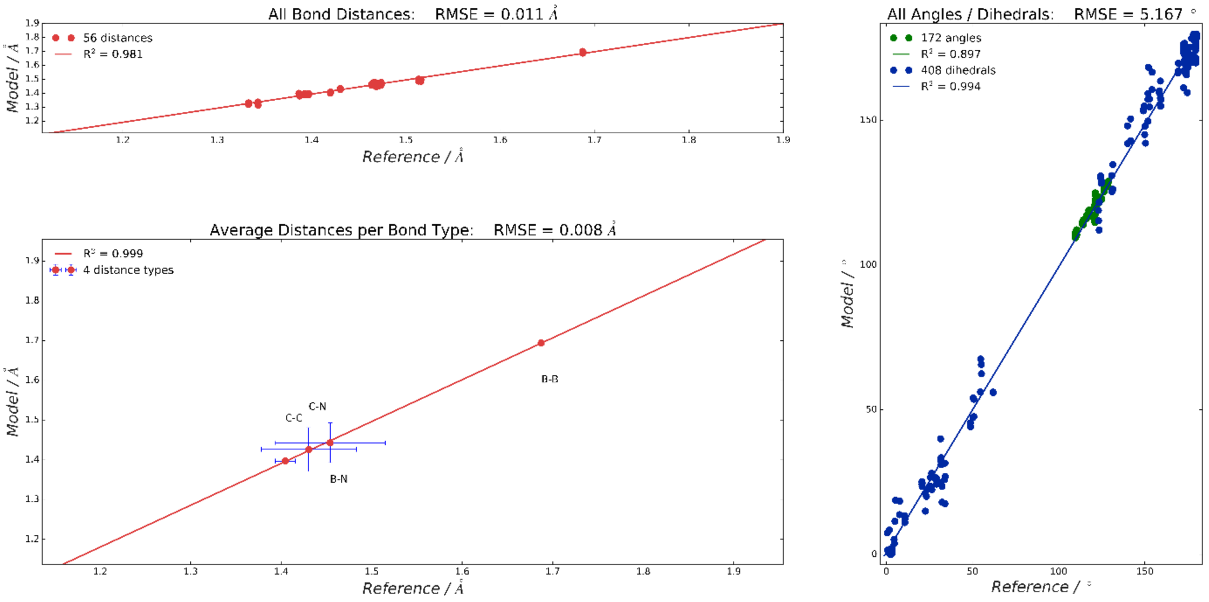

Figure S5: Detailed analysis of the Root-Mean-Square-Error (RMSE) for bonds and angles for the experimentally determined structure (Model) of **P4**$_{isomer}$$^{4+}$ and the calculated structure (Reference).

The crystal structure of **P2**$_{\textbf{isomer}}$($GaCl_4$)$_2$ is in very good agreement with that calculated, with a total RMSD of 0.16 Å. Only small deviations are due to twists of NMe_2 groups of the guanidine and NMe_2 groups which are directly bound to the boron atom (Figure S6). All bond distances are in excellent agreement, as evinced with the RMSE of 0.008 Å (Figure S7).

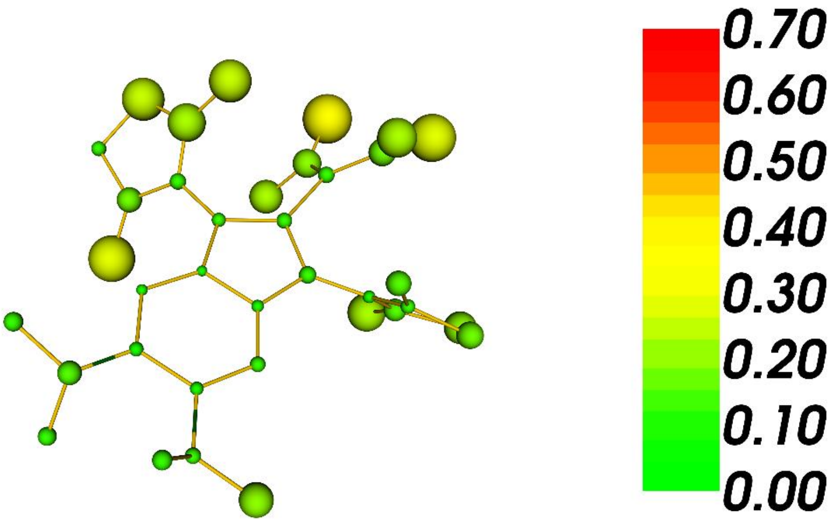

Figure S6: Superposition with Root-Mean-Square-Deviation (RMSD) for the experimentally determined structure of **P2**$_{\textbf{isomer}}^{2+}$ and the calculated structure. The sphere dimensions reflect the relative RMSD distribution and the color code the absolute deviation (small for green color and large for red color).

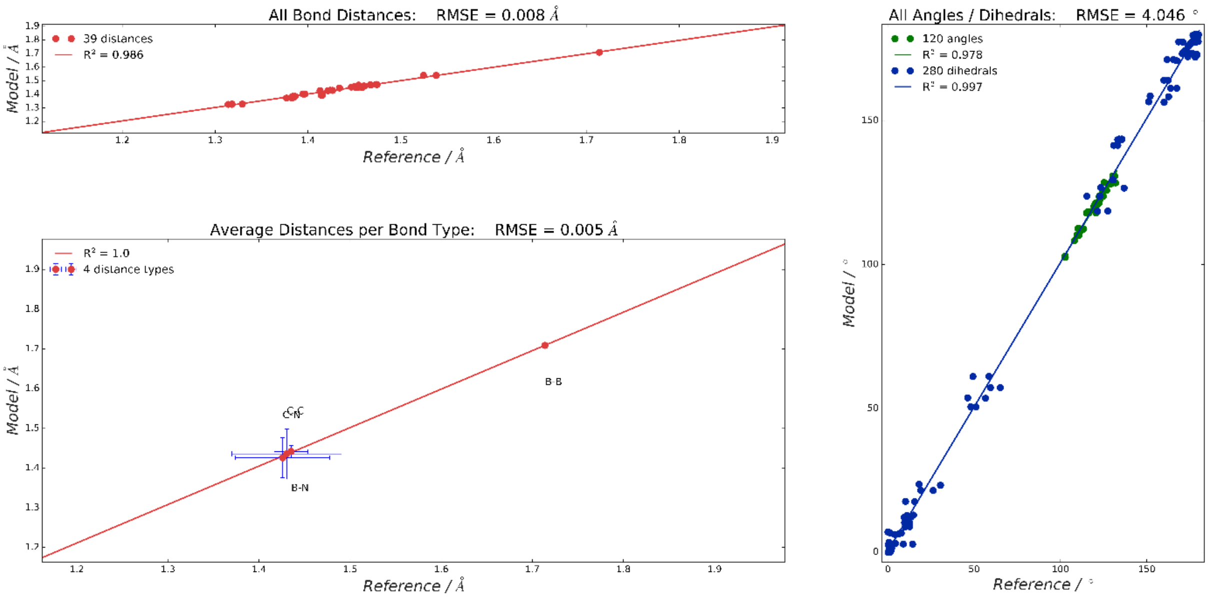

Figure S7: Detailed analysis of the Root-Mean-Square-Error (RMSE) for bonds and angles for the experimentally determined structure (Model) of **P2**$_{\textbf{isomer}}^{2+}$ and the calculated structure (Reference).

The crystal structure of **P4**$_{\textbf{isomer}}$($GaCl_4$)$_2$ is in very good agreement with that calculated, with a total RMSD of 0.12 Å. Only small deviations are due to twists of NMe_2 groups of the guanidine or the benzene ring (Figure S8). All bond distances are in excellent agreement, as evinced with the RMSE of 0.005 Å (Figure S9).

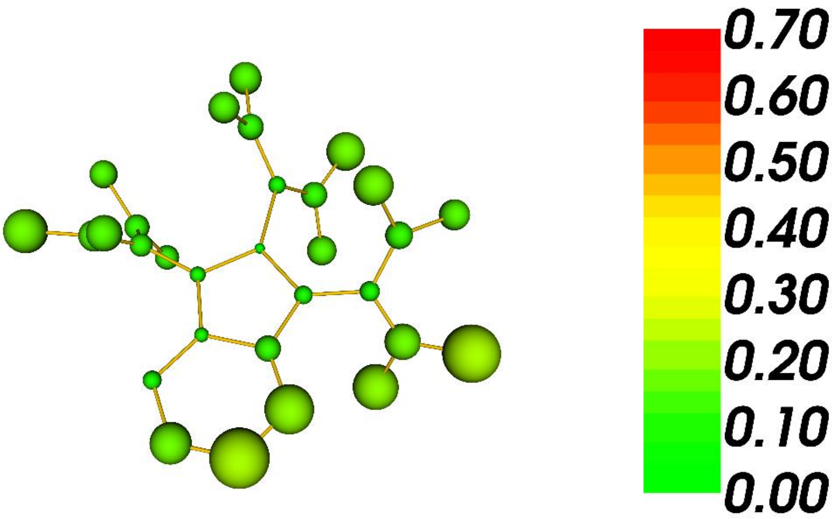

Figure S8: Superposition with Root-Mean-Square-Deviation (RMSD) for the experimentally determined structure of **P4**$_{\textbf{isomer}}^{2+}$ and the calculated structure. The sphere dimensions reflect the relative RMSD distribution and the color code the absolute deviation (small for green color and large for red color).

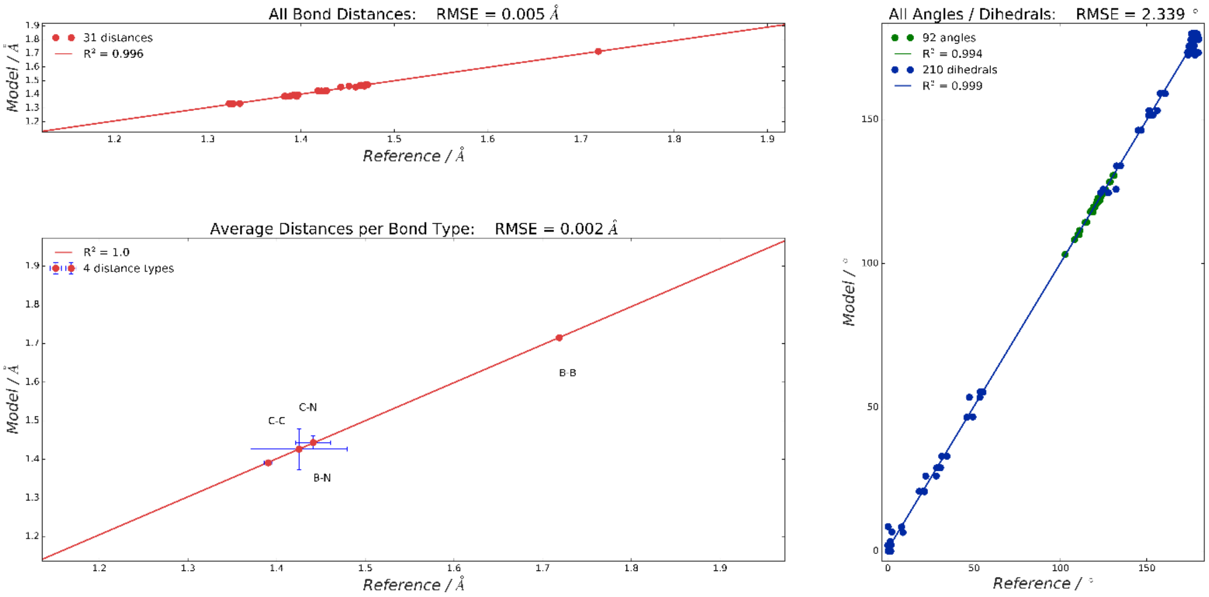

Figure S9: Detailed analysis of the Root-Mean-Square-Error (RMSE) for bonds and angles for the experimentally determined structure (Model) of **P4**$_{\textbf{isomer}}^{2+}$ and the calculated structure (Reference).

The crystal structure of **P4**($GaCl_4$)$_2$ is in very good agreement with that calculated, with a total RMSD of 0.36 Å. Only small deviations are due to twists of NMe_2 groups or the benzene ring (Figure S8). All bond distances are in excellent agreement, as evinced with the RMSE of 0.003 Å (Figure S9).

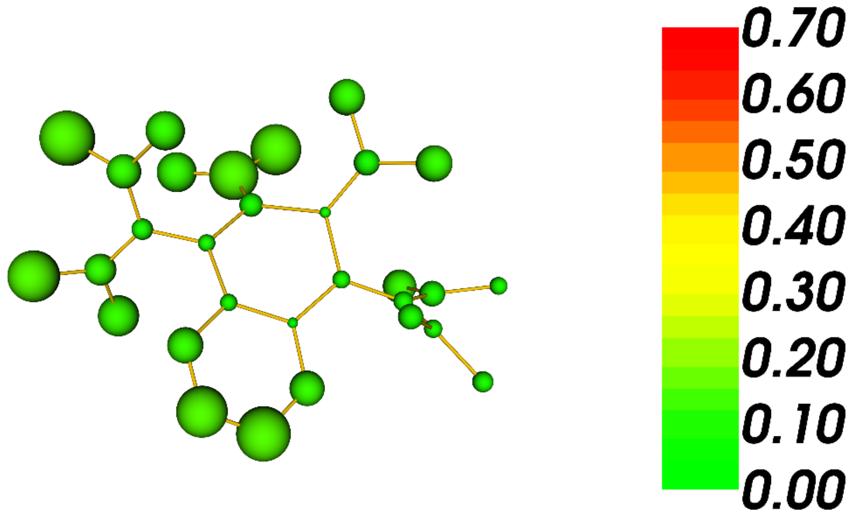

Figure S10: Superposition with Root-Mean-Square-Deviation (RMSD) for the experimentally determined structure of **P4**$^{2+}$ and the calculated structure. The sphere dimensions reflect the relative RMSD distribution and the color code the absolute deviation (small for green color and large for red color).

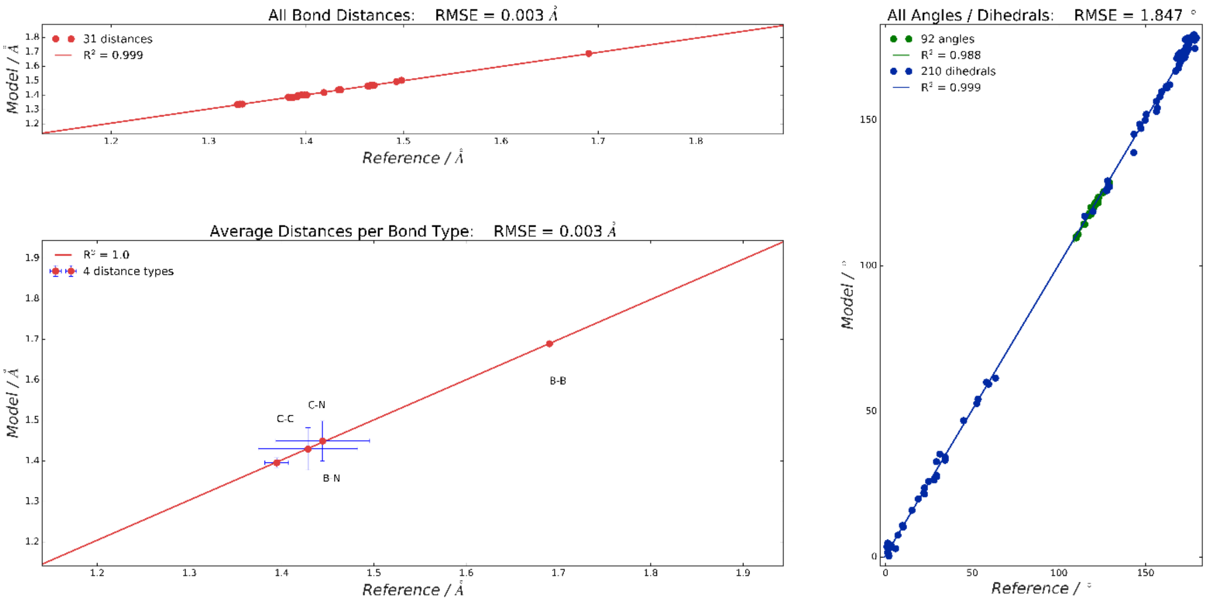

Figure S11: Detailed analysis of the Root-Mean-Square-Error (RMSE) for bonds and angles for the experimentally determined structure (Model) of **P4**$^{2+}$ and the calculated structure (Reference).

The crystal structure of **5**$(SbCl_6)_2$[S21] displays some deviations to that calculated. The total RMSD is 0.63 Å. The deviations are mainly due to twists of the mesityl-groups (Figure S12). Mainly, all bond distances are in good agreement, as evinced with the RMSE of 0.012 Å (Figure S13).

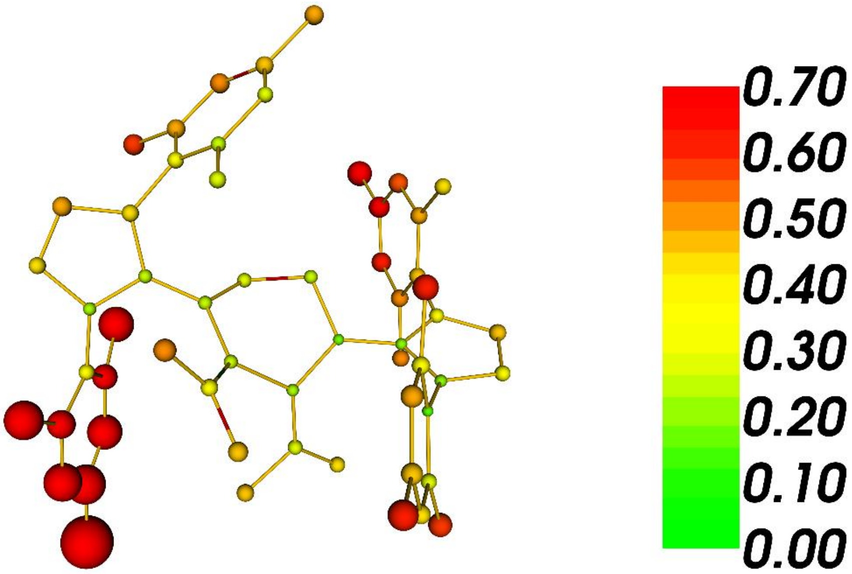

Figure S12: Superposition with Root-Mean-Square-Deviation (RMSD) for the experimentally determined structure of $\mathbf{5}^{2+}$ and the calculated structure. The sphere dimensions reflect the relative RMSD distribution and the color code the absolute deviation (small for green color and large for red color).

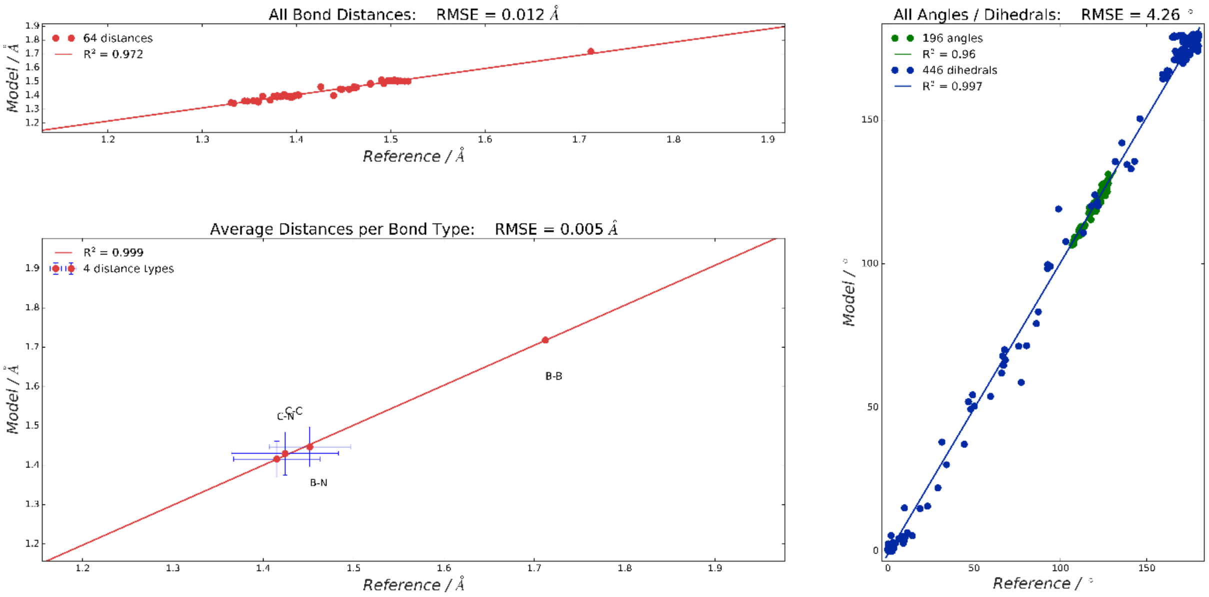

Figure S13: Detailed analysis of the Root-Mean-Square-Error (RMSE) for bonds and angles for the experimentally determined structure (Model) of $\mathbf{5}^{2+}$ and the calculated structure (Reference).

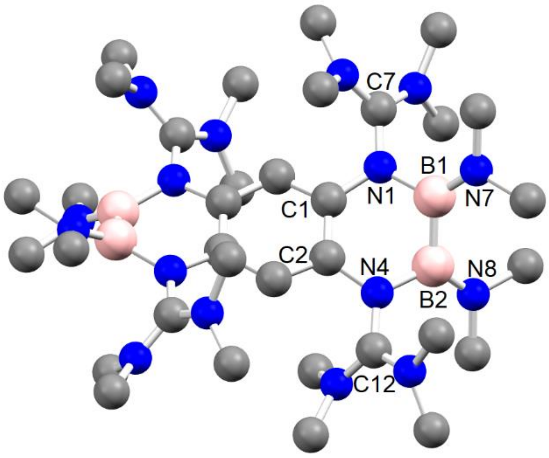

Figure S14: Illustration of the calculated structure of **P1**. E_{tot} = -2314.1119023480 H; Chem pot = 2713.84 kJ mol^{-1} (1 bar, 298 K).

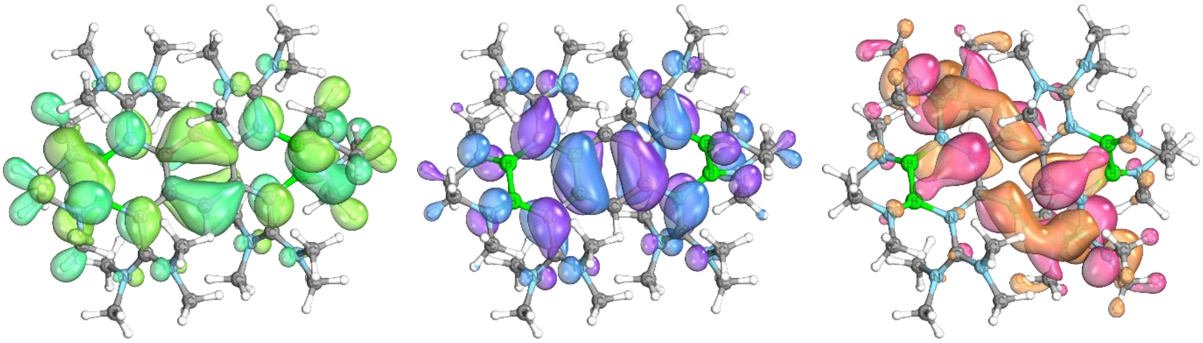

HOMO-1 (–15.639 eV) HOMO (–14.917 eV) LUMO (–10.983 eV)

Figure S15: Frontier molecular orbital plots for **P1**.

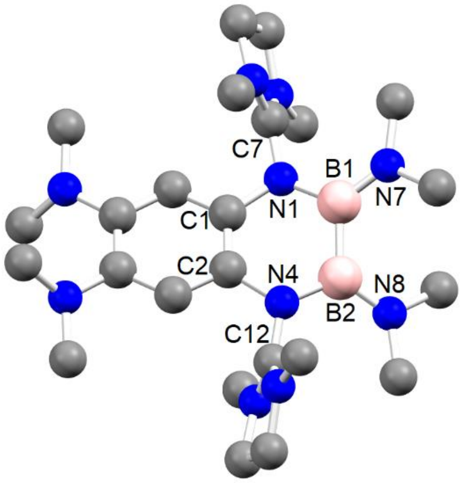

Figure S16: Illustration of the calculated structure of **P2**. E_{tot} = -1538.7690997020 H; Chem pot = 1689.48 kJ mol^{-1} (1 bar, 298 K).

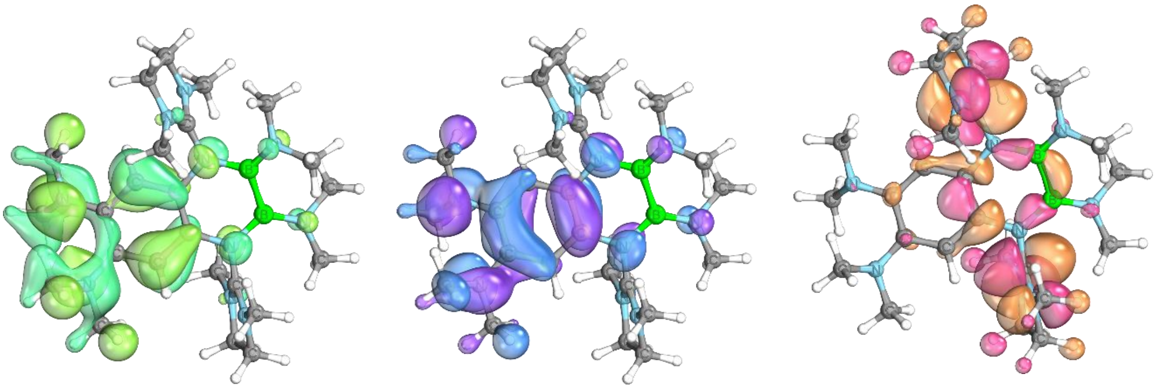

HOMO-1 (–10.604 eV) HOMO (–9.811 eV) LUMO (–6.368 eV)

Figure S17: Frontier molecular orbital plots for **P2**.

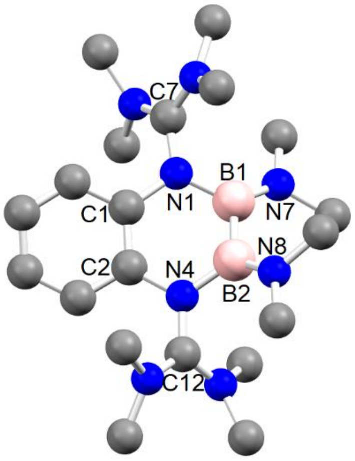

Figure S18: Illustration of the calculated structure of **P4**. E_{tot} = -1273.2770951720 H; Chem pot = 1448.72 kJ mol^{-1} (1 bar, 298 K).

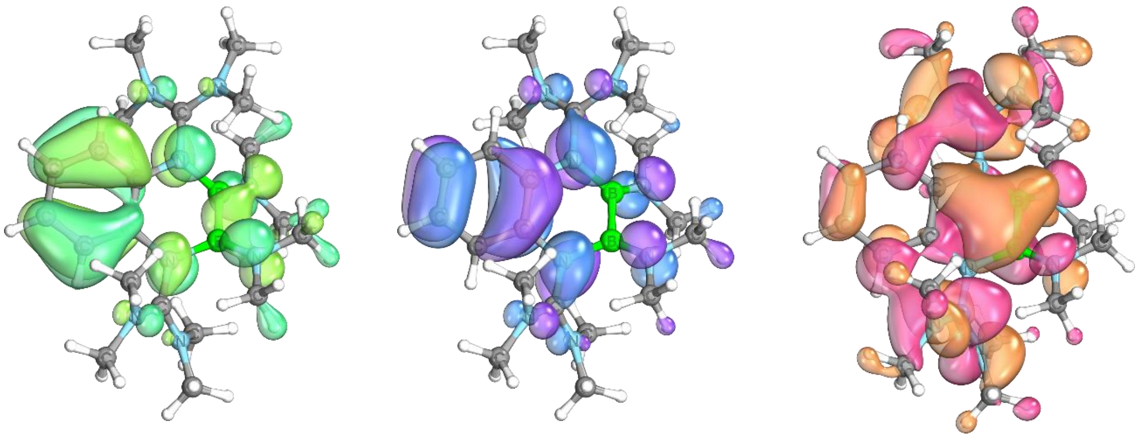

HOMO-1 (–10.898 eV) HOMO (–10.616 eV) LUMO (–7.751 eV)

Figure S19: Frontier molecular orbital plots for **P4**.

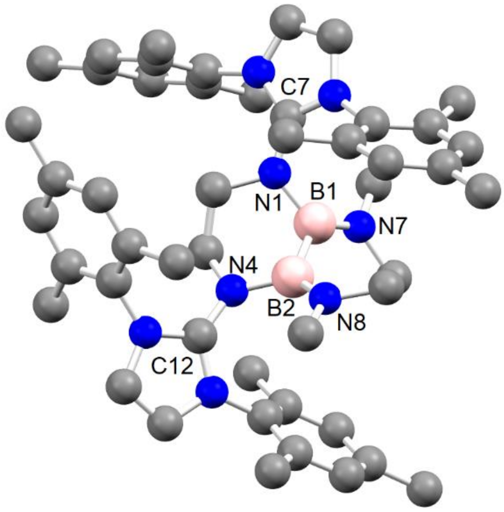

Figure S20: Illustration of the calculated structure of **5**. E_{tot} = -2354.5736802460 H; Chem pot = 2483.34 kJ mol^{-1} (1 bar, 298 K).

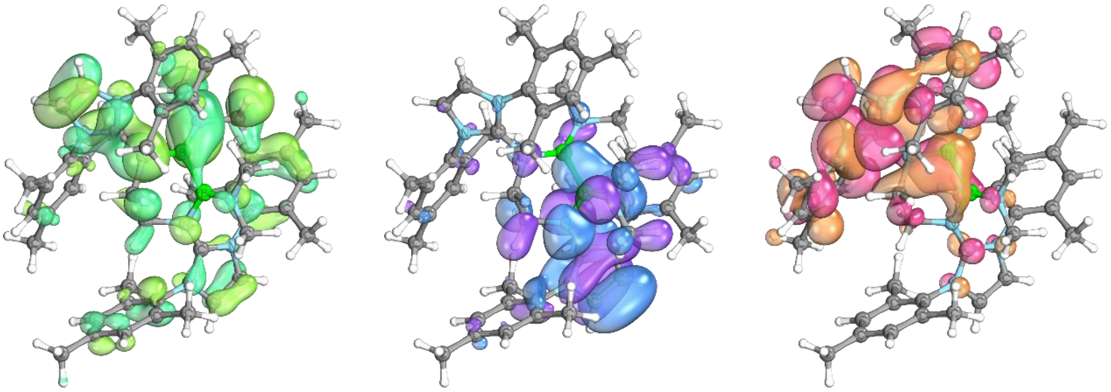

HOMO-1 (–11.112 eV) HOMO (–10.997 eV) LUMO (–5.965 eV)

Figure S21: Frontier molecular orbital plots for **5**.

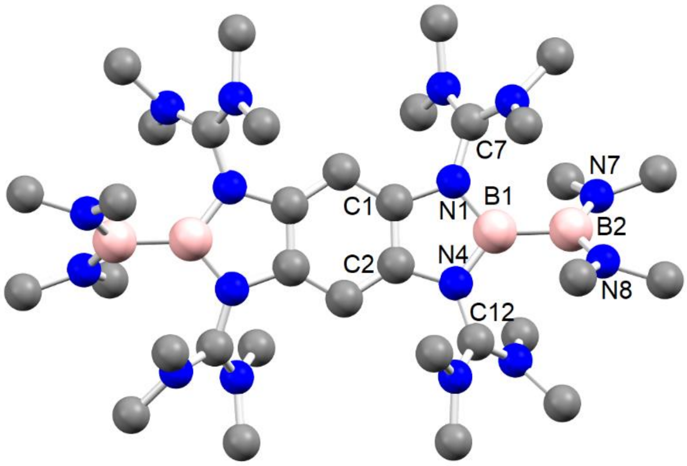

Figure S22: Illustration of the calculated structure of **P1$_{isomer}$**. E_{tot} = -2314.1492963600 H; Chem pot = 2707.55 kJ mol^{-1} (1 bar, 298 K).

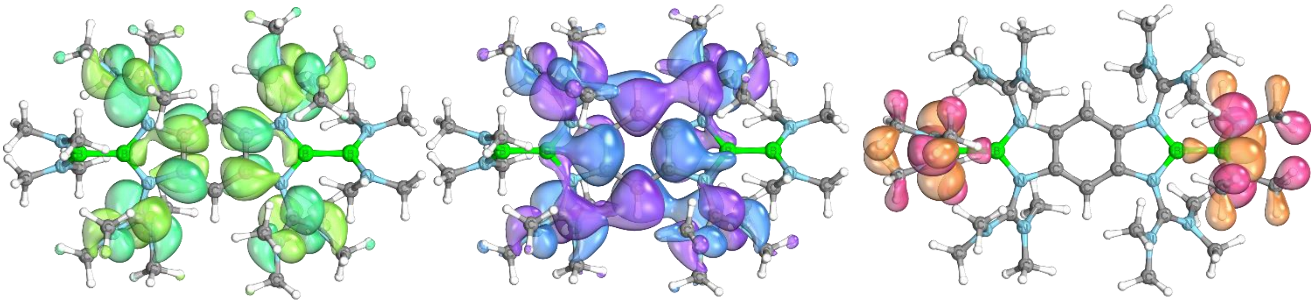

HOMO-1 (–14.325 eV) HOMO (–14.321 eV) LUMO (–11.100 eV)

Figure S23: Frontier molecular orbital plots for **P1**$_{\mathbf{Isomer}}$.

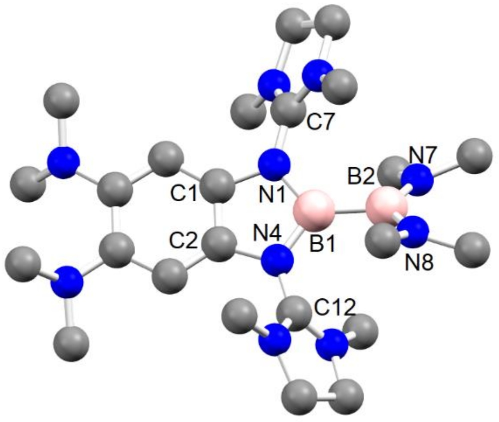

Figure S24: Illustration of the calculated structure of **P2**$_{\mathbf{isomer}}$. E_{tot} = -1538.7852830310 H; Chem pot = 1688.53 kJ mol^{-1} (1 bar, 298 K).

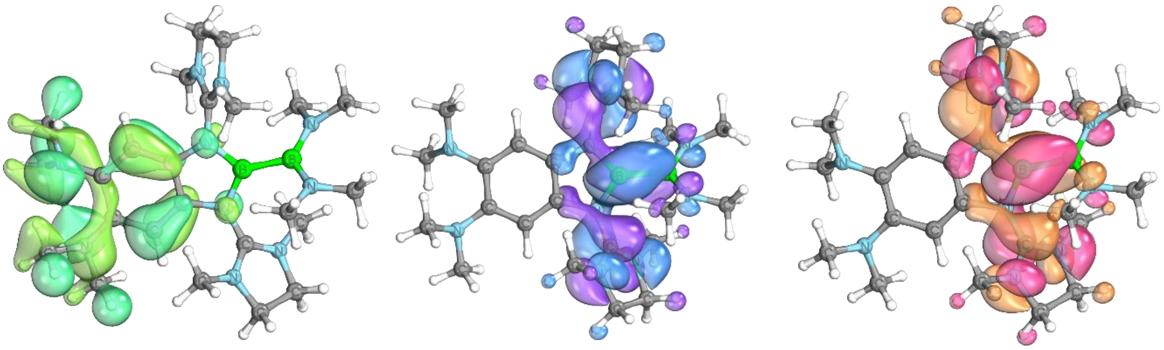

HOMO-1 (–10.667 eV) HOMO (–9.871 eV) LUMO (–6.598 eV)

Figure S25: Frontier molecular orbital plots for **P2**$_{\mathbf{Isomer}}$.

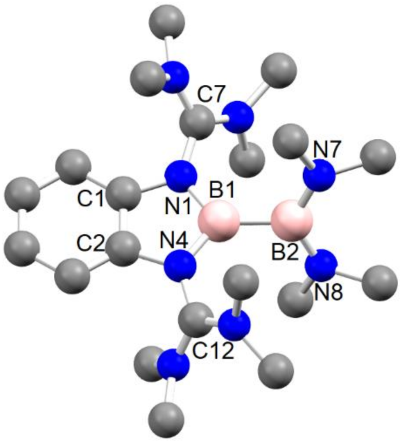

Figure S26: Illustration of the calculated structure of **P4**$_{\mathbf{isomer}}$. E_{tot} = -1273.2959138350 H; Chem pot = 1448.93 kJ mol^{-1} (1 bar, 298 K).

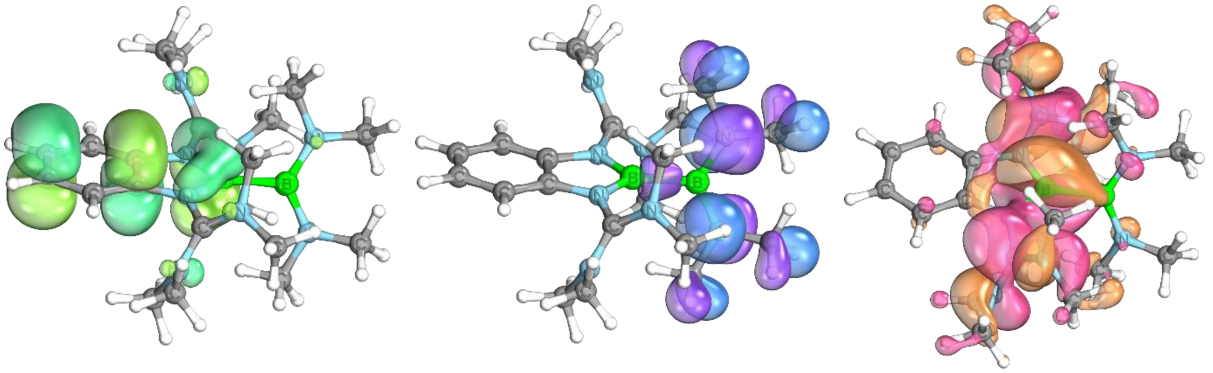

HOMO-1 (–11.442 eV) HOMO (–10.368 eV) LUMO (–7.943 eV)

Figure S27: Frontier molecular orbital plots for **P4**$_{\mathbf{Isomer}}$.

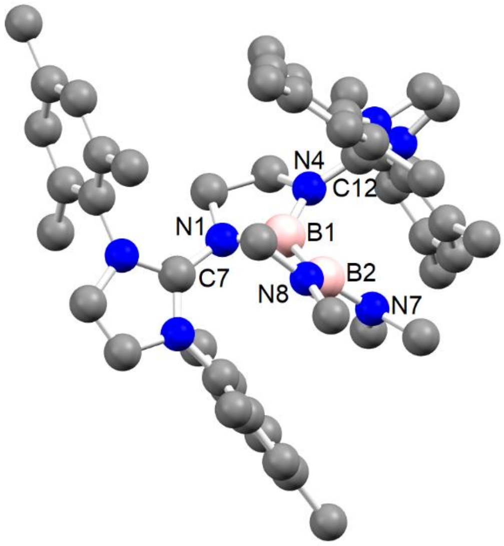

Figure S28: Illustration of the calculated structure of **5**$_{\mathbf{isomer}}$. E_{tot} = -2354.5696867710 H; Chem pot = 2477.84 kJ mol^{-1} (1 bar, 298 K).

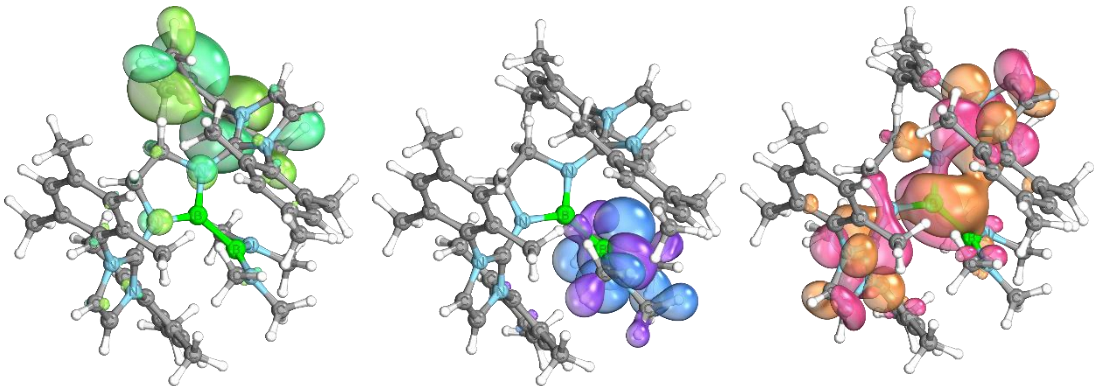

HOMO-1 (–11.259 eV) HOMO (–10.294 eV) LUMO (–6.112 eV)

Figure S29: Frontier molecular orbital plots for **5**.

Table S1: Selected bond legths (in Å) of the calculated structures of the diboranes.

	**P1**	**P2**	**P4**	**5**
B1–B2	1.688	1.710	1.689	1.718
B1–N1	1.516	1.481	1.503	1.482
B1–N4	1.514	1.481	1.494	1.511
B2–N7	1.393	1.409	1.399	1.400
B2–N8	1.394	1.409	1.402	1.394
C1–C2	1.420	1.393	1.418	-
N1–C7	1.387	1.386	1.385	1.366
N4–C12	1.387	1.385	1.388	1.352

	**P1**$_{isomer}$	**P2**$_{isomer}$	**P4**$_{isomer}$	**5**$_{isomer}$
B1–B2	1.721	1.709	1.715	1.721
B1–N1	1.466	1.457	1.461	1.461
B1–N4	1.466	1.457	1.461	1.458
B2–N7	1.424	1.427	1.426	1.434
B2–N8	1.424	1.427	1.426	1.419
C1–C2	1.405	1.382	1.396	-
N1–C7	1.396	1.372	1.385	1.368
N4–C12	1.396	1.372	1.385	1.369

DFT calculations on the bonding mode of $P4_{isomer}$:

Geometries:

Geometry optimizations have been performed with ORCA 4.2,[S22] at the PBEh-3c/def2-mSVP level of theory.[S23] The RI approximation[S24] for the Coulomb integrals was used in all cases (RIJCOSX), with application of corresponding auxiliary basis sets.[S25] All calculated geometries have been confirmed as energetic minima on the potential energy surface by analytical calculation of harmonic frequencies, revealing only positive values. To describe the bonding mode in **$P4_{isomer}$**, we tested different functionals to get the best possible structure. Herein, especially the PBEh-3c functional figured out to be in perfect agreement with the SCXRD derived structure, as can be seen in the following table.

Table S2: Comparison of selected computed and measured bond lengths and angles (in Å/°).

**$P4_{Isomer}$**	**SCXRD**	**[PBEh-3c]**	**[B3LYP-D3/def2TZVP]**
B-B	1.719(2)	1.716	1.714
B-NMe_2	1.418/1.427(2)	1.419/1.420	1.425
B-N-C(NR)$_2$Ar	1.451/1.468(2)	1.456	1.461/1.462
Me_2N-C	1.323 – 1.334(2)	1.324 – 1.327	1.328 – 1.334
(Me_2N)2C-N-B	1.395 – 1.384(2)	1.379 – 1.376	1.381 – 1.384
N-B-B-N (torsion angle)	55.2	56.5	50.6
**TDADB**			
B-B	1.735(3)	1.730	1.727
B-NMe_2	1.422(3) – 1.430(3)	1.430	1.435
N-B-B-N (torsion angle)	60.5	65.7	77.8

Single point energies, FIA, calculated NMR shifts and bond dissociation enthalpies

The PW6B95[26] including Grimme's semi-empirical dispersion correction[S18] with Becke-Johnson damping function[S27] (D3(BJ) and the def2-QZVPP[S28] basis set was used for the final single-point evaluation. With a large quadruple-ζ basis set, basis set superposition errors should be minimal and hence no further counter-poise correction was applied. Enthalpies at 298 K have been obtained from the geometry optimization step (PBEh-3c) by using the rigid-rotor harmonic oscillator (RRHO) approximation,[S29] as implemented in ORCA.

The bond dissociation enthalpies were obtained by optimizing the individual fragments and subsequent single point computations of the relaxed fragments at PW6B95-D3(BJ)/def2-QZVPP.

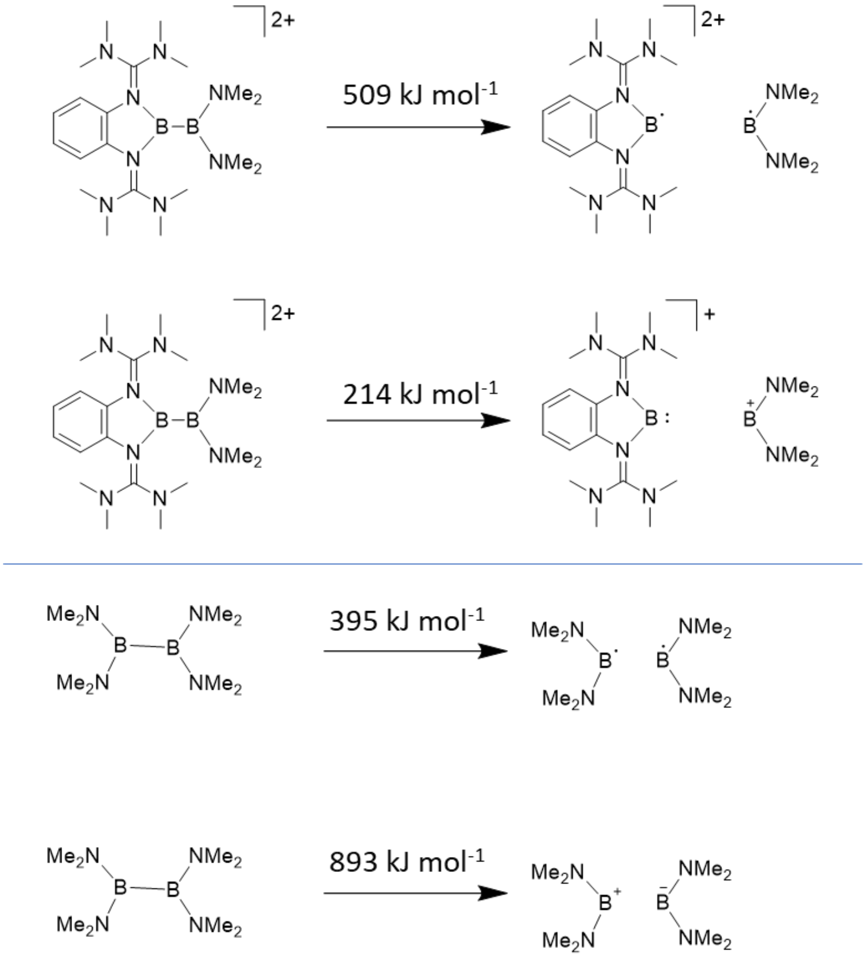

Figure S30: Computed bond dissociation enthalpies (ΔH, PW6B95-D3(BJ)/def2-QZVPP//PBEh-3c) for the homolytic and heterolytic bond cleavages, leading to the relaxed fragments.

The FIA reaction enthalpies were calculated according to the scheme proposed by Krossing,[S30] using the therein given G3 anchor points and isodesmic reactions. The solvation Gibbs free energies ΔG_{solv} were obtained from COSMO-RS[S31] implemented in the ADF program package,[S32] based on BP86-D3/TZ2P[S33] single point energy calculations for the solute-solvent interaction.

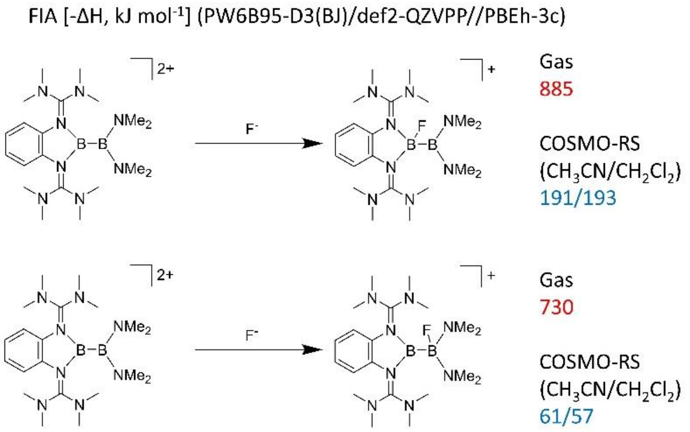

Figure S31: Computed fluoride ion affinities (-ΔH, PW6B95-D3(BJ)/def2-QZVPP//PBEh-3c) with and without solvation correction.

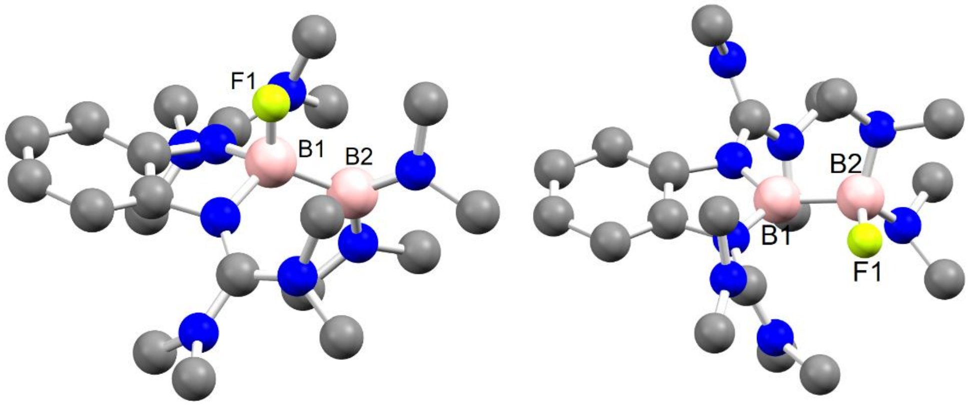

Figure S32: Illustration of the two calculated structures of **P4$_{isomer}$** after fluorid addition (left: **P4$_{isomer}$F1**; right: **P4$_{isomer}$F2**).

The ^{11}B NMR chemical shifts (Table S3) of both fluoride adducts (Figure S31 and Figure S32) of **P4$_{isomer}$** (structures optimized with PBEh-3c) were calculated using the Gauge Including Atomic Orbital method as implemented in Turbomole[S12] at the BP86/SV(P) level of theory[S15,S17] and referenced to **P4$_{isomer}$**.

Table S3: Calculated ^{11}B NMR shifts of **P4$_{isomer}$F1** and **P4$_{isomer}$F2**.

calculated ^{11}B NMR shifts		
	B(GFA) (**B1**)	B(NMe$_2$)$_2$ (**B2**)
**P4$_{isomer}$F1**	10	37
**P4$_{isomer}$F2**	46	10

NBO analysis

NBO charge analysis was performed with the NBO 7.0 program suite based on the B3LYP-D3/def2-TZVP electron density.[S34]

Table S4: Results for the NBO analysis for **P4**$_{\textbf{isomer}}$, **P4**$_{\textbf{isomer}}$ and **TDADB**.

NBO		
	$B(NMe_2)_2$ / B2	B(guanidine) / B1
NBO-charge		
**P4**$_{\textbf{Isomer}}$	+0.58	+0.84
**P2**$_{\textbf{Isomer}}$	+0.60	+0.72
	$B(NMe_2)_2$	
NBO-charge (**TDADB**)	+ 0.648	

QTAIM analysis

QTAIM analyses were performed on the B3LYP-D3/def2-TZVP electron densities, using the AIMAll software with default integration.[S35] A variety of descriptors have been developed in the context of Bader's theory of AIM, to describe and compare the nature of chemical bonds.[S36] The components considered in the QTAIM analysis are the electron density, $\rho(\mathbf{r}_{BCP})$, and the Laplacian of the density, $\nabla^2\rho(\mathbf{r}_{BCP})$. Low electron densities and positive Laplacians as found in the B-N bonds suggest closed shell (ionic) interactions whereas the opposite is found for B-B bonds, meaning shared (covalent) interactions. The total electronic energy density $H(\mathbf{r}_{BCP})$ serves as a criterion for the bond classification, with negative values for shared-type (covalent) atomic interactions (typical covalent single bonds ≈ -0.35) and positive values in purely closed-shell bonds.[S37] In addition, the ratio of $G(\mathbf{r}_{BCP})/\rho(\mathbf{r}_{BCP})$ (*Lagrangian kinetic energy per electron*) indicates the kind of interatomic interactions, with a value <1 for shared interaction and >1 for closed-shell (ionic) bonding.[S38] The ellipticity ($\varepsilon=\lambda_1/\lambda_2-1$) of a bond quantifies the anisotropy of the electron density, with deviations from a cylindrical distribution leading to values larger than zero.

Table S5: Result for the QTAIM analysis of **P4**$_{\textbf{isomer}}$ and **TDADB**.

QTAIM								
**P4**$_{\textbf{isomer}}$	$\rho(\mathbf{r}_{BCP})$	$\nabla^2\rho(\mathbf{r}_{BCP})$	$H(\mathbf{r}_{BCP})$	$G(\mathbf{r}_{BCP})$	$G(\mathbf{r}_{BCP})/\rho(\mathbf{r}_{BCP})$	$\varepsilon=\lambda_1/\lambda_2-1$	DI	
B-B	0.159	-0.410	-0.121	0.018	0.113	0.040	0.659	
B-NMe2	0.204	+0.524	-0.191	0.322	1.578	0.108	0.576	
B-N-C(NR)2Ar	0.188	+0.453	-0.173	0.286	1.521	0.066	0.545	
Me2N-C	0.390	-1.235	+0.560	0.255	0.654	0.294	1.113	
(Me2N)2C-N-B	0.328	-1.089	+0.431	0.159	0.485	0.130	0.973	
**TDADB**								
B-B	0.160	-0.387	-0.109	0.012	0.075	0.038	0.693	
B-NMe2	0.198	+0.519	-0.182	0.312	1.576	0.083	0.577	

According the QTAIM bond descriptors, the B-B and B-N bonds in **P4**$_{\textbf{isomer}}$ and **TDADB** are similar in nature, although the polarity in **P4**$_{\textbf{isomer}}$ is slightly increased, as can be seen by the more negative Laplacian, the less negative energy density $H(\mathbf{r}_{BCP})$ and the smaller $G(\mathbf{r}_{BCP})/\rho(\mathbf{r}_{BCP})$ ratio. The bcp of the B-B bond in **P4**$_{\textbf{isomer}}$ is shifted towards the $B(NMe)_2$ unit, indicating a bond polarization. However, the very negative Laplacian reveals predominant covalent character.

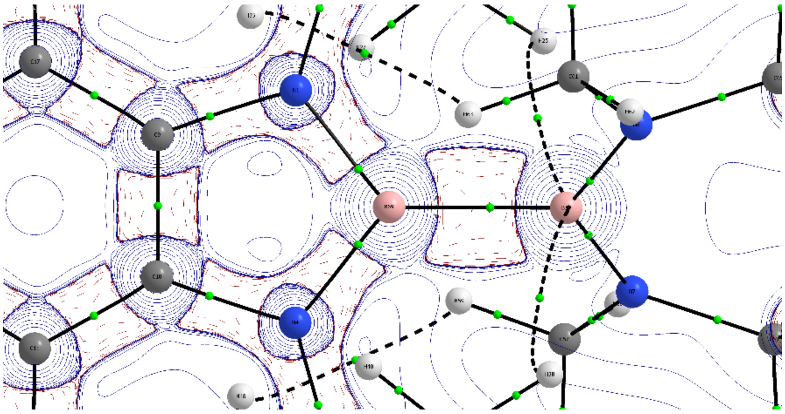

Figure S33: Contour diagram of the Laplacian of the electron density in the plane defined by N-B-B.

Energy decomposition analysis (EDA)

Energy decomposition analysis was performed with the ADF program package,[39] the BP86 functional[S40] including Grimmes Dispersion correction with Becke-Johnson damping and a triple-ζ Slater type basis set (TZ2P).[S33] The EDA scheme arbitrarily decomposes the interaction energies (ΔE_{int}) between the *prepared* monomers into contributions of Pauli repulsion (ΔE_{Pauli}), electrostatic interaction (ΔE_{elstat}), orbital interaction (ΔE_{orb}) and dispersion (ΔE_{disp}). To obtain the final association energies (D_e) between the *relaxed* fragments, the preparation energies (ΔE_{prep}) have to be added to the interaction energies ΔE_{int}. The fragments were chosen as depicted in Figure SXX. It is worth to note, that the dissociation energies found with this model are in good agreement to the PW6B95-D3/def2-QZVPP values obtained above. As can be seen from table XX, the general trends in the bond interaction contributions for **P4$_{isomer}$** and **TDADB** are similar, except for the coulomb interaction ΔE_{elstat}. It is significantly smaller in case of the heterolytic bond cleavage for **P4$_{isomer}$** due to the mutual repulsion of two cationic entities. In turn, this explains the low heterolytic bond dissociation energy for **P4$_{isomer}$** as a result of coulombic repulsion. Another feature that is favoring the heterolytic bond cleavage is the substantial energy gain by relaxation of the cationic $B(NMe_2)_2^+$ fragment (large $\boldsymbol{\Delta E_{prep}}$).

Table S6: Energies (kJ mol^{-1}) obtained by EDA (BP86-D3/TZ2P), percentages in parentheses give the contribution to the total interaction energy.

**Compound**	**P4$_{isomer}$**	**P4$_{isomer}$**	**TDADB**	**TDADB**
**fragmentation**	**homolytic**	**heterolytic**	**homolytic**	**heterolytic**
**ΔE_{int}**	-540	-429	-427	-1103
**ΔE_{Pauli}**	724	1041	844	1186
**ΔE_{elstat}**	**-677 (53%)**	**-573 (39%)**	**-737 (58%)**	**-1299 (56%)**
**ΔE_{orb}**	-502 (40%)	-812 (55%)	-493 (39%)	-949 (42%)
**ΔE_{disp}**	-85 (7%)	-85 (6%)	-41 (3%)	-41 (2%)
**$\sum\Delta E_{prep}$**	$[Ph(N{=}C(NMe_2)_2)_2B\bullet]^{2+}$ $(Me_2N)_2B\bullet$	$[Ph(N{=}C(NMe_2)_2)_2B]^+$ $(Me_2N)_2B^+$	2 x $(Me_2N)_2B\bullet$	$(Me_2N)_2B^+$ $(Me_2N)_2B^-$
	129	198	119	200
**D_e**	-411	-231	-308	-903

5. Computation of the transition state of P4 isomerisation

The transition state of the thermal isomerization of **P4** → **P4$_{isomer}$** was obtained by the nudge elastic band / ZOOM-TS feature as implemented in ORCA 4.2, at the PBEh-3c/def2-mSVP level of theory. Final single point energies were obtained at PW6B95-D3/def2-QZVPP and corrected for solvation by COSMO-RS (CH_3CN) as implemented in the SCM ADF program package.

Table S7: Computation results of the thermal isomerization **P4** → **P4$_{isomer}$**.

Isomerization P4 → P4$_{isomer}$		
**Thermodynamics**	ΔH	ΔG
gas-phase	-52.4	-51.9
COSMO-RS (CH_3CN)		-62.6
**Kinetics**	$\Delta^{‡}H$	$\Delta^{‡}G$
gas-phase	180.4	181.1
COSMO-RS (CH_3CN)		175.0

Figure S34: Representation of the transition state during the thermal isomerization of **P4** → **P4$_{isomer}$** (hydrogen atoms omitted).

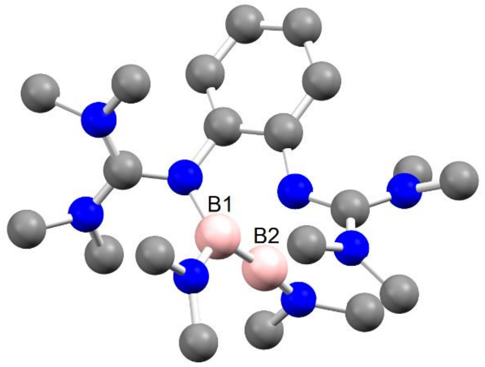

Coordinates of the transition state:

N	0.41071047654315	-0.67725172690615	-1.22354401692148
N	-0.06339255453883	-2.37490481493142	-2.77190518923952
N	1.97325378607180	-2.32288416170176	-1.68321931361743
N	0.65201307271551	-1.68308510463826	1.09704712615508
N	2.12021933086984	1.82489991444201	1.33132447818491
N	-0.87777505805573	0.77143904301632	0.38268221252291
N	-1.78725376288544	2.70056124757539	1.40977480946915
N	-1.71844765216257	0.67738636719586	2.52481379075774
C	-1.14772091855838	1.06971165486646	-0.96116905016430
C	-2.12450347246579	1.91064164991191	-1.48678817052303
H	-2.84510247857441	2.39878205358598	-0.84718458783412
C	-2.20600046244416	2.10900795773150	-2.85715453485526
H	-2.96490800370955	2.76933532606255	-3.25307620477414
C	-1.31263084655473	1.48517530379002	-3.71329252752183
H	-1.34373405146894	1.68493839358151	-4.77509432129286
C	-0.39276442611931	0.57898024458649	-3.20836321177353
H	0.27915264146166	0.06568953756638	-3.88440051509322
C	-0.36159472253069	0.32799251779630	-1.84592016473508
C	0.76574761695747	-1.79951680023833	-1.89969363318881
C	0.40907070974163	-3.05076438402268	-3.97366972407781
H	0.27384930526715	-4.13075646059492	-3.91605280817693

H	-0.16662930161838	-2.68419363781508	-4.82393090260301
H	1.45599005160011	-2.83109939672139	-4.16210185239164
C	-1.51124823673486	-2.27973396424229	-2.67237002015996
H	-1.93358542635861	-3.27952182696579	-2.77493891766220
H	-1.82100116359137	-1.87398774507536	-1.71432733174357
H	-1.92031722753222	-1.64697455988549	-3.46035260180277
C	2.24061686105279	-3.75237080760213	-1.75262707553682
H	1.31667614140805	-4.32243679827753	-1.77638114219541
H	2.84420915551101	-4.01354766687363	-2.62199221884942
H	2.79143812237058	-4.04613803462632	-0.85887824860344
C	3.09581034421845	-1.51149160183690	-1.25523076083763
H	3.99281909454702	-1.85080850284462	-1.77280247977606
H	2.93752331219674	-0.46851728306266	-1.51726689438963
H	3.26933803094886	-1.60344328771292	-0.18078601219307
C	-0.10283101401597	-2.89227091335173	0.84755049100528
H	-0.67680141558289	-3.18544505147903	1.73089513105324
H	-0.81861144573251	-2.76726243754717	0.03821468306836
H	0.54782509319346	-3.73779439064515	0.60541503508793
C	1.42078777592279	-1.77989043346296	2.31884409876138
H	2.09622297943445	-2.63958813176201	2.29352207812744
H	2.03332650965097	-0.89480160602209	2.48631466798573
H	0.78107902365034	-1.90747137421276	3.19805609086047
C	3.57079756221226	1.74770292992768	1.08354956495448
H	3.82079987441695	0.87942846035825	0.47705546187631
H	3.89745600932121	2.64146941767686	0.55400111757324
H	4.10158698394190	1.67335377142424	2.03165424646167
C	1.72447804509472	2.93705595924790	2.18574474202115
H	0.68125271466091	2.85911979951337	2.47162219137895
H	2.32857709648846	2.92020668624876	3.09231614941485
H	1.88992004257251	3.88496989460005	1.67536071712298
C	-1.46552076515276	1.38972690112399	1.41600788117407
C	-1.07196583238579	3.70772148374040	0.64747033110842
H	-1.70592008817085	4.15336350220164	-0.11907669503090
H	-0.75529695033070	4.50794067802170	1.31878250306689
H	-0.19699795002179	3.29823756699344	0.15212811481756
C	-2.96710270199698	3.21063038710288	2.09896704997082
H	-3.63104807497170	2.40282982639685	2.39219191137934
H	-2.71030085256896	3.80057438543682	2.97943734355415
H	-3.51807950674151	3.85646496189787	1.41461113083963
C	-2.13672894952295	-0.71140444459141	2.47259512458068
H	-3.10605918703351	-0.81206390207054	2.96436862806117
H	-2.23398492838775	-1.04544119582453	1.44578465423060
H	-1.42825291133116	-1.35370452903686	2.99362050166568
C	-1.68797664264275	1.24314869908253	3.86313298057959
H	-2.68586392255454	1.36831850108821	4.28558682037295
H	-1.13531427627246	0.56502572893351	4.51412501066227
H	-1.18179247154515	2.20460346485636	3.87604492203672
B	0.60127652720716	-0.52833811428979	0.27434926404968
B	1.39123836161672	0.83247087328966	0.81862707157140

6. Kinetic data for the isomerisation process of P4

The isomerisation process of **P4**(OTf)$_2$ was followed by ^{1}H NMR spectroscopy at different temperatures (T = 298.2, 308.4, 318.2 K). Therefore, solutions of **P4**(OTf)$_2$ (10.0 mg, 14.3 µmol) in CD_3CN (0.5 ml) were prepared and ^{1}H NMR spectra were recorded with increasing reaction time. The error of the measurements was calculated by the gaussian error propagation (ΔT = 0.5 K; integration error = 5%). The temperature was calibrated by the method from Berger et. al. [S41]

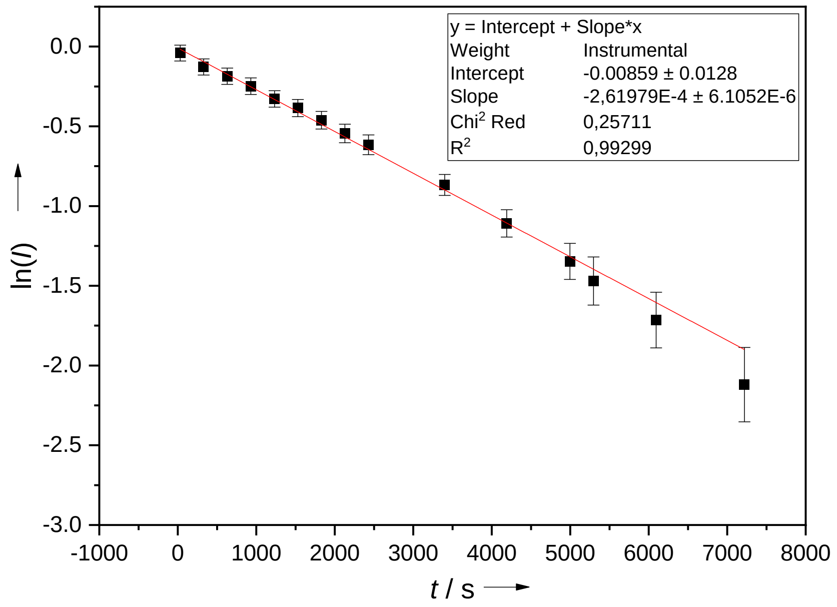

Figure S35: First-order plot of ln(I-I$_0$) vs. time, based on the relative signal intensities from ^{1}H NMR integration of **P4**(OTf)$_2$ and **P4**$_{\mathbf{isomer}}$(OTf)$_2$ at different times with T = 298.2 K.

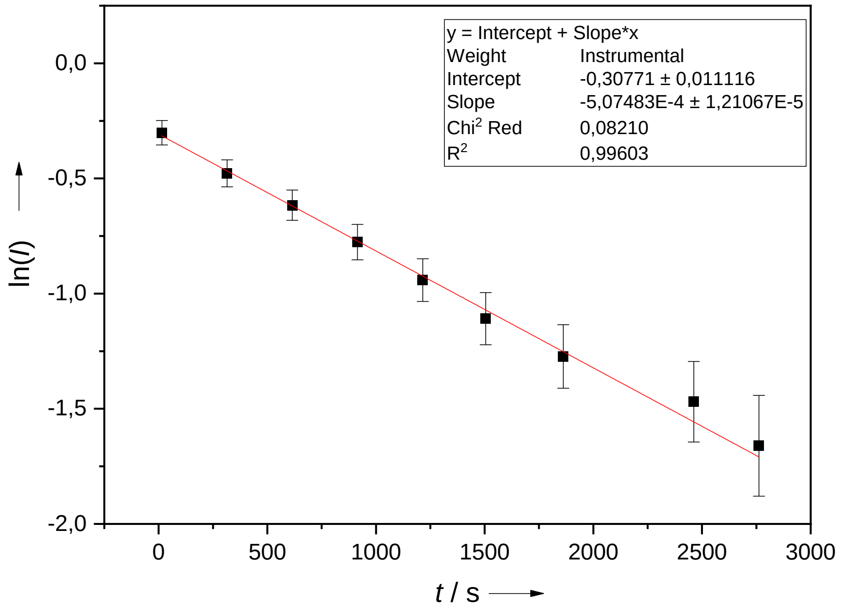

Figure S36: First-order plot of ln(I-I$_0$) vs. time, based on the relative signal intensities from ^{1}H NMR integration of **P4**(OTf)$_2$ and **P4**$_{\mathbf{isomer}}$(OTf)$_2$ at different times with T = 308.4 K.

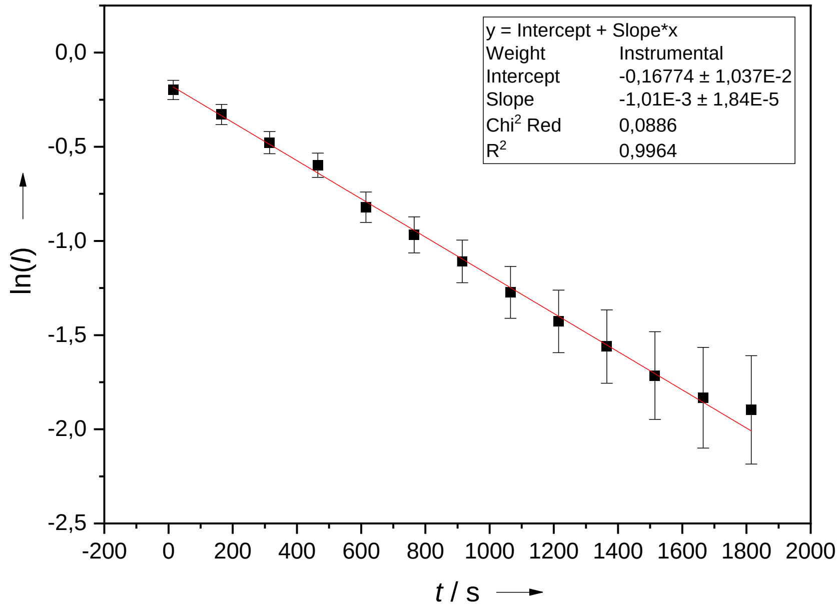

Figure S37: First-order plot of ln(I-I$_0$) vs. time, based on the relative signal intensities from ^{1}H NMR integration of **P4**(OTf)$_2$ and **P4$_{isomer}$**(OTf)$_2$ at different times with T = 318.2 K.

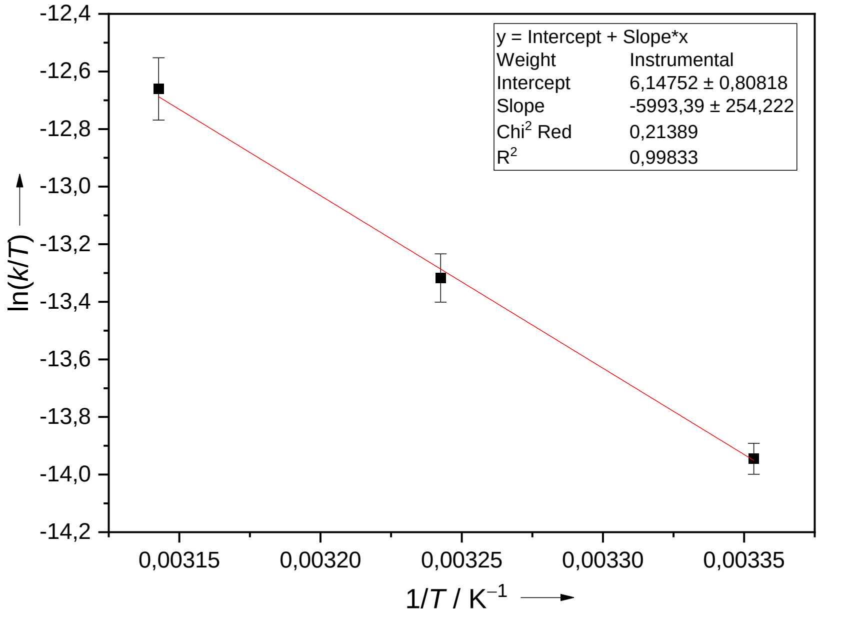

Figure S38: Eyring plot for the isomerisation of **P4**(OTf)$_2$ to **P4$_{isomer}$**(OTf)$_2$, based on the determined rate constants (k) at T = 298.2; 308.4 and 318.2 K.

$\Delta H^{\ddagger} = 49.86 \pm 2.04$ kJ mol^{-1}

Eyring-equation:

$$k = x\frac{k_B T}{h} e^{\frac{-\Delta G^{\ddagger}}{RT}}$$

for $x = 1$:

$$\Delta G^{\ddagger} = -\ln\left(\frac{kh}{k_B T}\right) RT$$

$$\ln\left(\frac{k}{T}\right) = ln\left(\frac{k_B}{h}\right) - \left(\frac{\Delta H^{\ddagger}}{R}\right) \cdot \frac{1}{T} + \left(\frac{\Delta S^{\ddagger}}{R}\right)$$

T / K	$\Delta G^{\ddagger}$ */ kJ/mol*
298.2	93.5 ± 0.2
308.4	95.1 ± 0.2
318.2	96.4 ± 0.2

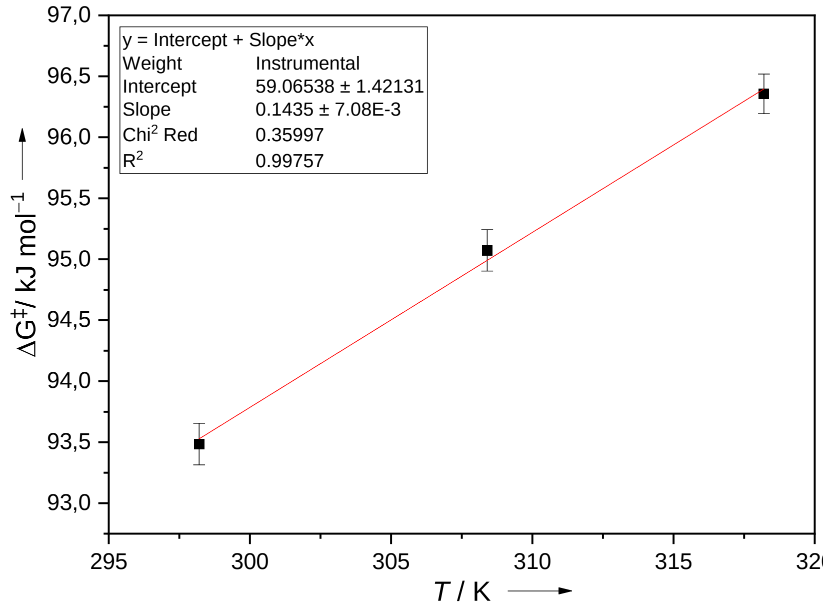

Figure S39: Plot of $\Delta G^{\ddagger}$ vs. T, based on the results from the Eyring-equation.

$\Delta S^{\ddagger} = -143.5 \pm 7.1$ J mol^{-1} K^{-1}

The isomerisation process of **P4**($GaCl_4$)$_2$ was followed by ^{1}H NMR spectroscopy at different temperatures (T = 293.2, 313.6, 324.4 K). Therefore, solutions of **P4**($GaCl_4$)$_2$ in CD_3CN (with different concentrations) were prepared and ^{1}H NMR spectra were recorded with increasing reaction time. The error of the measurements was calculated by the gaussian error propagation (ΔT = 0.5 K; integration error = 5%). The temperature was calibrated by the method from Berger et al.[S42]

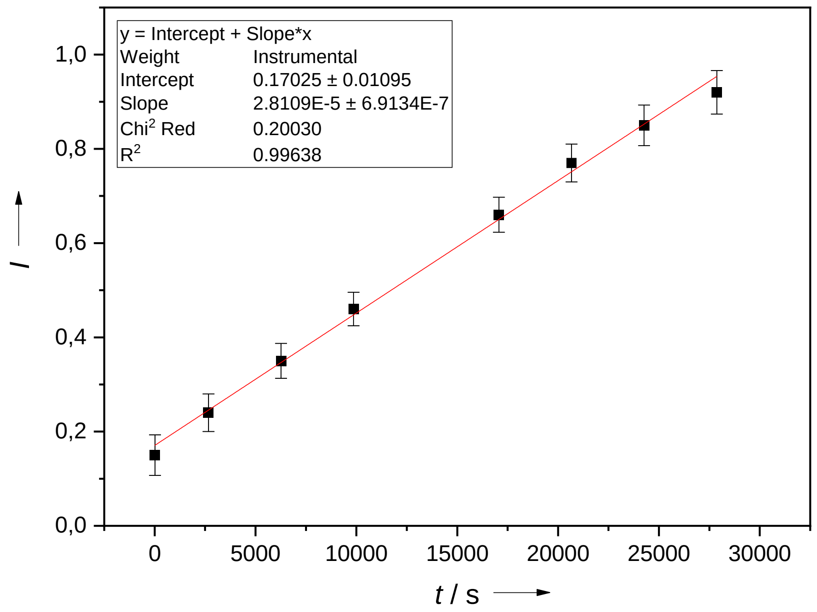

Figure S40: Zero-order plot of *I* vs. time, based on the relative signal intensities from ^{1}H NMR integration of **P4**($GaCl_4$)$_2$ and **P4**$_{\textbf{isomer}}$($GaCl_4$)$_2$ at different times with T = 293.2 K, 23.9 mmol l^{-1}.

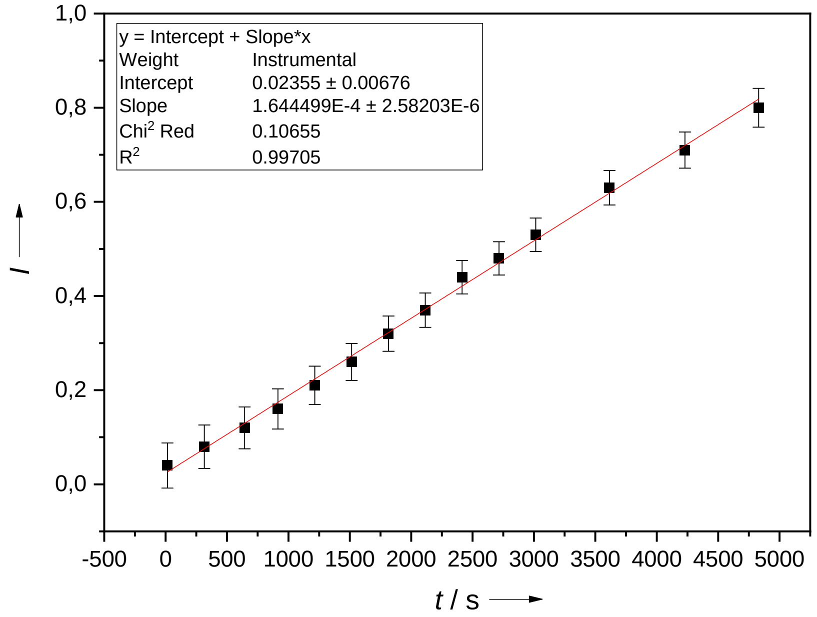

Figure S41: Zero-order plot of *I* vs. time, based on the relative signal intensities from ^{1}H NMR integration of **P4**($GaCl_4$)$_2$ and **P4**$_{\textbf{isomer}}$($GaCl_4$)$_2$ at different times with T = 313.6 K, 23.9 mmol l^{-1}.

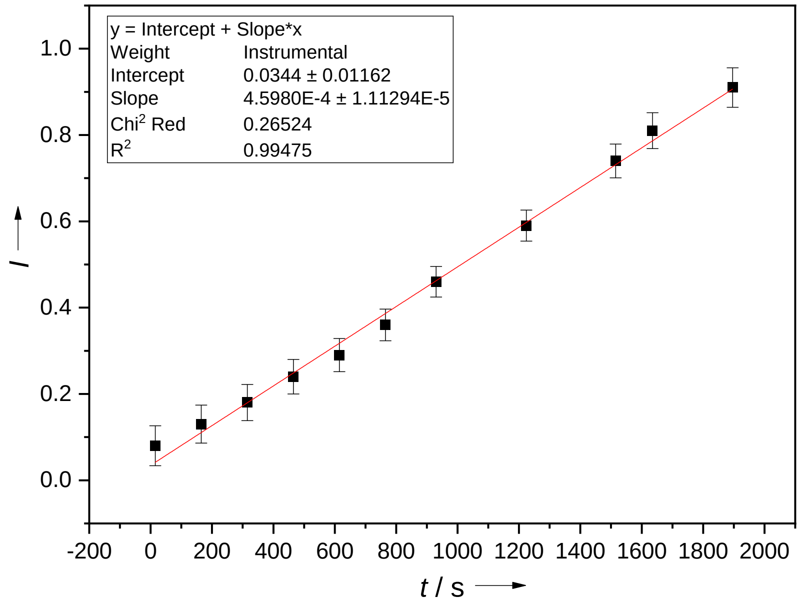

Figure S42: Zero-order plot of *I* vs. time, based on the relative signal intensities from ^{1}H NMR integration of **P4**$(GaCl_4)_2$ and $\mathbf{P4}_{\mathbf{isomer}}(GaCl_4)_2$ at different times with T = 324.4 K, 23.9 mmol l^{-1}.

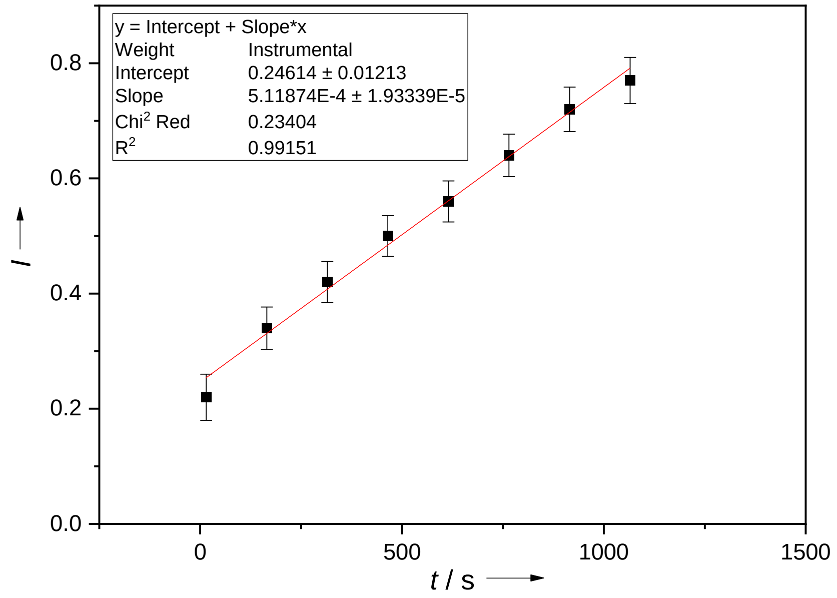

Figure S43: Zero-order plot of *I* vs. time, based on the relative signal intensities from ^{1}H NMR integration of **P4**$(GaCl_4)_2$ and $\mathbf{P4}_{\mathbf{isomer}}(GaCl_4)_2$ at different times with T = 324.4 K, 7.16 mmol l^{-1}.

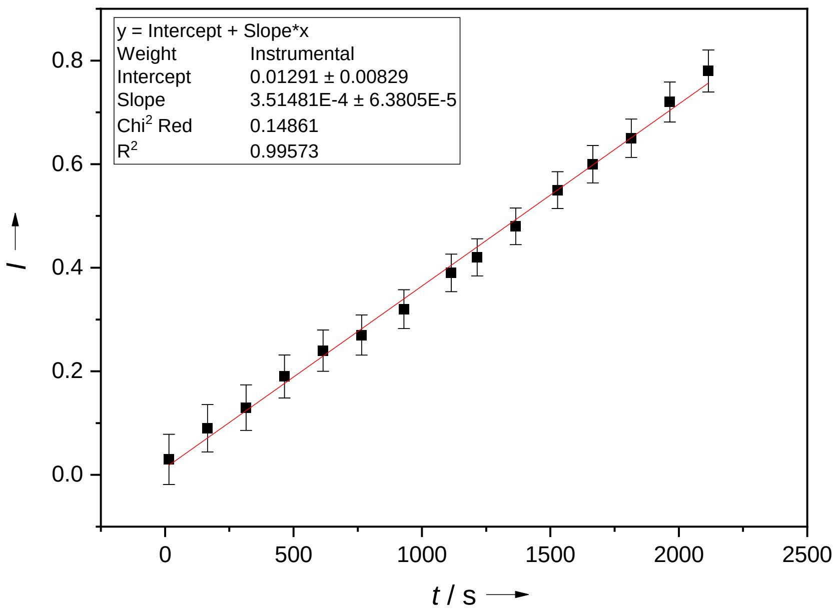

Figure S44: Zero-order plot of *I* vs. time, based on the relative signal intensities from ^{1}H NMR integration of **P4**($GaCl_4$)$_2$ and **P4**$_{\mathbf{isomer}}$($GaCl_4$)$_2$ at different times with T = 324.4 K, 47.8 mmol l^{-1}.

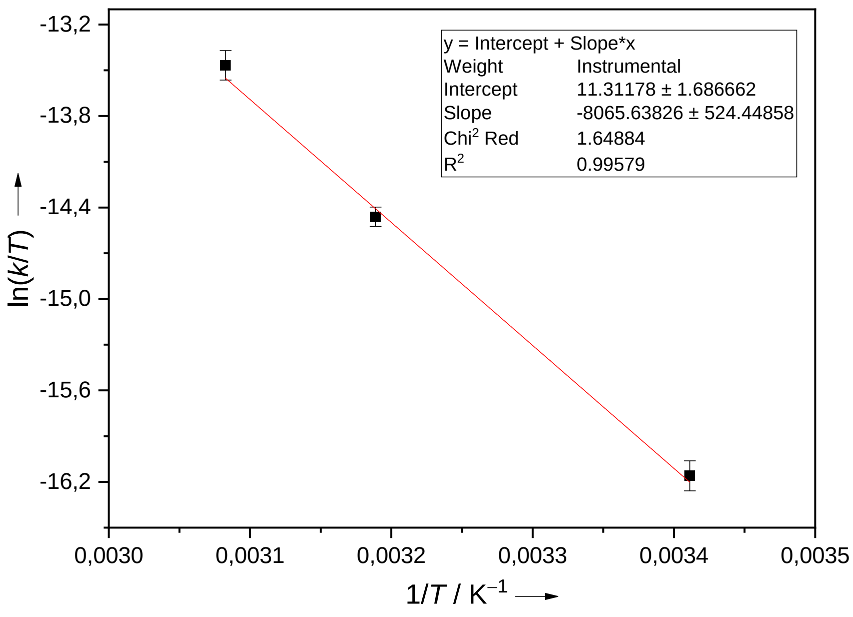

Figure S45: Eyring plot for the isomerisation of **P4**($GaCl_4$)$_2$ to **P4**$_{\mathbf{isomer}}$($GaCl_4$)$_2$, based on the determined rate constants (k) at T = 293.2; 313.6 and 324.4 K, for 23.9 mmol l^{-1}.

$\Delta H^{\ddagger} = 67.09 \pm 4.36$ kJ mol^{-1}

Eyring-equation:

$$k = x\frac{k_B T}{h}e^{\frac{-\Delta G^{\ddagger}}{RT}}$$

for x = 1:

$$\Delta G^{\ddagger} = -\ln\left(\frac{kh}{k_B T}\right)RT$$

$$\ln\left(\frac{k}{T}\right) = ln\left(\frac{k_B}{h}\right) - \left(\frac{\Delta H^{\ddagger}}{R}\right)\cdot\frac{1}{T} + \left(\frac{\Delta S^{\ddagger}}{R}\right)$$

T / K	$\Delta G^{\ddagger}$ */ kJ/mol*
293.2	97.3 ± 0.2
313.6	99.7 ± 0.2
324.4	100.4 ± 0.2

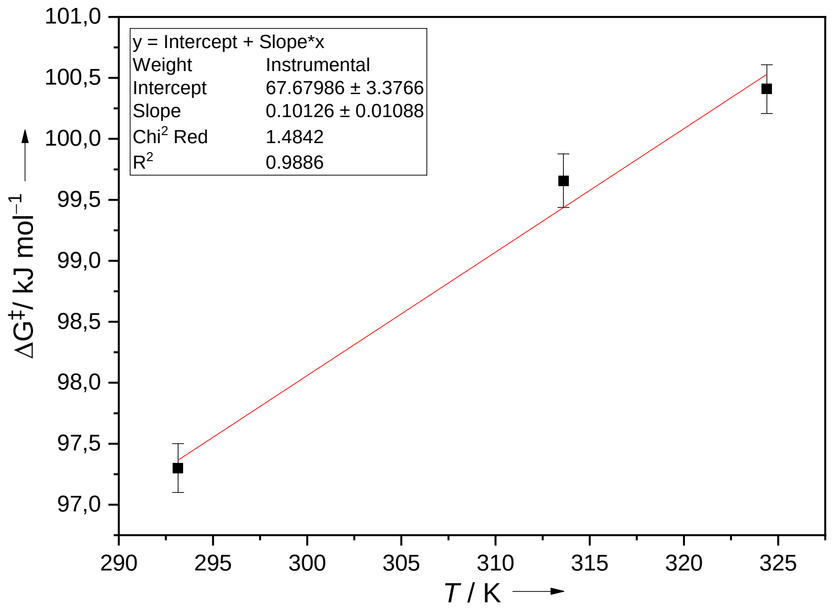

Figure S46: Plot of $\Delta G^{\ddagger}$ vs. T, based on the results from the Eyring-equation, for 23.9 mmol l^{-1}.

$\Delta S^{\ddagger} = -101.3 \pm 10.9$ J mol^{-1} K^{-1}

Reaction zero-order kinetic on [P4]($GaCl_4$)$_2$

Obviously, the fit of the zero-order kinetic shows only small deviations from the experiment (see Figures S39-43). However, the rate decrease upon increase of the starting concentration (Table S8) of [**P4**]($GaCl_4$)$_2$ argues for a more complex mechanism. We suggest the catalysis by Cl^- ions generated in small quantities in a rate-determing preequilibrium reaction from $GaCl_4^-$ (Figure S47). A higher concentration of [**P4**]($GaCl_4$)$_2$ is related to a higher concentration of $GaCl_4^-$. We suggest a shift of the equilibrium to the side of $GaCl_4^-$ since the chance to build an ion-pair is increasing with increasing concentrations. Thus, less Cl^- ions per **P4** are available for the catalytic conversion and the rate is decreasing.

$$GaCl_4^- \rightleftharpoons GaCl_3 + Cl^-$$

Figure S47: Formation of Cl^- ions.

Table S8: Reaction rate of [**P4**]($GaCl_4$)$_2$ with varying starting concentrations.

c [**P4**]($GaCl_4$)$_2$ / mmol l^{-1}	*k* (T = 324.4 K) / s^{-1}
7.16	$5.12\times10^{-4} \pm 1.93\times10^{-5}$
23.9	$4.60\times10^{-4} \pm 1.11\times10^{-5}$
47.8	$3.51\times10^{-4} \pm 6.38\times10^{-5}$

7. NMR Spectra:

^{1}H NMR spectrum (400 MHz, CD_3CN) for **L2**:

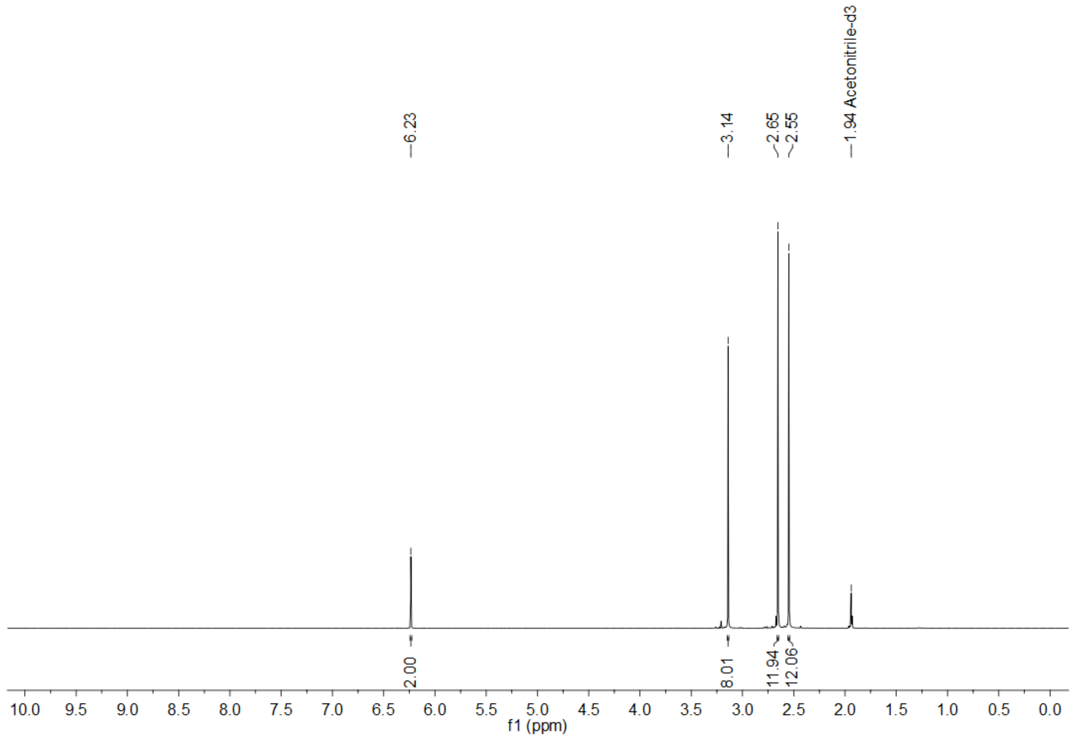

^{13}C NMR spectrum (150 MHz, CD_3CN) for **L2**:

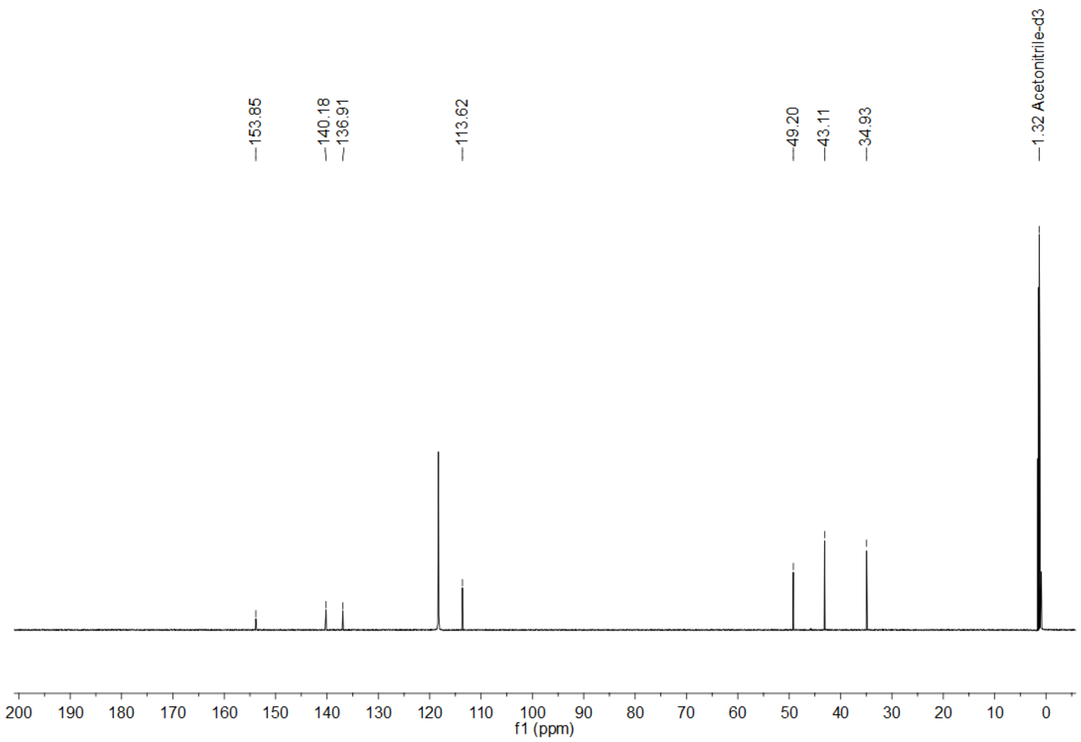

^{1}H NMR spectrum (400 MHz, CD_3CN) for **P1**$(OTf)_4$:

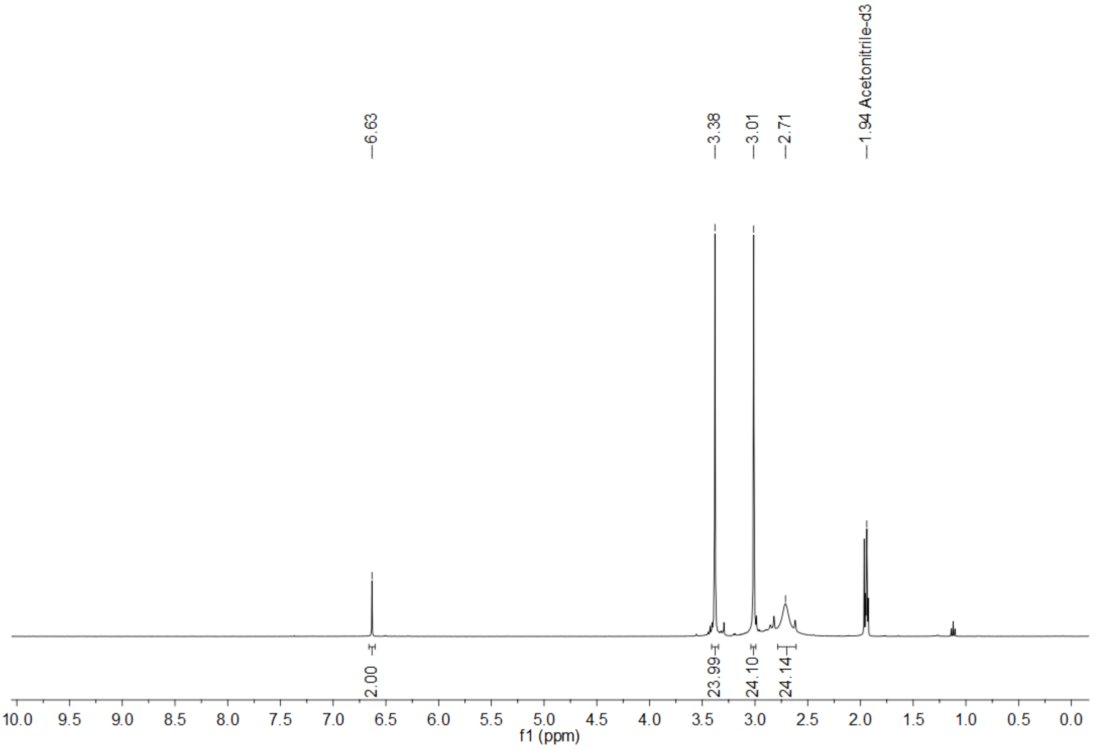

^{13}C NMR spectrum (100 MHz, CD_3CN) for **P1**$(OTf)_4$:

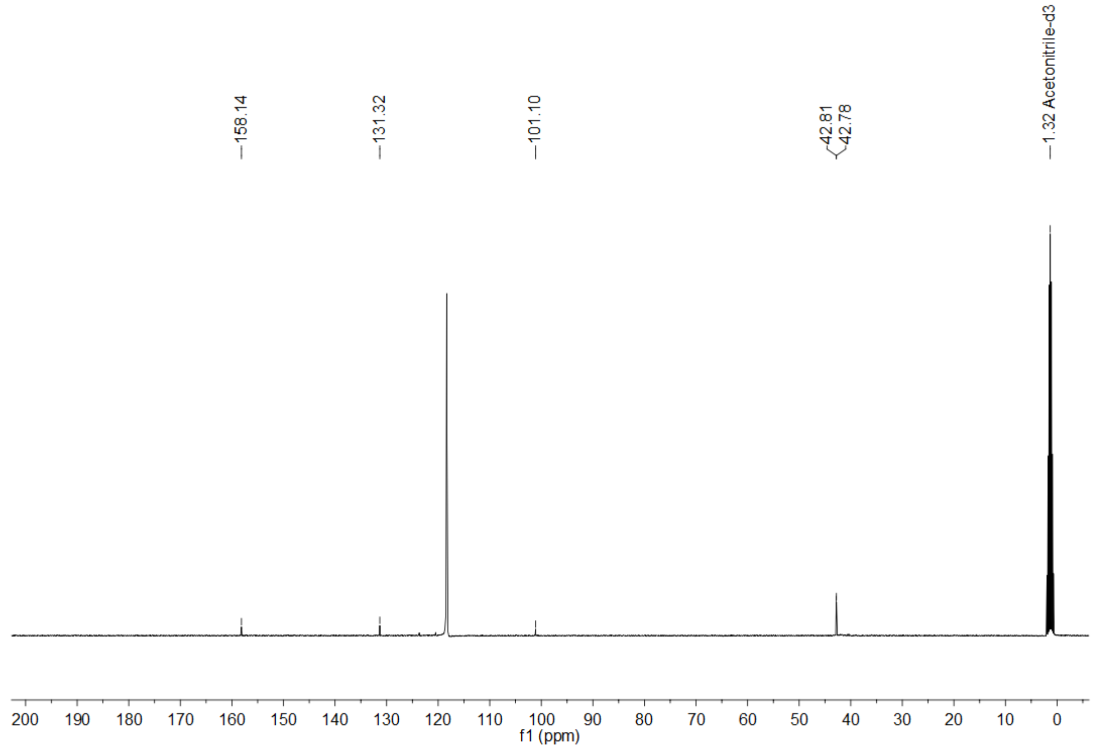

^{11}B NMR spectrum (128 MHz, CD_3CN) for **P1**$(OTf)_4$:

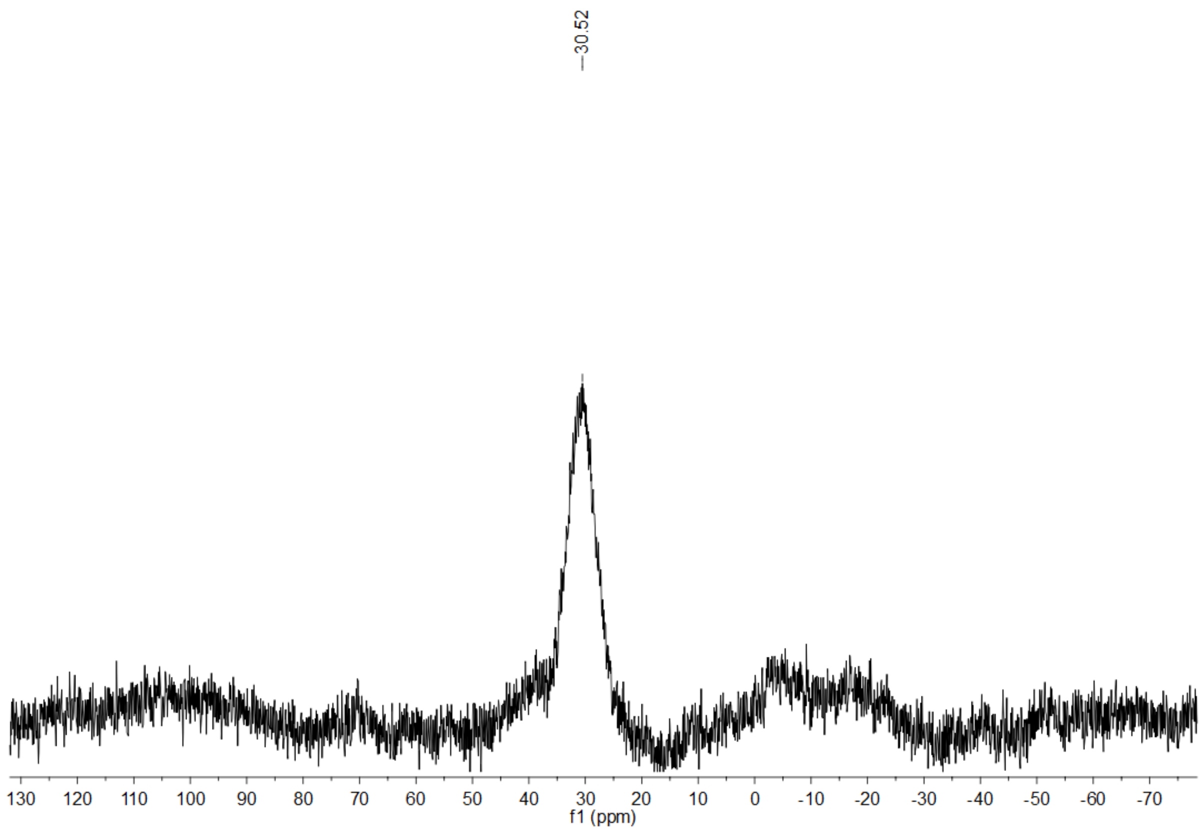

^{1}H NMR spectrum (400 MHz, CD_3CN) for **P3**[OTf]$_2$:

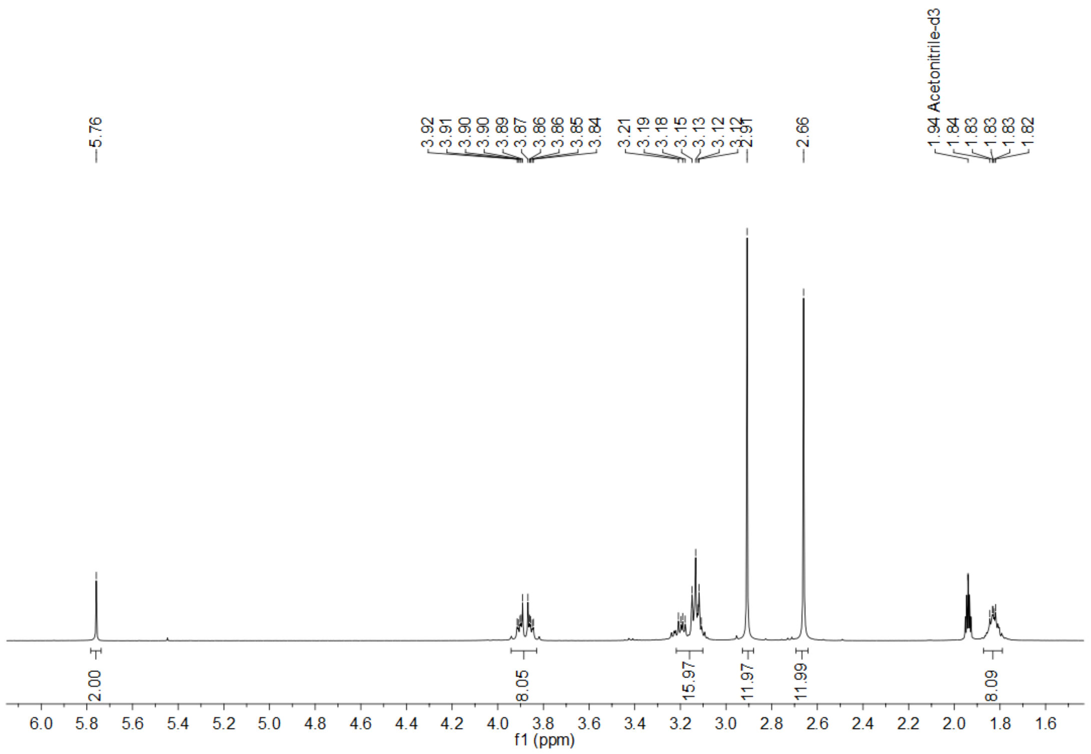

^{13}C NMR spectrum (100 MHz, CD_3CN) for **P3**[OTf]$_2$:

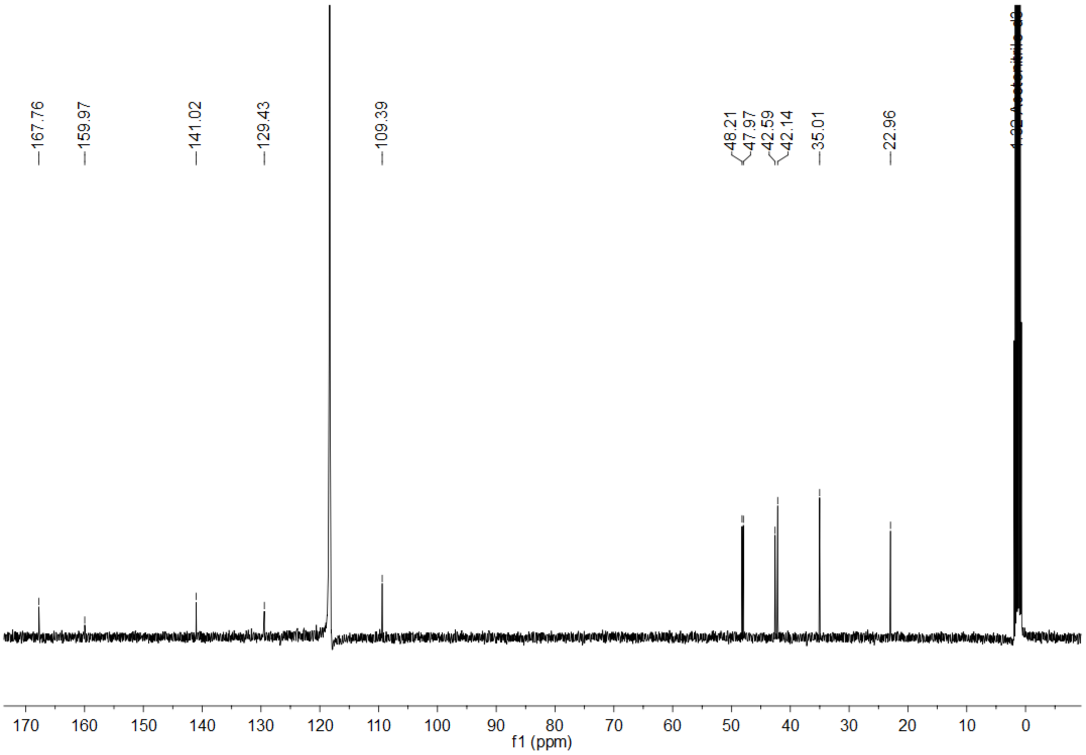

^{11}B NMR spectrum (128 MHz, CD_3CN) for **P3**[OTf]$_2$:

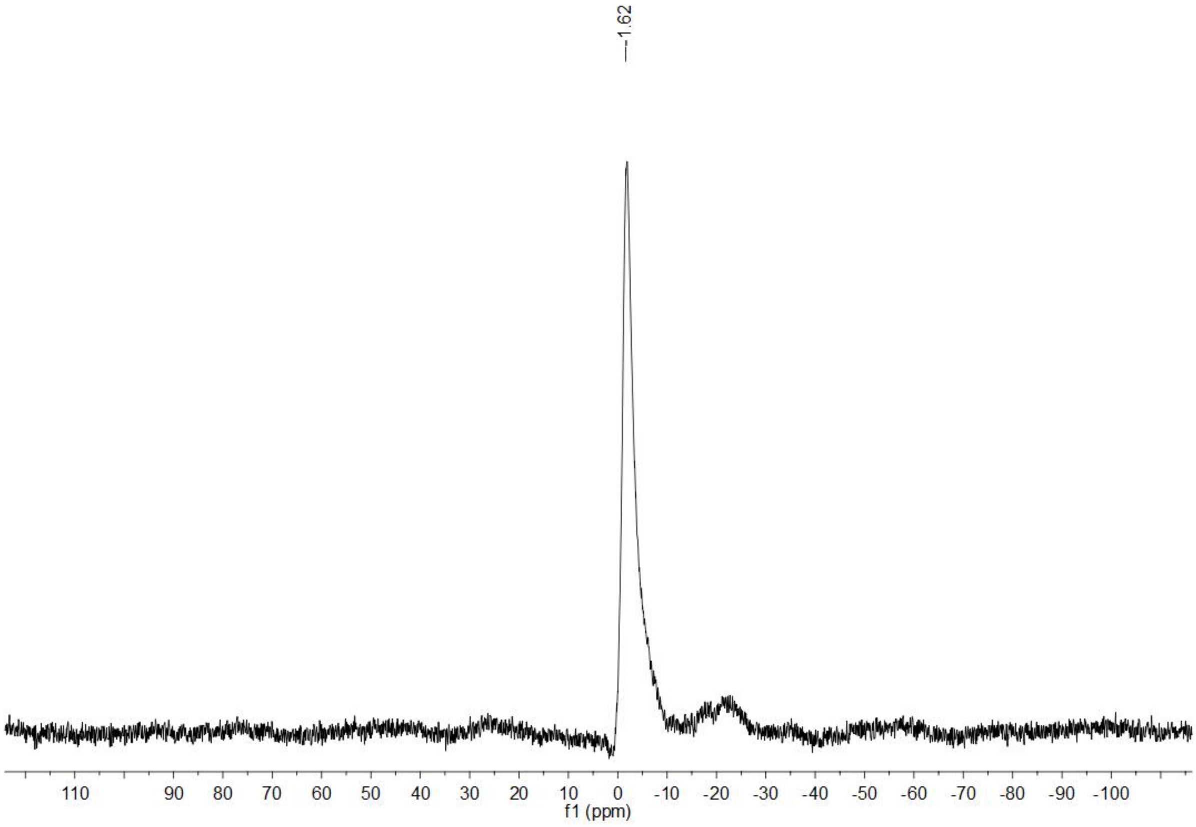

^{19}F NMR spectrum (376 MHz, CD_3CN) for **P3**[OTf]$_2$:

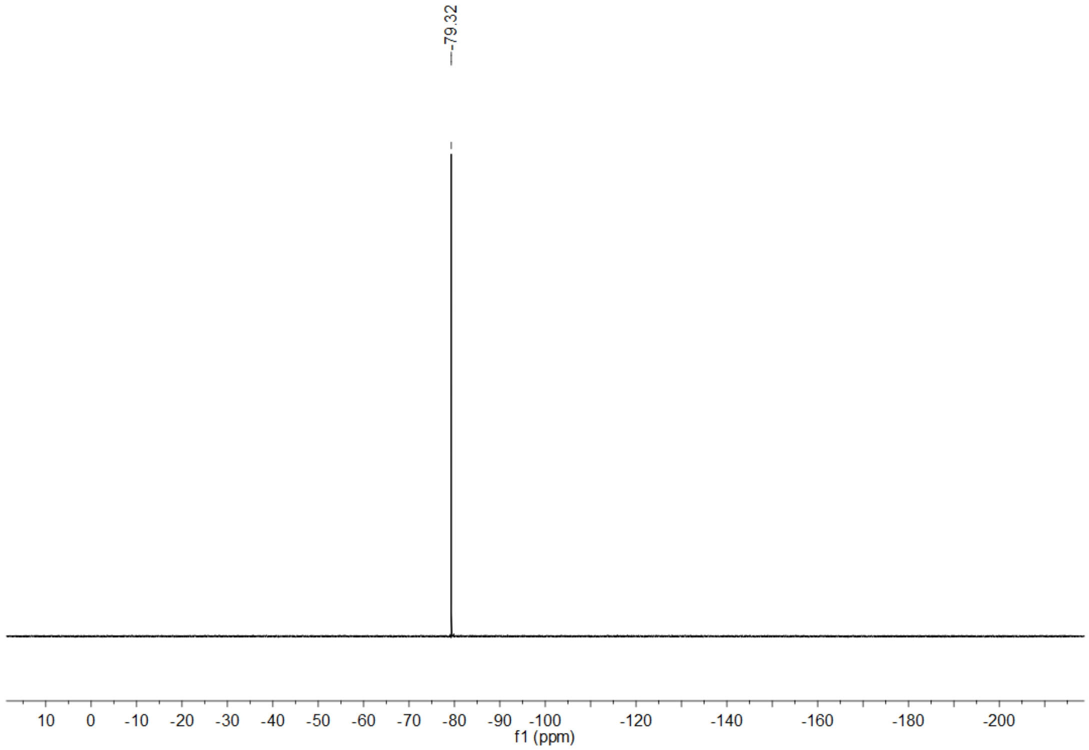

^{1}H NMR spectrum (400 MHz, CD_3CN) for **P2$_{isomer}$**($GaCl_4$)$_2$:

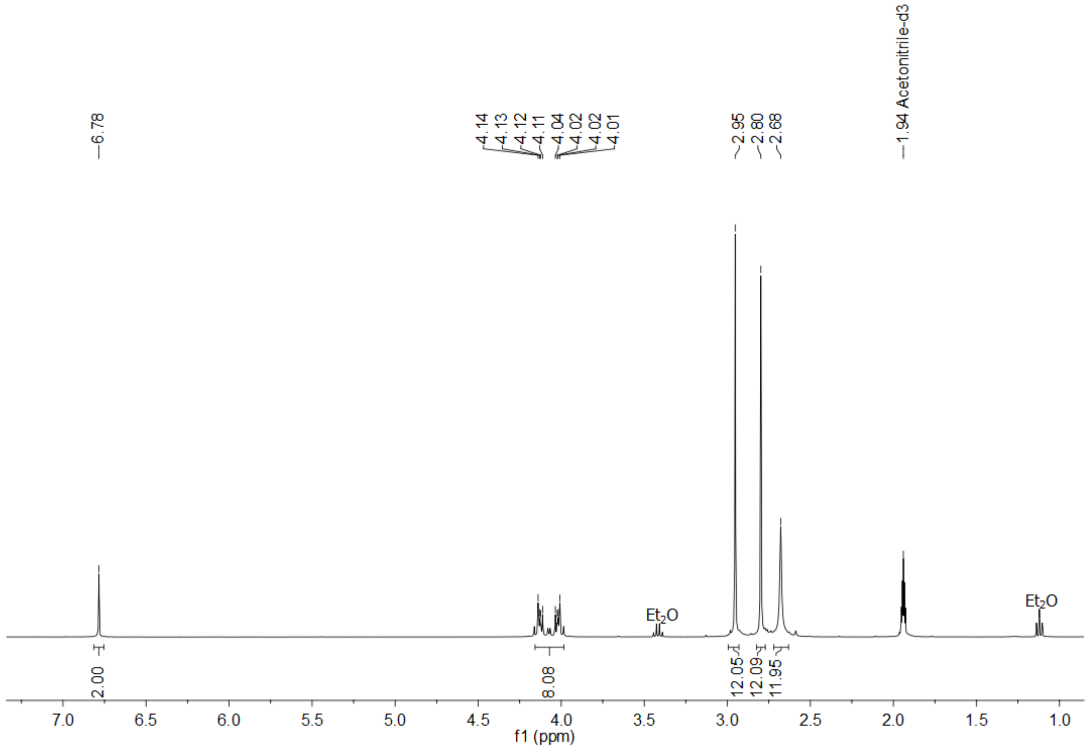

^{13}C NMR spectrum (100 MHz, CD_3CN) for **P2$_{isomer}$**$(GaCl_4)_2$:

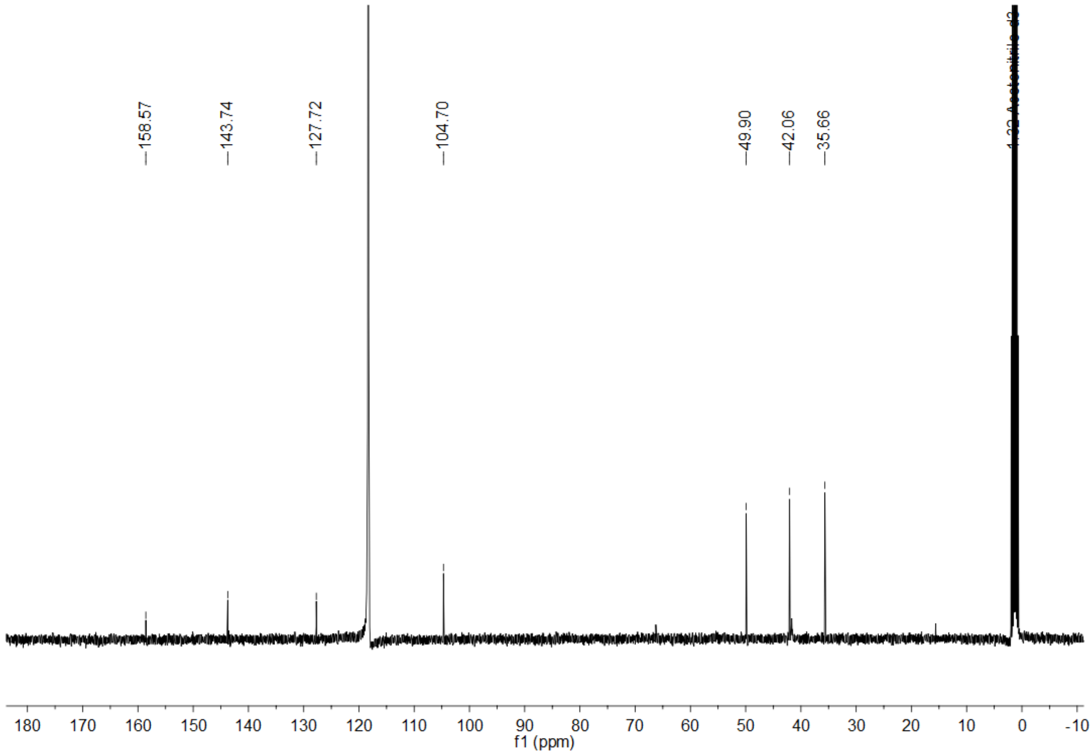

^{11}B NMR spectrum (128 MHz, CD_3CN) for **P2$_{isomer}$**$(GaCl_4)_2$:

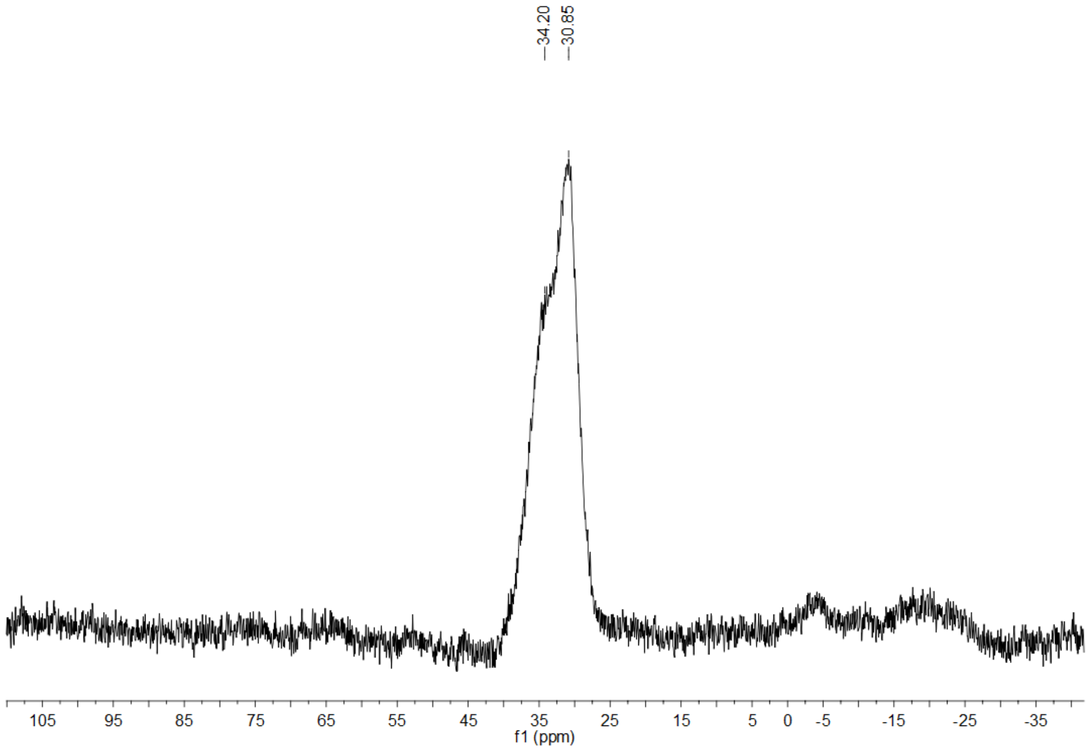

$^{11}B\{^{1}H\}$ NMR spectrum (128 MHz, CD_3CN) for **P2**$_{\mathbf{isomer}}$($GaCl_4$)$_2$:

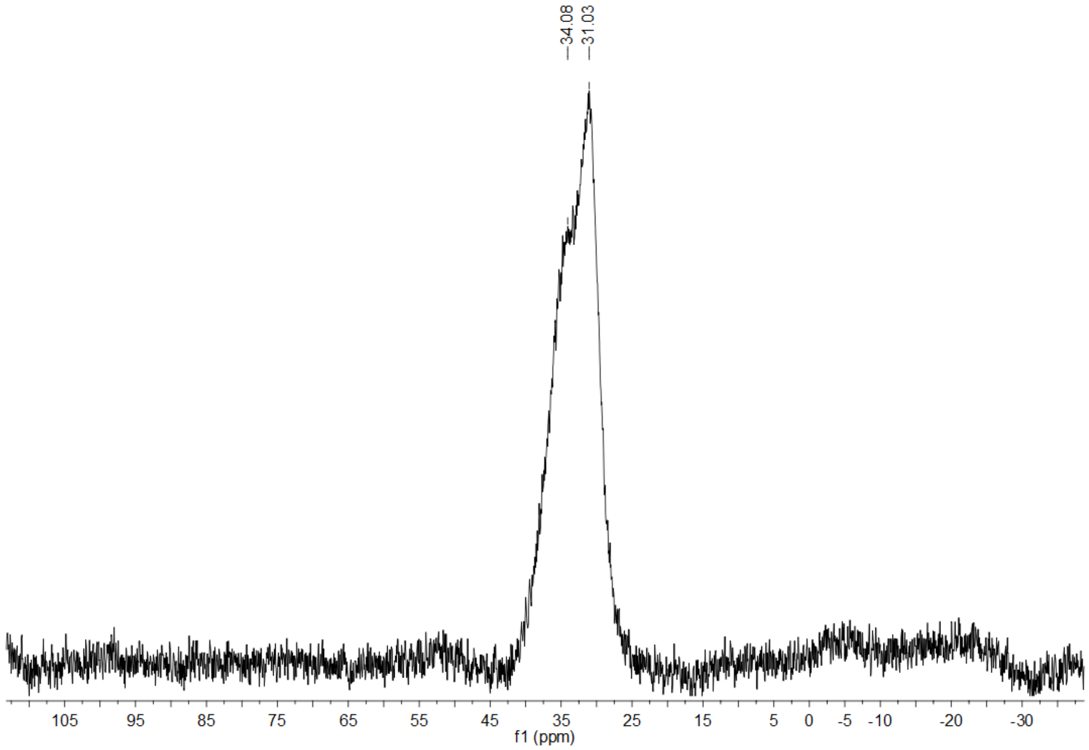

^{1}H NMR spectrum (400 MHz, CD_3CN) for **P2**$_{\mathbf{isomer}}$($AlCl_4$)$_2$:

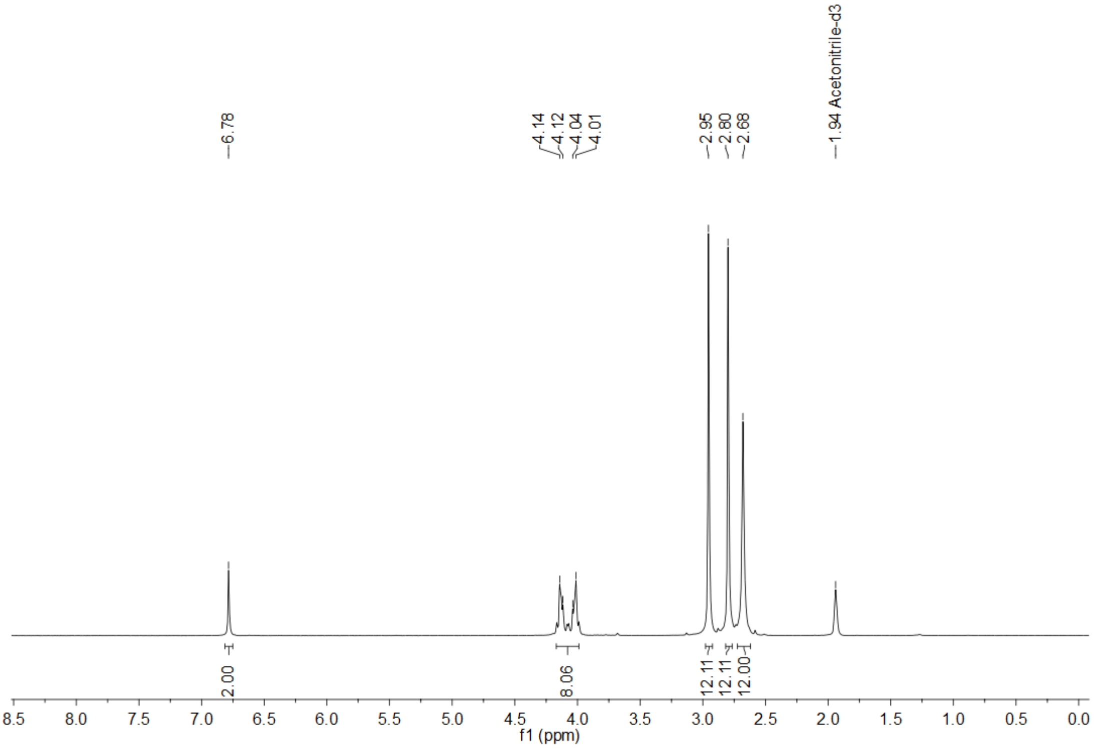

^{13}C NMR spectrum (100 MHz, CD_3CN) for **P2$_{isomer}$**$(AlCl_4)_2$:

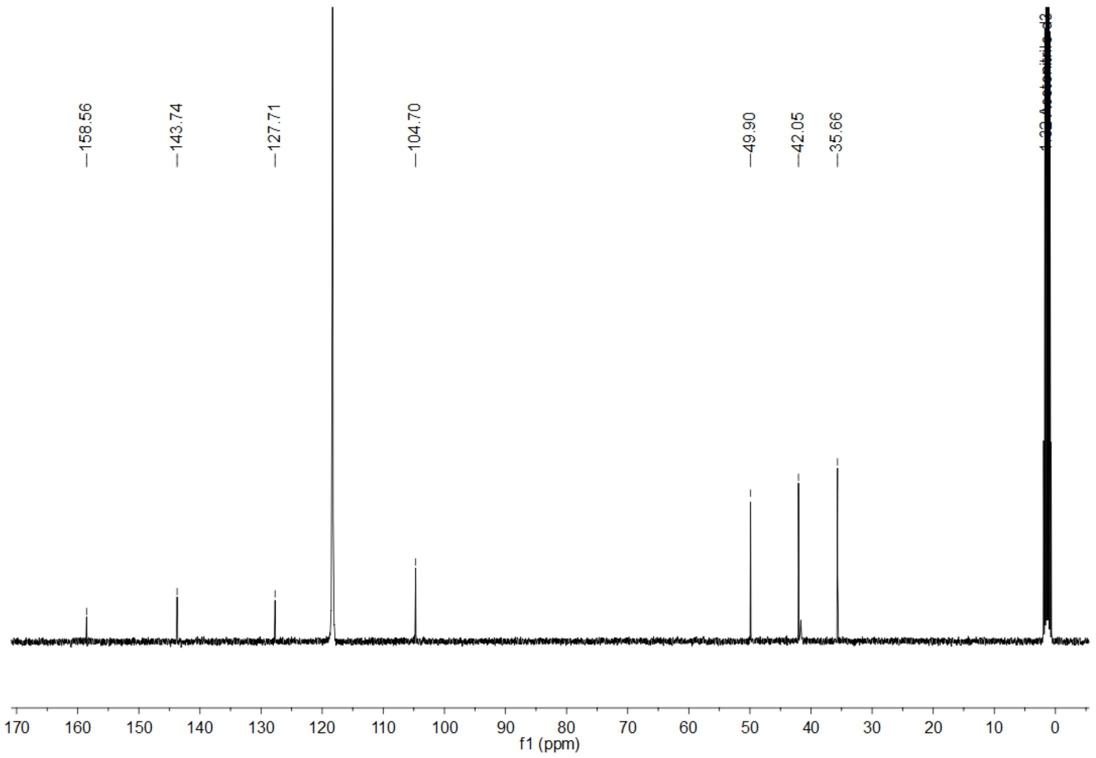

^{11}B NMR spectrum (128 MHz, CD_3CN) for **P2$_{isomer}$**$(AlCl_4)_2$:

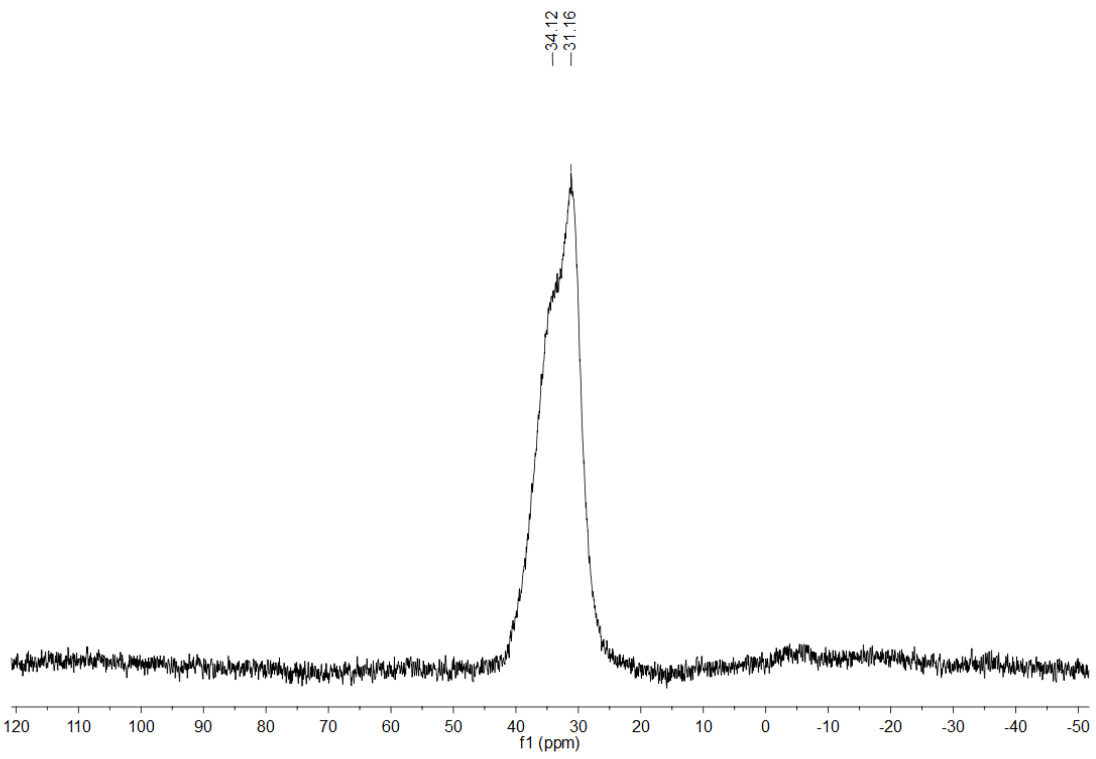

$^{11}B\{^{1}H\}$ NMR spectrum (128 MHz, CD_3CN) for **P2$_{isomer}$**$(AlCl_4)_2$:

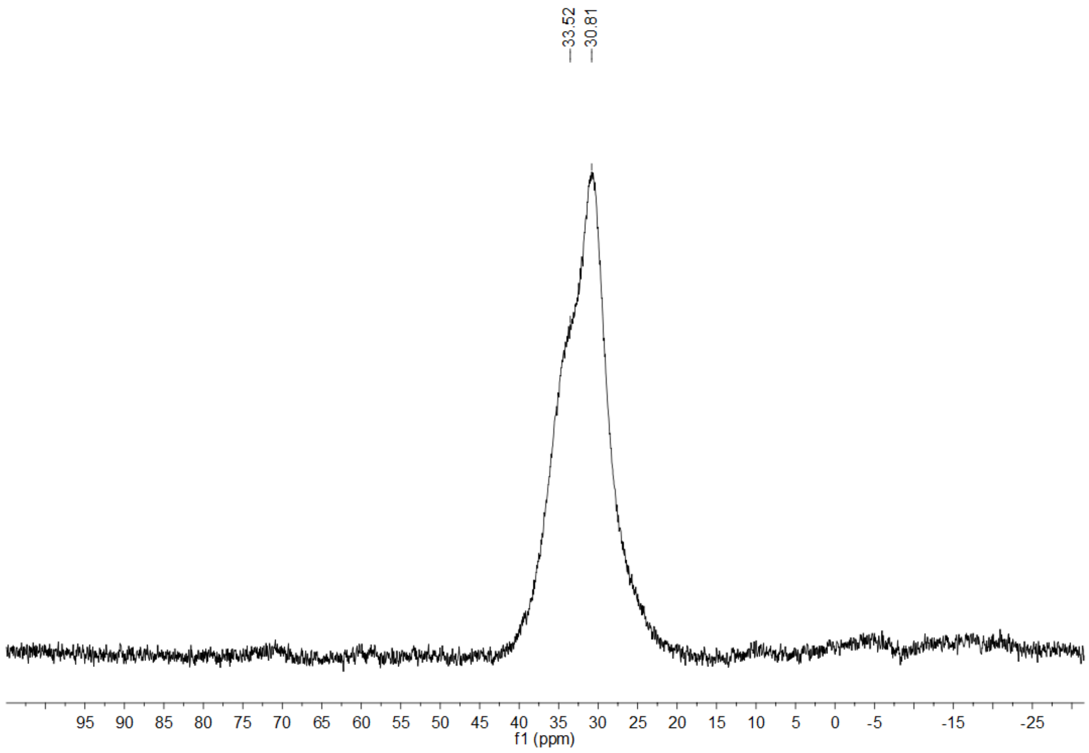

^{1}H NMR spectrum (400 MHz, CD_3CN) for **P4$_{isomer}$**$(GaCl_4)_2$:

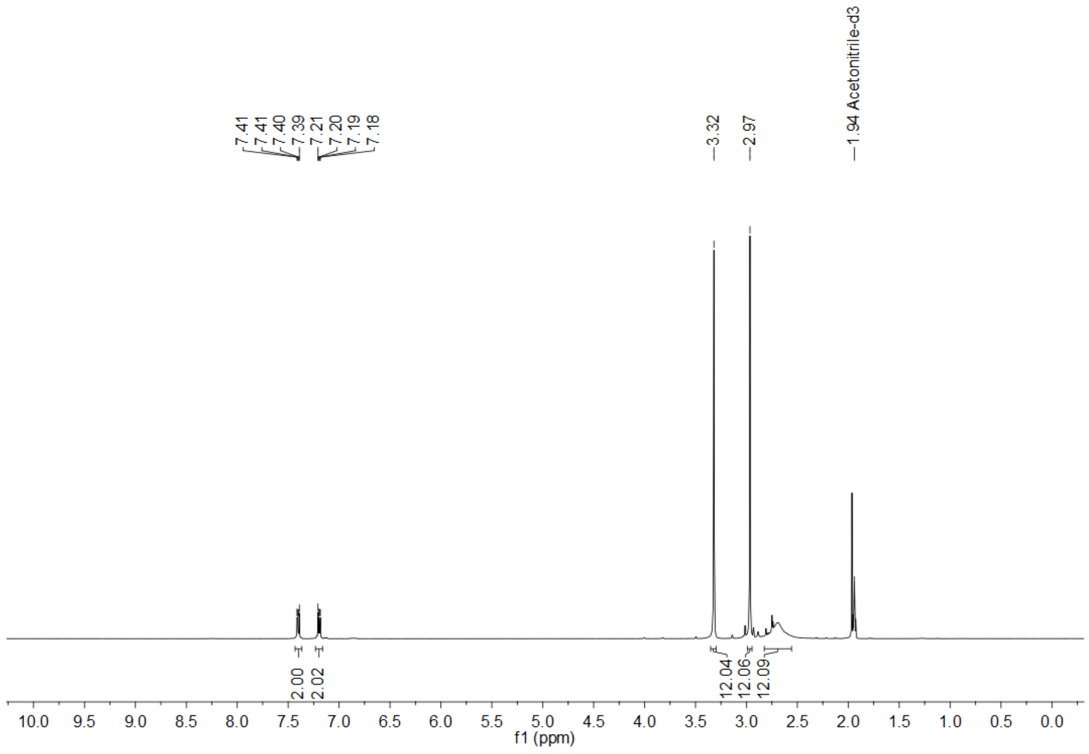

^{13}C NMR spectrum (400 MHz, CD_3CN) for **P4$_{isomer}$**$(GaCl_4)_2$:

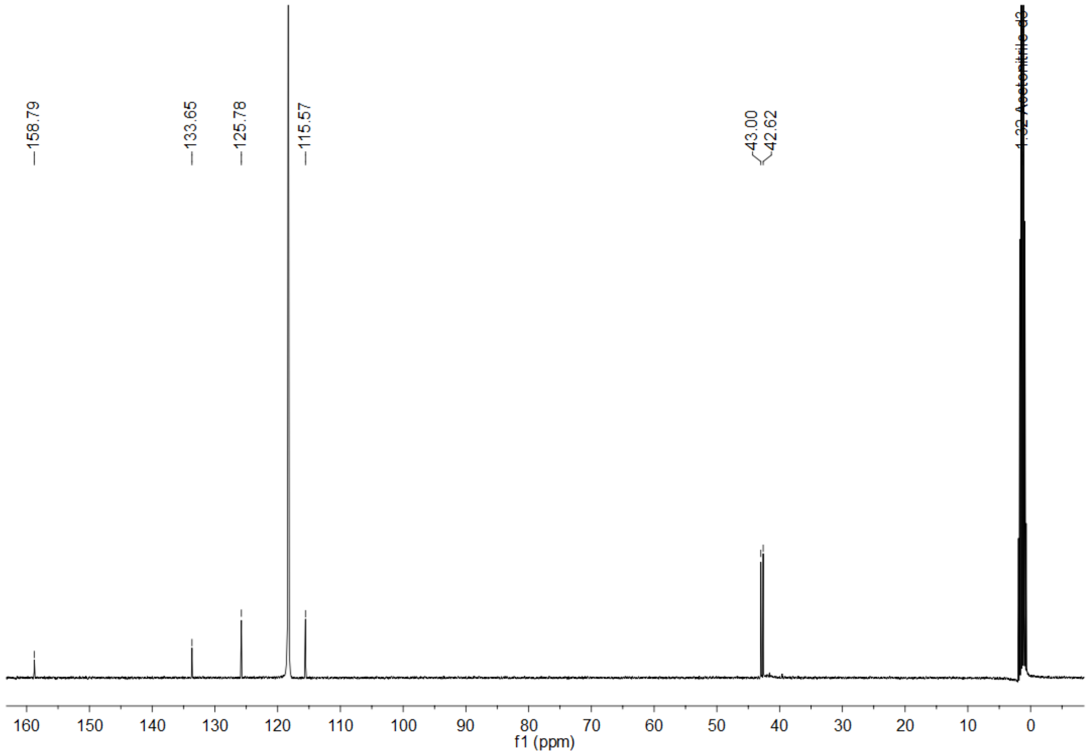

^{11}B NMR spectrum (128 MHz, CD_3CN) for **P4$_{isomer}$**$(GaCl_4)_2$:

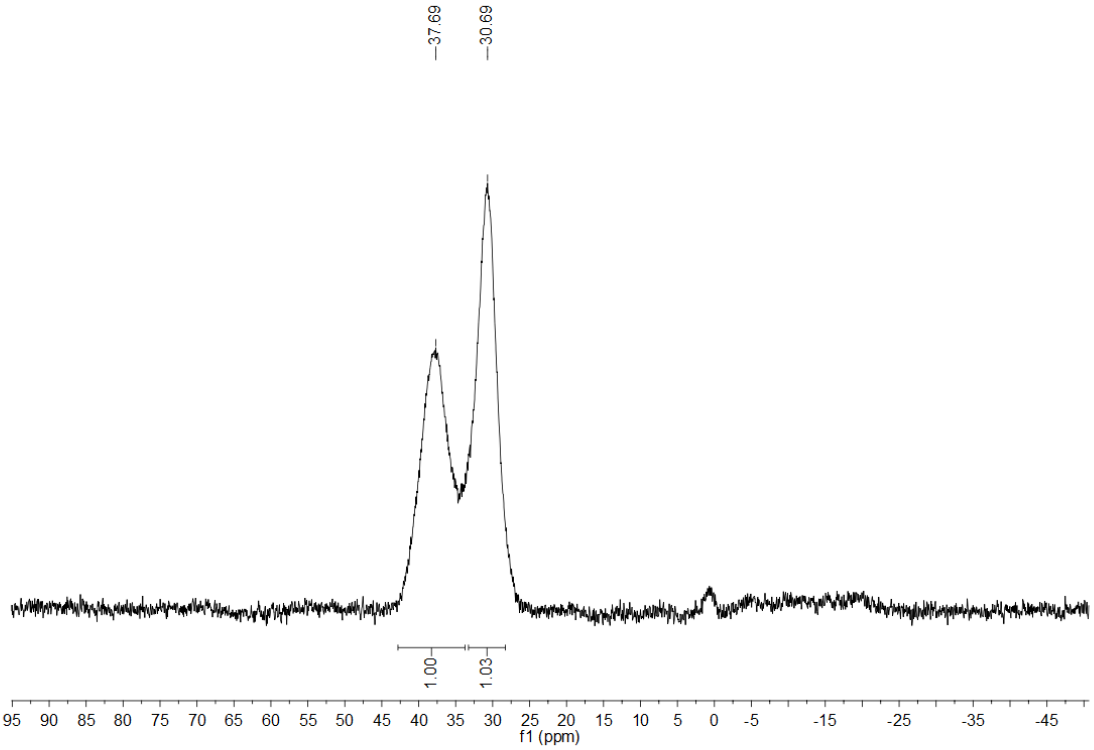

$^{11}B\{^{1}H\}$ NMR spectrum (128 MHz, CD_3CN) for **P4$_{isomer}$**$(GaCl_4)_2$:

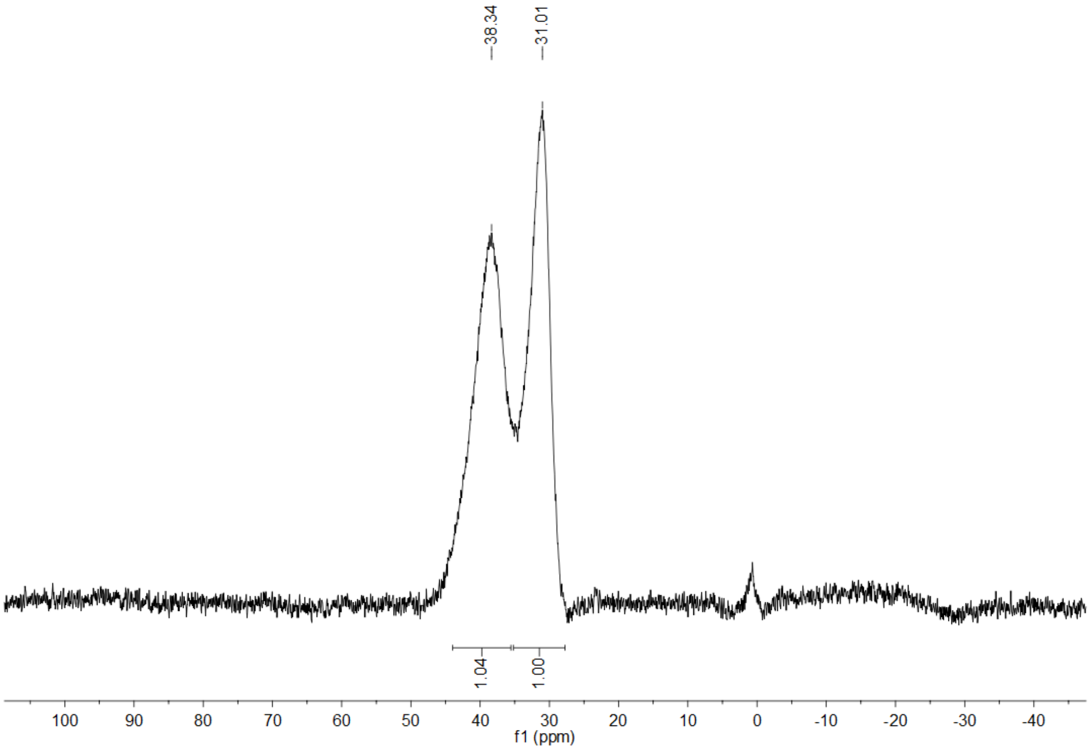

^{1}H NMR spectrum (400 MHz, CD_3CN) for the isomeric mixture of **P4**$(GaCl_4)_2$ and **P4$_{isomer}$** $(GaCl_4)_2$:

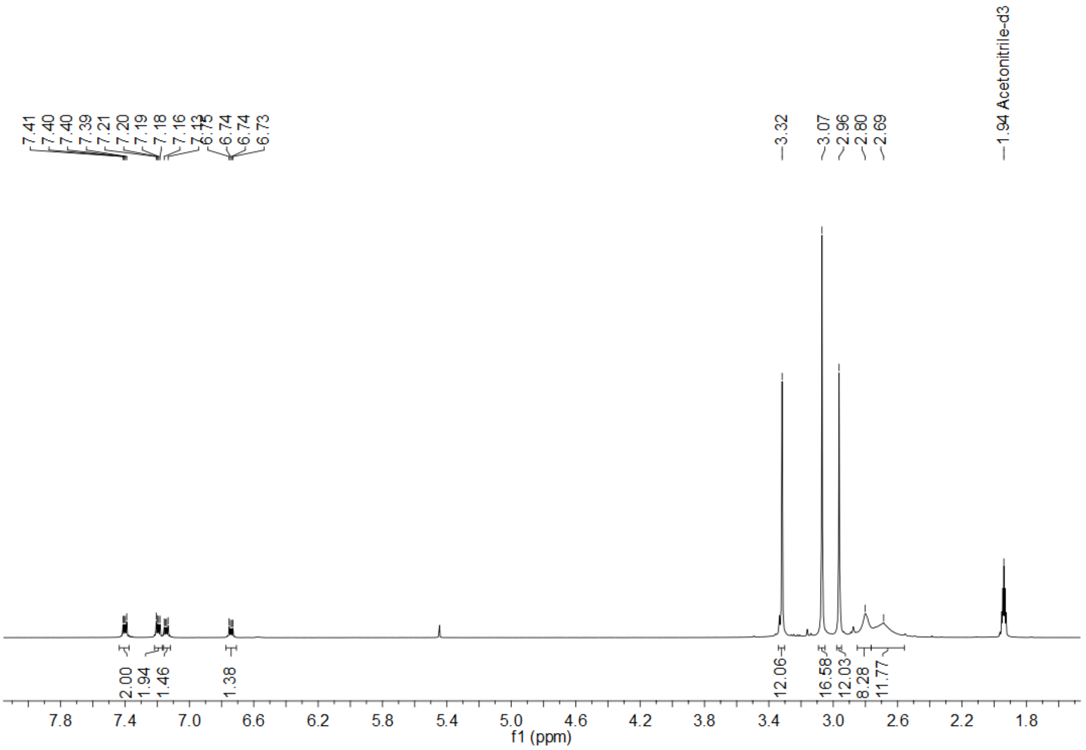

^{1}H NMR spectrum (200 MHz, CD_3CN) for **P4**$(GaCl_4)_2$:

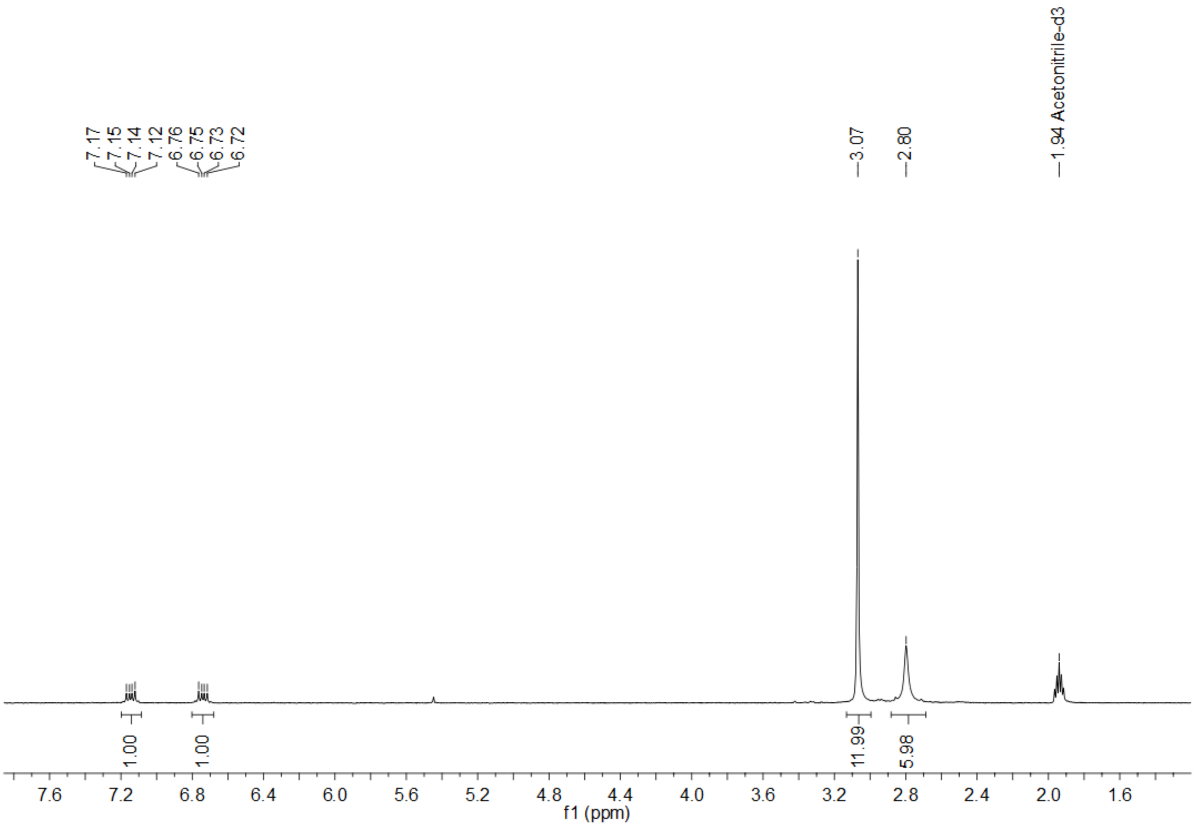

^{13}C NMR spectrum (100 MHz, CD_3CN) for **P4**$(GaCl_4)_2$:

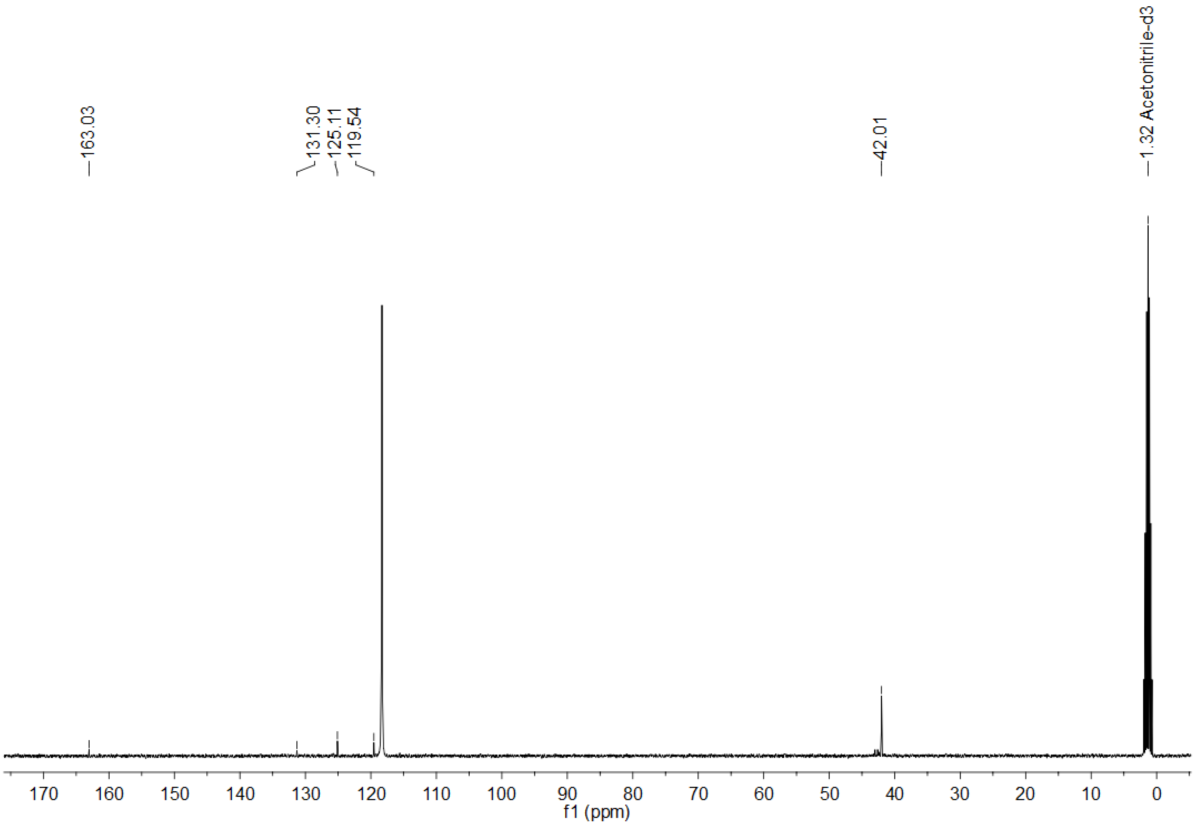

^{1}H NMR spectrum (400 MHz, CD_3CN) for **P4**$(OTf)_2$:

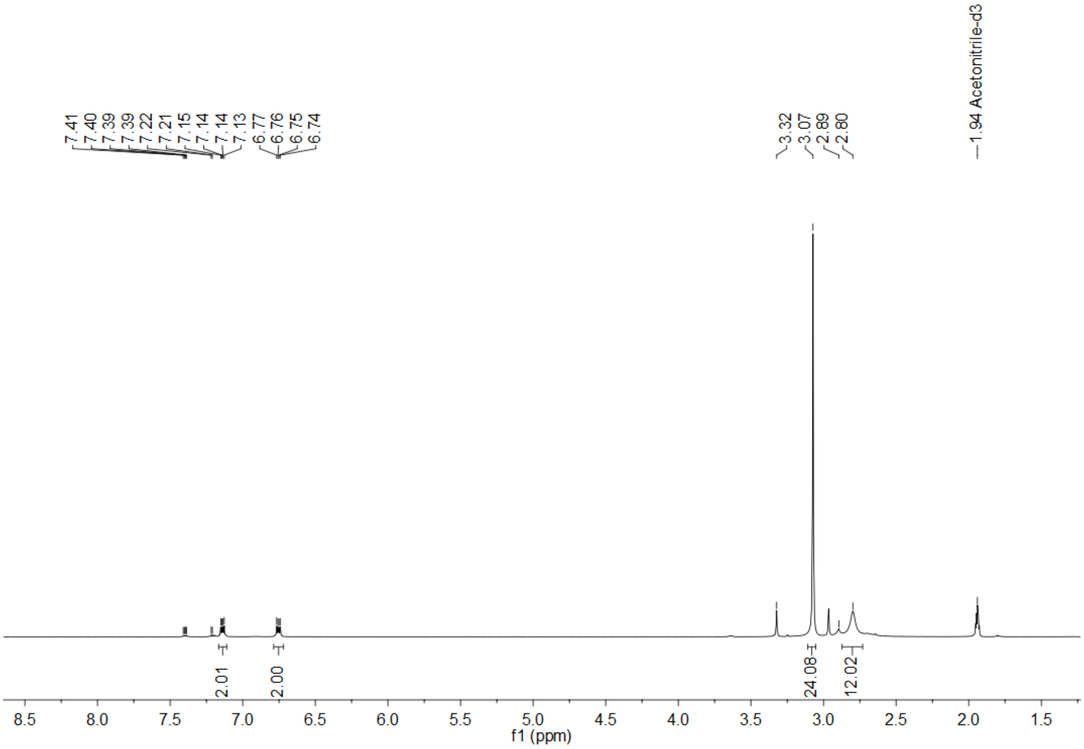

^{13}C NMR spectrum (100 MHz, CD_3CN) for **P4**$(OTf)_2$:

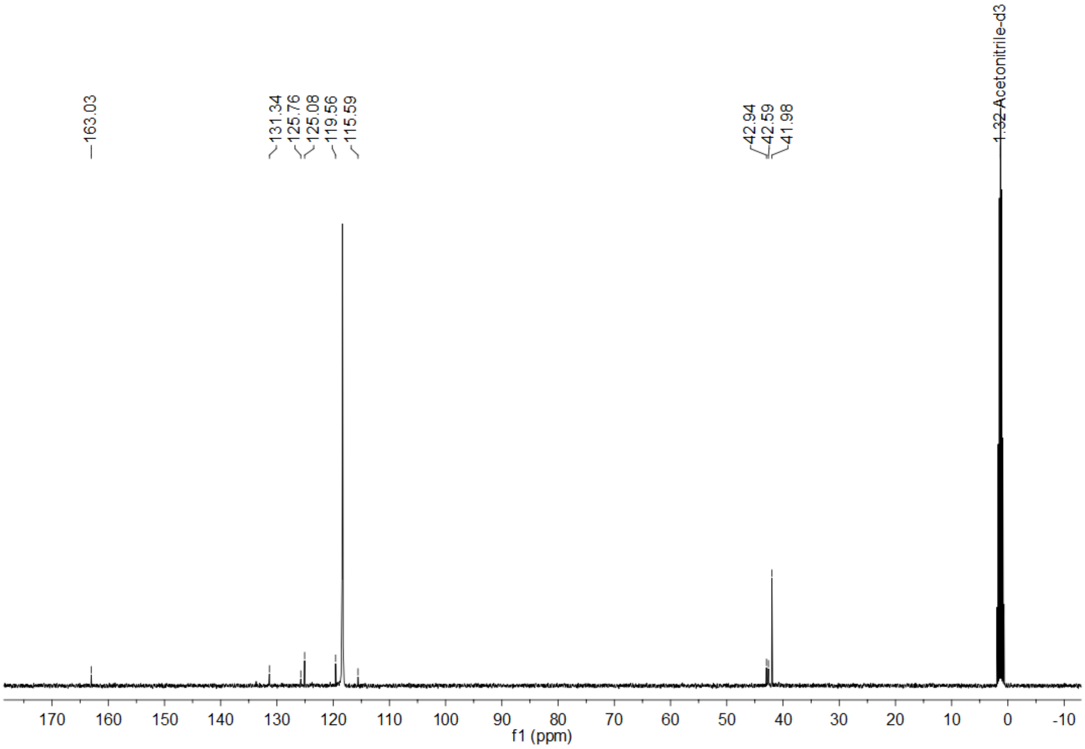

^{11}B NMR spectrum (128 MHz, CD_3CN) for **P4**$(OTf)_2$:

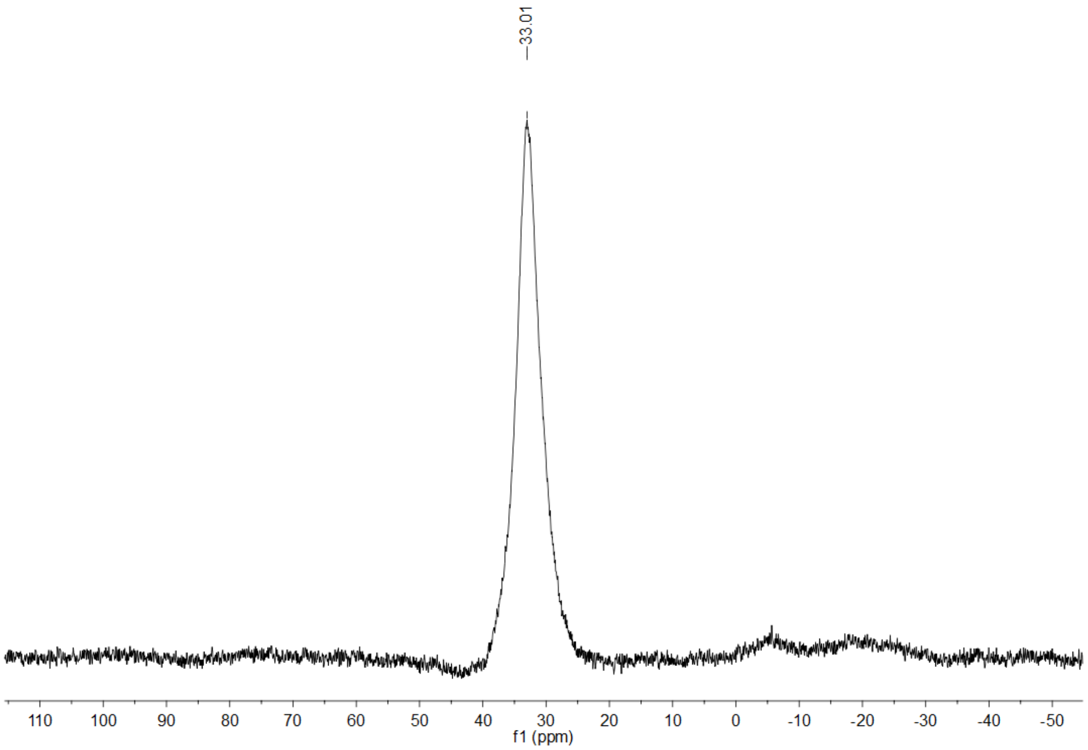

^{1}H NMR spectrum (400 MHz, CD_3CN) for $\mathbf{P4}_{\mathbf{isomer}}(OTf)_2$:

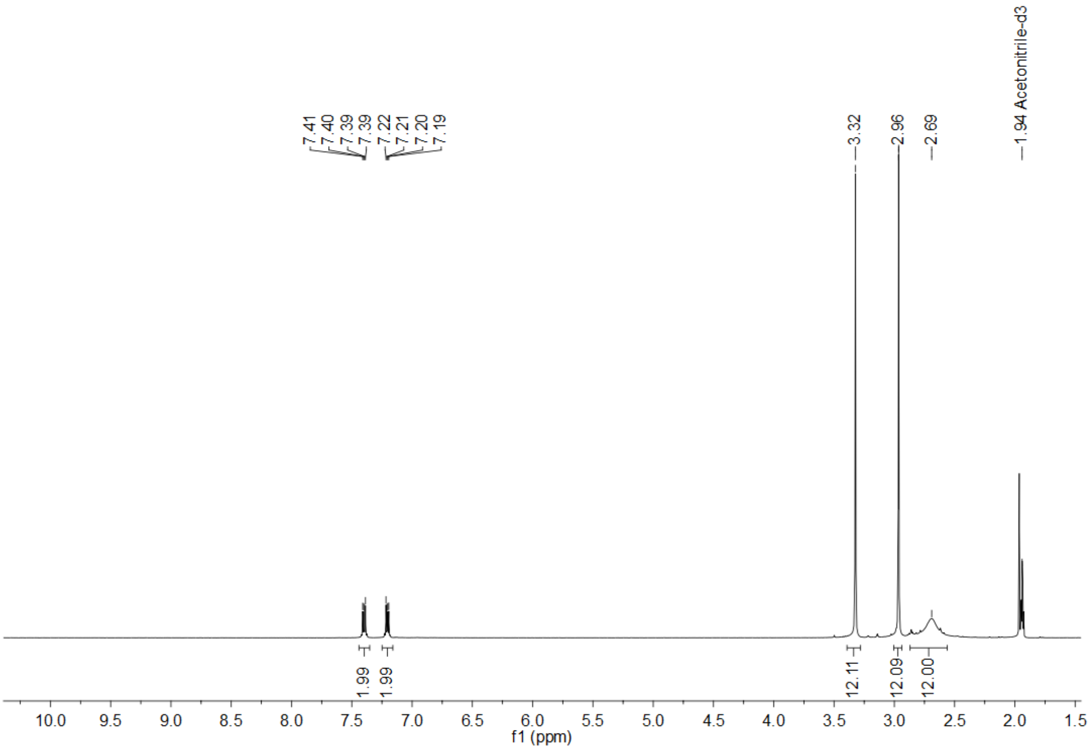

^{13}C NMR spectrum (100 MHz, CD_3CN) for $\mathbf{P4}_{\mathbf{isomer}}(OTf)_2$:

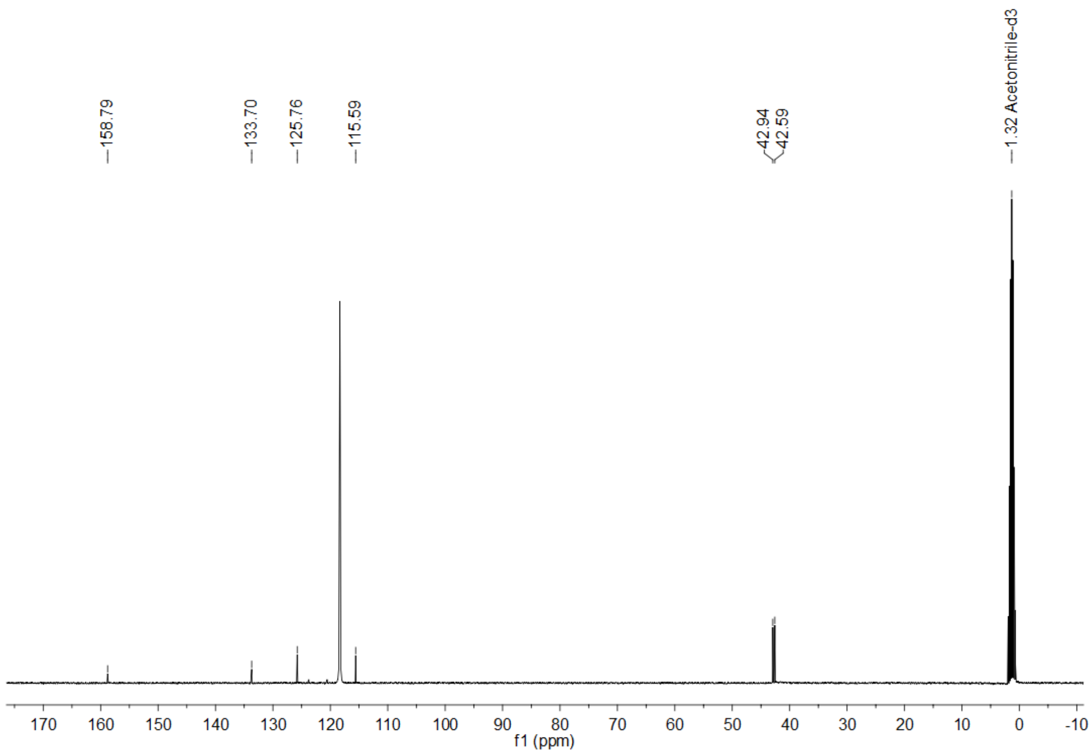

^{11}B NMR spectrum (128 MHz, CD_3CN) for **P4$_{isomer}$**$(OTf)_2$:

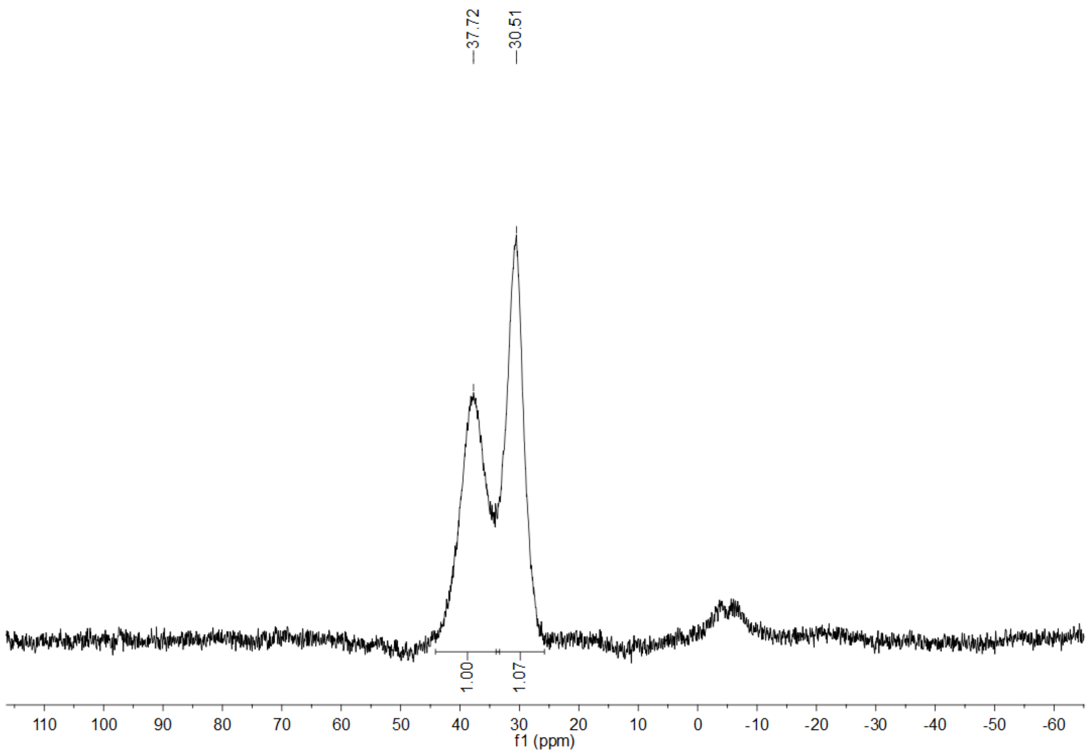

$^{11}B\{^{1}H\}$ NMR spectrum (128 MHz, CD_3CN) for **P4$_{isomer}$**$(OTf)_2$:

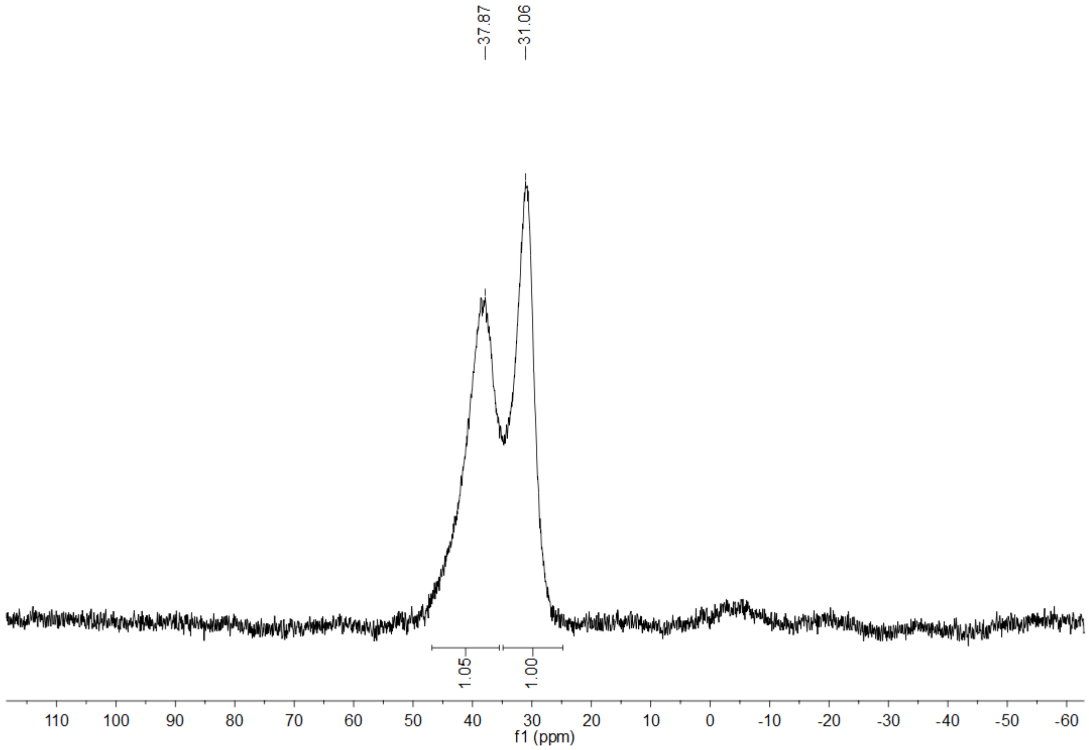

^{1}H NMR spectrum (400 MHz, CD_3CN) for **P4$_{isomer}$F1**$(GaCl_4)_2$:

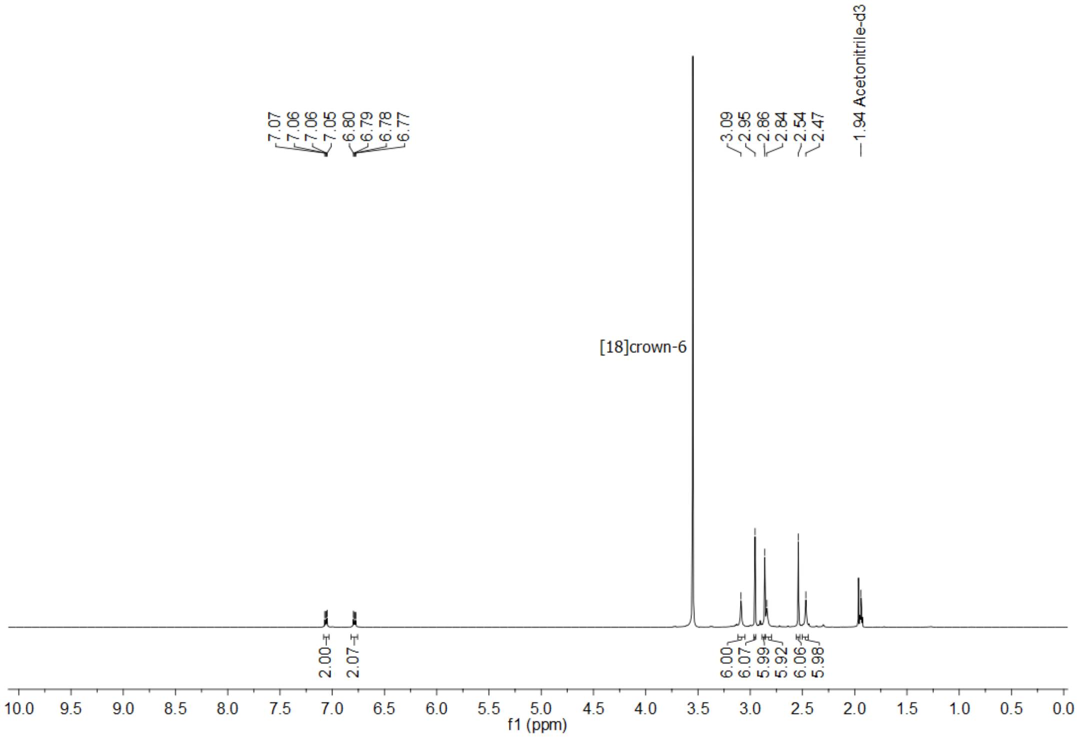

^{11}B NMR spectrum (128 MHz, CD_3CN) for **P4$_{isomer}$F1**($GaCl_4$)$_2$:

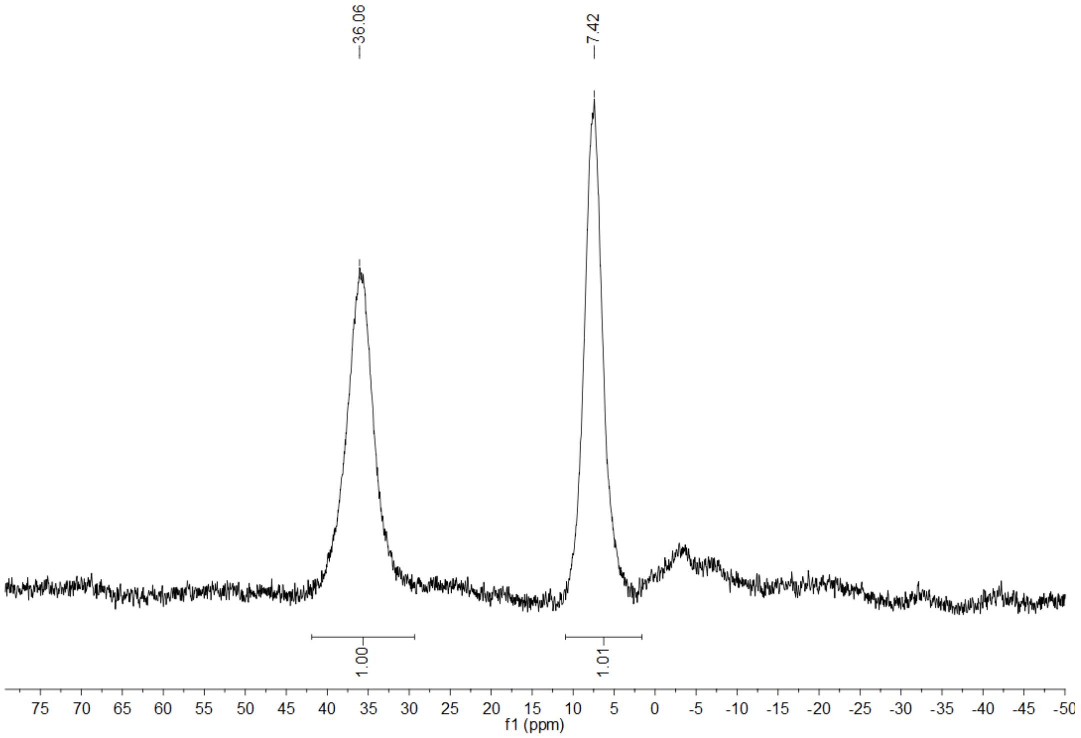

$^{11}B\{^1H\}$ NMR spectrum (128 MHz, CD_3CN) for **P4$_{isomer}$F1**($GaCl_4$)$_2$:

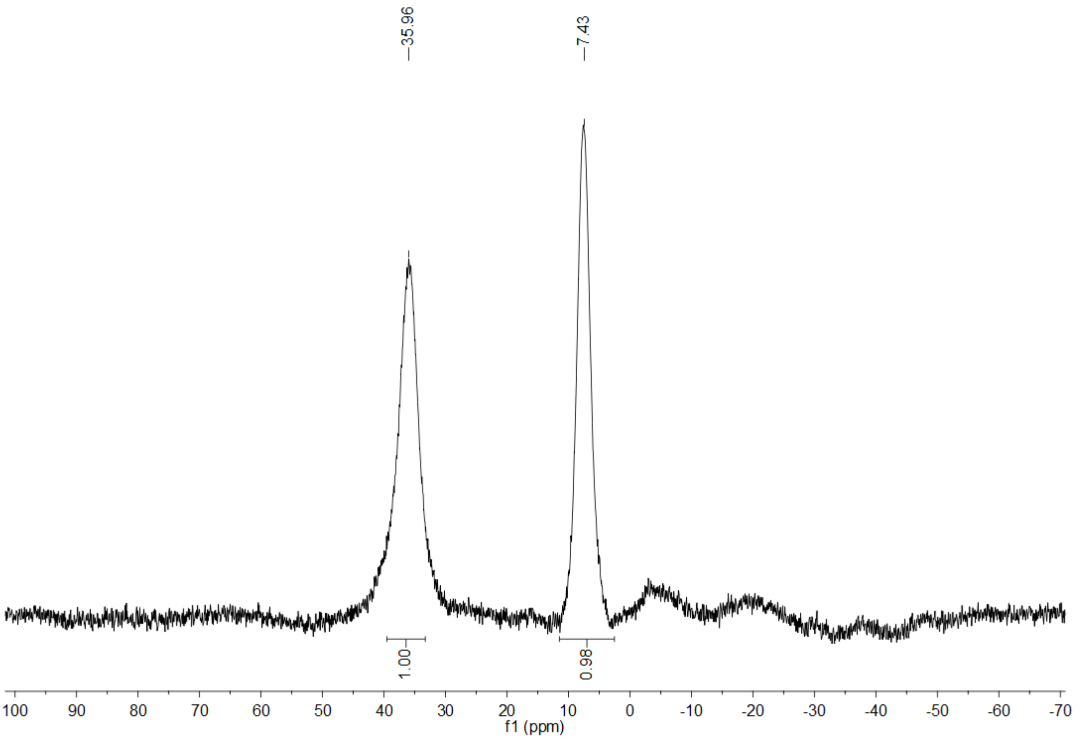

^{19}F NMR spectrum (376 MHz, CD_3CN) for **P4$_{isomer}$F1**$(GaCl_4)_2$:

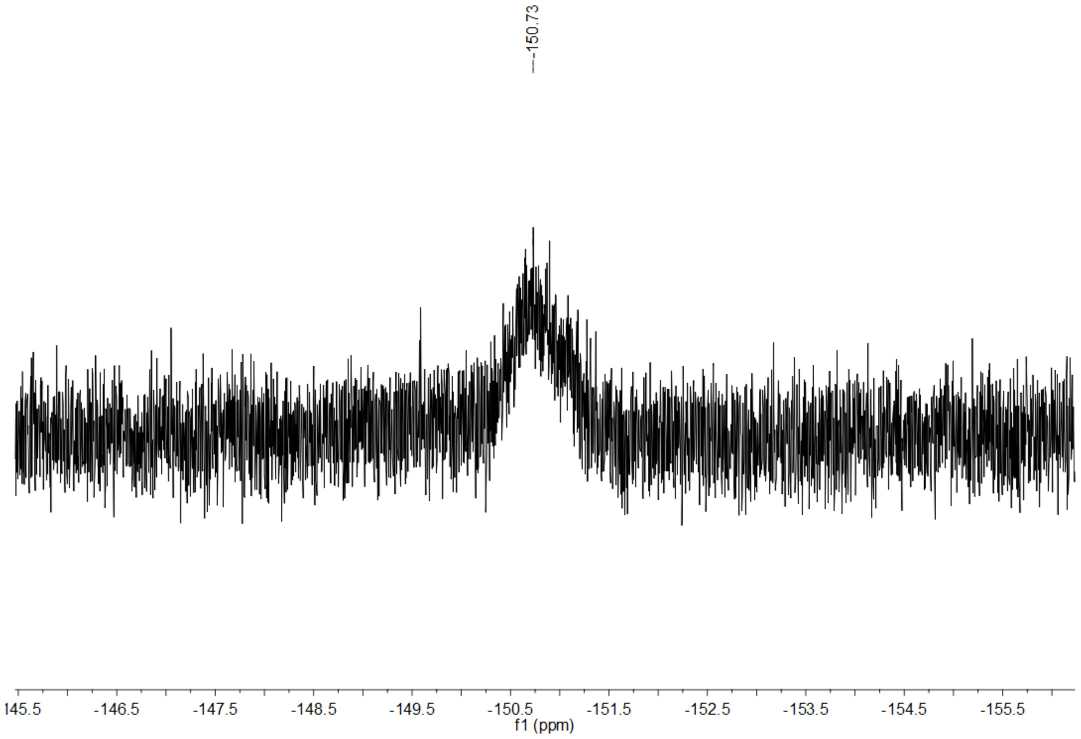

8. References

[S1] D. de Bellefeuille, M. S. Askari, B. Lassalle-Kaiser, Y. Journaux, A. Aukauloo, M. Orio, F. Thomas, X. Ottenwaelder, *Inorg. Chem.* **2012**, *51*, 12796-12804.

[S2] J. Horn, A. Widera, S. Litters, E. Kaifer, H.-J. Himmel, *Dalton Trans.* **2018**, 2009-2017.

[S3] A. Peters, E. Kaifer, H.-J. Himmel, *Eur. J. Org. Chem.* **2008**, 5907–5914.

[S4] M. Kawahata, K. Yamaguchi, T. Ito, T. Ishikawa, *Acta Cryst.* **2006**, *62*, o3301-o3302.

[S5] a) *DENZO-SMN*, Z. Otwinowski & W. Minor, Processing of X-ray Diffraction Data Collected in Oscillation Mode, Methods Enzymol. (1997), 276, Eds C. W. Carter, R. M. Sweet, Academic Press.; b) *SAINT*, Bruker AXS GmbH, Karlsruhe, Germany **2016.**

[S6] a) G. M. Sheldrick, SADABS, Bruker AXS GmbH, Karlsruhe, Germany **2004-2014**; b) L. Krause, R. Herbst-Irmer, G. M. Sheldrick, D. Stalke, J*. Appl. Cryst*. **2015**, *48*, 3.

[S7] a) G. M. Sheldrick, SHELXT, *Program for Crystal Structure Solution*, University of Göttingen, Germany **2014-2018**; b) G. M. Sheldrick, *Acta Cryst*. **2015**, *A71*, 3.

[S8] a) G. M. Sheldrick, *SHELXL-20xx*, University of Göttingen and Bruker AXS GmbH, Karlsruhe,Germany **2012-2018**; b) W. Robinson, G. M. Sheldrick in: N. W. Isaaks, M. R. Taylor (eds.) „*Crystallographic Computing 4*“, Ch. 22, IUCr and Oxford University Press, Oxford, UK, **1988**; c) G. M. Sheldrick, *Acta Cryst*. **2008**, *A64*, 112; (d) G. M. Sheldrick, *Acta Cryst*. **2015**, *C71*, 3.

[S9] O. V. Dolomanov, L. J. Bourhis, R. J. Gildea, J. A. K. Howard, H. Puschmann, OLEX2: A complete structure solution, refinement and analysis program, *J. Appl. Cryst.* **2009**, *42*, 339.

[S10] a) P. v. d. Sluis, A. L. Spek, *Acta Cryst.* **1990**, *A46*, 194; b) A. L. Spek, *Acta Cryst.* **2015**, *C71*, 9.

[S11] a) A. L. Spek, *PLATON*, Utrecht University, The Netherlands; b) A. L. Spek, *J. Appl. Cryst*. **2003**, *36*, 7.

[S12] TURBOMOLE V7.1, 2017, a development of University of Karlsruhe and Forschungszentrum Karlsruhe GmbH, **1989-2007**, TURBOMOLE GmbH, since 2007.

[S13] K. Eichkorn, O. Treutler, H. Öhm, M. Häser and R. Ahlrichs, *Chem. Phys. Lett.* **1995**, *42*, 652–660.

[S14] M. Sierka, A. Hogekamp, R. Ahlrichs, *J. Chem. Phys.* **2003**, *118*, 9136–9148.

[S15] A. D. Becke, *Phys. Rev. A: At., Mol., Opt. Phys.,* **1988**,*38*, 3098−3100.

[S16] C. Lee, W. Yang, R. G. Parr, *Phys. Rev. B: Condens. Matter Mater. Phys.,* **1988**,*37*, 785−789.

[S17] F. Weigend, R. Ahlrichs, *Phys. Chem. Chem. Phys*,. **2005**, *7*, 3297–3305.

[S18] S. Grimme, J. A., S. Ehrlich, H. Krieg, *J. Chem. Phys.* **2010**, *132*, 154104-1–154104-19.

[S19] a) A. Klamt, G. Schüürmann, *J. Chem. Soc.*, *Perkin Trans*. 2 **1993**, 799-805; b) A. Klamt, *WIREs Comput. Mol. Sci.* **2011**, *1*, 699-709.

[S20] A. Wagner, H.-J. Himmel, *J. Chem. Inf. Model* **2017**, *57*, 428-438.

[S21] D. Franz, T. Szilvási, A. Pöthig, F. Deiser, S. Inoue, *Chem. Eur. J.* **2018**, *24*, 4283–4288.

[S22] a) F. Neese, *Interdisc. Rev: Comp. Mol. Sci.* **2012**, *2*, 73-78; b) F. Neese, *Interdisc. Rev: Comp. Mol. Sci.* **2017**, e1327.

[S23] S. Grimme, J. G. Brandenburg, C. Bannwarth, A. Hansen, *J. Chem. Phys.* **2015**, *143*, 054107.

[S24] K. Eichkorn, O. Treutler, H. Öhm, M. Häser, R. Ahlrichs, *Chem. Phys. Lett.* **1995**, *240*, 283-290.

[S25] K. Eichkorn, F. Weigend, O. Treutler, R. Ahlrichs, *Theor. Chem. Acc.* **1997**, *97*, 119-124.

[S26] Y. Zhao, D. G. Truhlar, *J. Phys. Chem. A* **2005**, *109*, 5656-5667.

[S27] a) S. Grimme, S. Ehrlich, L. Goerigk, *J. Comput. Chem.* **2011**, *32*, 1456-1465; b) A. D. Becke, E. R. Johnson, *J. Chem. Phys.* **2005**, *122*, 154104; c) E. R. Johnson, A. D. Becke, *J. Chem. Phys.* **2005**, *123*, 024101.

[S28] a) A. Schäfer, C. Huber, R. Ahlrichs, *J. Chem. Phys.* **1994**, *100*, 5829-5835; b) F. Weigend, R. Ahlrichs, *Phys. Chem. Chem. Phys.* **2005**, *7*, 3297-3305.

[S29] S. Grimme, *Chem. Eur. J.* **2012**, *18*, 9955-9964.

[S30] a) L. O. Müller, D. Himmel, J. Stauffer, G. Steinfeld, J. Slattery, G. Santiso-Quiñones, V. Brecht, I. Krossing, *Angew. Chem. Int. Ed.* **2008**, *47*, 7659-7663; b) H. Böhrer, N. Trapp, D. Himmel, M. Schleep, I. Krossing, *Dalton. Trans.* **2015**, *44*, 7489-7499.

[S31] a) A. Klamt, *J. Phys. Chem.* **1995**, *99*, 2224-2235; b) F. Eckert, A. Klamt, *AIChE Journal* **2002**, *48*, 369-385; c) A. Klamt, B. Mennucci, J. Tomasi, V. Barone, C. Curutchet, M. Orozco, F. J. Luque, *Accounts Chem. Res.* **2009**, *42*, 489-492.

[S32] E. J. Baerends, T. Ziegler, A. J. Atkins, J. Autschbach, D. Bashford, O. Baseggio, A. Brces, F. M. Bickelhaupt, C. Bo, P. M. Boerritger, L. Cavallo, C. Daul, D. P. Chong, D. V. Chulhai, L. Deng, R. M. Dickson, J. M. Dieterich, D. E. Ellis, M. van Faassen, A. Ghysels, A. Giammona, S. J. A. van Gisbergen, A. Goez, A. W. Gtz, S. Gusarov, F. E. Harris, P. van den Hoek, Z. Hu, C. R. Jacob, H. Jacobsen, L. Jensen, L. Joubert, J. W. Kaminski, G. van Kessel, C. Knig, F. Kootstra, A. Kovalenko, M. Krykunov, E. van Lenthe, D. A. McCormack, A. Michalak, M. Mitoraj, S. M. Morton, J. Neugebauer, V. P. Nicu, L. Noodleman, V. P. Osinga, S. Patchkovskii, M. Pavanello, C. A. Peeples, P. H. T. Philipsen, D. Post, C. C. Pye, H. Ramanantoanina, P. Ramos, W. Ravenek, J. I. Rodrguez, P. Ros, R. Rger, P. R. T. Schipper, D. Schlns, H. van Schoot, G. Schreckenbach, J. S. Seldenthuis, M. Seth, J. G. Snijders, Sol, ADF2019, SCM, Theoretical Chemistry, Vrije Universiteit, Amsterdam, The Netherlands, https://www.scm.com.

[S33] E. Van Lenthe, E. J. Baerends, *J. Comput. Chem.* **2003**, *24*, 1142-1156.

[S34] NBO 7.0. E. D. Glendening, J. K. Badenhoop, A. E. Reed, J. E. Carpenter, J. A. Bohmann, C. M. Morales, P. Karafiloglou, C. R. Landis, and F. Weinhold, Theoretical Chemistry Institute, University of Wisconsin, Madison, WI (**2018**)

[S35] T. A. K. AIMAll (Version 17.01.25), TK Gristmill Software, Overland Park KS, USA, **2017** (aim.tkgristmill.com).

[S36] a) R. F. W. Bader, H. Essén, *J. Chem. Phys.* **1984**, *80*, 1943-1960; b) R. F. W. Bader, *Chem. Rev.* **1991**, *91*, 893-928; c) F. Cortés-Guzmán, R. F. W. Bader, *Coord. Chem. Rev.* **2005**, *249*, 633-662; d) D. Stalke *Chem. Eur. J.* **2011**, *17*, 9264-9278.

[S37] D. Cremer, E. Kraka, *Angew. Chem. Int. Ed.* **1984**, *23*, 627-628.

[S38] a) N. Kocher, J. Henn, B. Gostevskii, D. Kost, I. Kalikhman, B. Engels, D. Stalke, *J. Am. Chem. Soc.* **2004**, *126*, 5563-5568; b) R. F. W. Bader, *J. Phys. Chem. A* **1998**, *102*, 7314-7323.

[S39] C. C. Pye, T. Ziegler, *Theor. Chem. Acc.* **1999**, *101*, 396-408.

[S40] a) J. P. Perdew, *Phys. Rev. B* **1986**, *33*, 8822-8824; b) A. D. Becke, *Phys. Rev. A* **1988**, *38*, 3098-3100.

[S41] M. Findeisen,T.Brand,S. Berger,Magn. *Reson.Chem.* **2007**, *45*, 175–178.

[S42] C. Ammann, P. Meier, A. E. Merbach, *J. Magn. Reson.* **1982**, *46*, 319-321.
